# Genome-wide analysis of primary microRNA expression using H3K36me3 ChIP-seq data

**DOI:** 10.1016/j.csbj.2021.03.035

**Published:** 2021-04-05

**Authors:** Tanja Turunen, Ana Hernández de Sande, Petri Pölönen, Merja Heinäniemi

**Affiliations:** a School of Medicine, University of Eastern Finland, Kuopio FI-70200, Finland; b Department of Environmental and Biological Sciences, University of Eastern Finland, Joensuu FI-80101, Finland

**Keywords:** MiRNA, Lineage-determination, Cardiac septal defects, Transcriptional regulation

## Abstract

MicroRNAs are key players in gene regulatory networks controlling cell homeostasis. Their altered expression has been previously linked to disease outcomes and microRNAs thus serve as biomarkers for disease diagnostics. However, their synthesis and its transcriptional regulation have been challenging to investigate. In this study, we validated the use of H3K36me3 histone modification for the quantification of microRNA transcription levels using data from the ENCODE Consortium and then applied this approach to provide new insight into the cell-type-specific regulation in tissues, cell line models and cardiac disease. In cardiomyocytes derived from patients suffering from septal defects, carrying a G296S mutation in the transcription factor GATA4, we show that microRNA gene transcription is altered in cardiomyocytes carrying this mutation and coincides with novel super-enhancers formed within regulatory domains defined using chromatin interaction profiles. The most prominently elevated primary transcript encodes for let-7a and miR-100 that may target genes in the Hippo signaling pathway. Collectively, our work presents a methodology to quantify microRNA gene expression using histone marker data and paves the way for functional studies of cell-type-specific transcriptional regulation occurring in disease pathology.

## Highlights

• Expression of microRNA genes (pri-miRNA) can be quantified using NGS methodology from H3K36me3 ChIP-seq data, a histone marker that is deposited during elongation.
• Cell-specific analysis of pri-miRNAs TSS activity complements tissue miRnomes to uncover disease-relevant changes.
• Mutation in the cardiac lineage transcription factor GATA4 altered super-enhancer landscape within pri-miRNA loci.
• Pri-miRNA transcript encoding for let-7a and miR-100 mature forms is prominently up-regulated in patients carrying the GATA4 mutation.

## Graphical abstract

**Figure:**
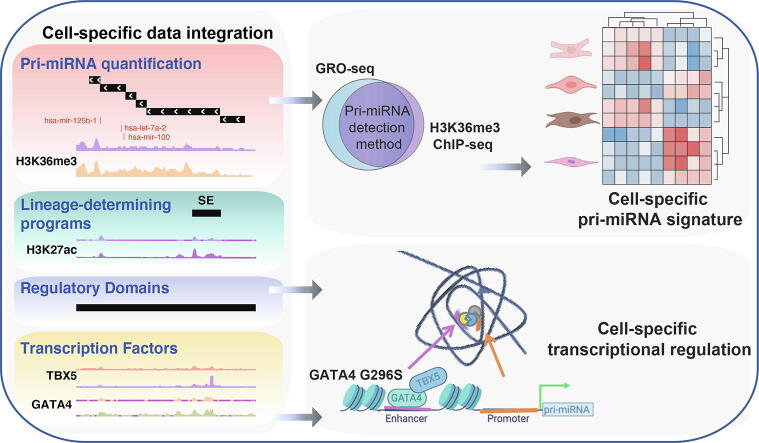

## 1 Introduction

Mature microRNAs (miRNA) are endogenous 20–22 nt long non-coding RNAs that post-transcriptionally regulate gene expression. Thus, miRNA-mediated regulation has an impact on diverse cellular processes, including cell development, stress responses, and disease progression [1]. However, studies on the regulation of miRNA genes (primary miRNAs, pri-miRNA) have not been widely carried out. Pri-miRNAs are transcribed and rapidly processed into shorter molecules (reviewed by [2]) and in consequence, conventional methods such as RNA-seq fail to capture their transcriptional dynamics. This challenge also hindered the annotation of pri-miRNA transcripts from a single genomic readout. In our previous work [3], we have integrated multiple genomics data types (global-run on sequencing (GRO-seq), cap analysis gene expression (CAGE) sequencing, and chromatin immunoprecipitation sequencing (ChIP-seq)) from 27 human cell lines to annotate pri-miRNA alternative transcription start sites (TSSs) and to assign the transcribed regions at these non-coding gene loci based on the nascent transcript signal. Our study provided a bioinformatics workflow that utilized genomic annotations (referred to as TSS elements) corresponding to over 1,500 pri-miRNA TSSs to compare their activities based on a signal subtraction approach. We showed that multiple TSSs contribute to the transcription of these non-coding RNAs in a cell- and stimulus-specific manner. This quantification approach, with the obtained pri-miRNA coordinates, is directly applicable to quantify their transcription level from nascent transcriptome data. However, isolation of nascent transcripts for sequencing is not widely adopted, prompting the comparison of alternative data types that could be suitable to study pri-miRNA transcription.

Previous studies have shown that distinct histone modifications that can be studied by the widely used ChIP-seq assays are enriched at chromatin regions corresponding to a specific functional state [4], [5]. Among activity markers, histone 3 lysine 27 acetylation (H3K27ac) is enriched at active regulatory regions, histone 3 lysine 4 trimethylation (H3K4me3) at active TSSs, and histone 3 lysine 36 trimethylation (H3K36me3) modification is enriched in regions with active transcript elongation. On the contrary, histone modifications such as histone 3 lysine 27 trimethylation (H3K27me3) and H2A.Z associate with gene silencing [4]. Thus, histone modifications can serve as a dynamic readout of regulatory regions and transcriptional activity (H3K36me3) to characterize gene and pri-miRNA regulation [6].

Congenital heart defects (CHD) are the most prevalent type of malformations present at birth in humans. CHD pathology manifests with alterations in the regulation of gene expression during cardiac development. Genetic alterations in key transcription factors (TF) driving cardiac development, including GATA4, TBX5, MEF2A, and NKX2-5, have been associated with CHD development and outcome [7], [8], [9], [10]. Furthermore, non-coding RNAs that include miRNAs are important contributors to the cardiac regulatory network [11]. Previous work to dissect in detail the contribution of miRNA-mediated regulation in CHD has identified miRNA biomarkers of the disease state [12], [13], [14]. However, tissue profiling of heart, plasma or serum fails to identify the cell type that synthesizes these miRNAs. Therefore, cardiac lineage-specific regulation of miRNAs and its impact on the disease outcome has been difficult to study.

Integrating TF binding and chromatin dynamics profiles at distal regulatory elements (enhancers) with gene expression data enables studying transcriptional regulation in disease. Moreover, groups of enhancers referred to as super-enhancers (SE) facilitate the described (lineage-specific) regulation by participating in the regulation of key genes involved in the cellular control at the specific developmental state [15]. SEs can be distinguished from typical enhancers since they present with wider H2K27ac histone modification regions that enable their detection. Furthermore, regulatory pathways and gene expression profiles can be associated using statistical analysis with specific signaling pathways to provide mechanistic insight into the establishment of disease phenotypes and to inform candidate drug targeting approaches.

Here, we evaluated the suitability of different histone modifications as a novel approach for quantification of pri-miRNA expression levels in cells using ChIP-seq data. We show that our approach enables assessing the contribution of cell-type-specific pri-miRNA expression in tissue miRnomes and across cell line models based on H3K36me3 histone marker. To provide new insights into pri-miRNA regulation, we analyzed differentially expressed (DE) pri-miRNA loci from induced pluripotent stem (iPS) cells-derived cardiomyocytes (referred to as iCM) carrying a missense mutation in GATA4 [16], revealing changes in the activities of these non-coding gene loci and candidate target pathways downstream GATA4, TBX5, and SEs alterations in disease [16].

## 2 Results

### 2.1 H3K36me3 histone modification level captures genome-wide pri-miRNA expression and differential TSS utilization

**Fig. 1:**
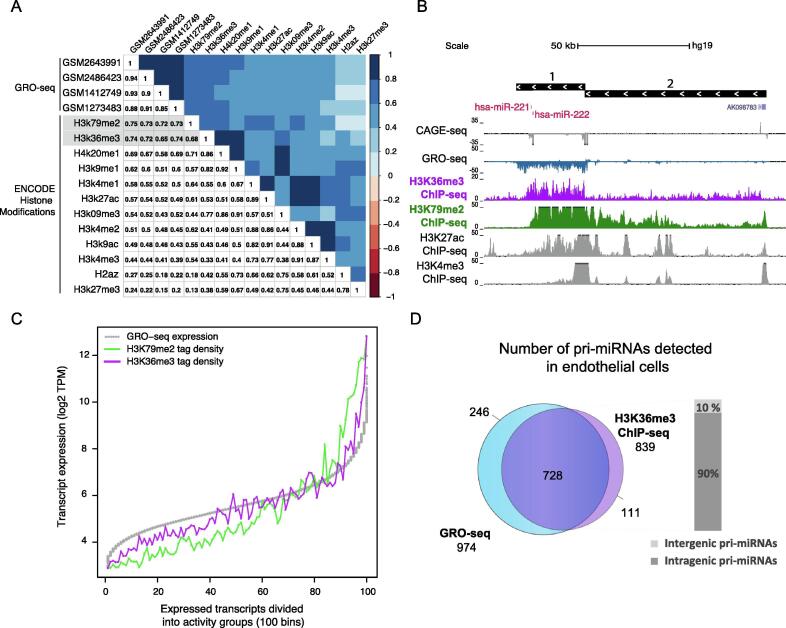
**Genome-wide quantification of primary transcripts using transcriptomic and epigenetic data in human endothelial cells.** (A) Pearson correlation of primary transcript expression (counts per million, CPM) quantified from GRO-seq and histone ChIP-seq data from endothelial cells (blue tones correspond to positive correlation whereas red tones correspond to negative correlation from 1 to −1). Best correlating histone marks are highlighted in grey. (B) Signal profiles indicative of active transcription and chromatin state are visualized at the hsa-mir-221~222 locus (chrX:45,584,514–45,719,179) as follows: annotation tracks indicate the region used for quantification of output from each pri-miRNA TSS (black box with arrows pointing left as hsa-mir-221~222 is transcribed from the minus strand) and mature miRNAs (red). NGS hubs correspond to CAGE-seq, GRO-seq, and ChIP-seq tracks for H3K36me3, H3K79me2, H3K27ac, and H3K4me3. (C) Transcripts were separated into 100 bins according to their mean expression levels based on GRO-seq data (log2 transformed transcripts per million (TPM), grey line). Histone marker average tag density (H3K36me3 purple and H3K79me2 green, y-axis) was plotted for each bin (group 1 has transcripts with lowest average expression; group 100 with highest, x-axis). (D) Pri-miRNAs detected based on either GRO-seq (blue) or ChIP-seq data (purple) are compared in a Venn diagram. Proportions of intergenic (light grey) and intragenic (dark grey) pri-miRNAs detected are shown in a barplot. (For interpretation of the references to color in this figure legend, the reader is referred to the web version of this article.)

Pri-miRNA expression can be efficiently captured by nascent transcriptomes (e.g. GRO-seq methodology); however, this type of data is not widely available. To find a new approach to quantify pri-miRNA expression, we evaluated whether different histone modifications measured using ChIP-seq could be utilized similarly. Towards this end, we used the TSS element coordinates introduced in [3] (genomic annotations that start for each transcript at its TSS and continue either until the next active TSS, or until the end of the transcript) for genome-wide quantification and correlated the signal level (TSS activity, see Methods) from GRO-seq (N = 4) and ChIP-seq data across 12 different histone modifications collected by the ENCODE project (ENCODE consortium accession numbers: Table S1) [17], [18]. Using human umbilical vein endothelial cells (HUVEC, referred hereafter as endothelial cells) for the benchmark, we found that H3K36me3 and H3K79me2 levels had the highest overall correlation with GRO-seq-based quantification (Pearson correlation coefficient from 0.65 to 0.74 for H3K36me3 and from 0.72 to 0.75 for H3K79me2) (Fig. 1A). To support the findings presented, we additionally examined H3K36me3 and H3K79me2 correlations in A549 (lung cancer), GM cell lines (lymphoblastoid), IMR-90 (fibroblast), and K562 (myeloid cancer), resulting in similar correlations values as shown for endothelial cells (Fig. S1A). To illustrate the coverage of ChIP-seq signal profiles of the different histone modifications across the gene body, we visualized GRO-seq and different histone mark levels at the hsa-mir-221~222 locus (this pri-miRNA gene locus encodes for both hsa-miR-221 and hsa-miR-222, hereafter referred with ~ between pri-miRNAs). Two TSSs are active, and their additive contribution can be evaluated based on quantifying the signal level within non-overlapping regions indicated by black rectangles in Fig. 1B (refer to Methods). We further compared H3K36me3 profiles across multiple cell types at the hsa-mir-29a~29b-1 locus (as previously shown in [3], this locus has multiple TSSs with cell-type-specific activity, Fig. S2A). Both H3K36me3 and H3K79me2 histone markers are deposited on the gene body, coinciding with GRO-seq signal (Fig. 1B, Fig. S2A-B). The H3K79me2 histone mark was elevated near the TSS, whereas H3K36me3 ChIP-seq signal more equally covered the entire pri-miRNA transcript. As an exception, we noticed that some miRNA host genes with long first introns showed a signal elevation for H3K36me3 from exon 2 (Fig. S2B) that may affect estimating the pri-miRNA level (see also Methods). As the main difference, strand-specificity of GRO-seq signal cannot be captured from the histone marker data and overall, a higher background is present in ChIP-seq signal (yellow box, Fig. S2C). As a second caveat, in total 3% of intragenic and 10% of intergenic pri-miRNA transcript regions overlapped (at least half of the transcript body) with an expressed gene(s) on the opposite strand, which could lead to overestimation of pri-miRNA levels. Despite these differences, pri-miRNA quantification was highly comparable to GRO-seq data and sensitive to capture cell-type-specific expression between the cell lines analyzed.

To ascertain whether levels of H3K79me2 and H3K36me3 histone modification correlate with transcriptional activity, i.e. whether coinciding profiles of these histone modifications are also reflected in corresponding ranking of expression level, we followed the genome-wide approach proposed in [4]. We separated 9,053 expressed transcripts (including annotated Refseq genes and pri-miRNAs) into 100 different transcriptional activity bins based on their mean expression across GRO-seq samples. The mean histone marker level of each bin is shown from the lowest to the highest group (left to right) in Fig. 1C. The level of H3K36me3 histone modification (purple line, Fig. 1C) had the smallest deviation from the expression level quantified from GRO-seq (grey line) and the difference was most distinct at the bins corresponding to low expression level (bins 0–50). The signal from H3K79me2 underestimated the expression of low expressed transcripts and this effect was reproduced in A549, GM cell, IMR-90, and K562 cell lines (Fig. S1, S3A).

Therefore, we concentrated on the use of H3K36me3 ChIP-seq data in the quantification of pri-miRNA transcriptional activity. From 974 pri-miRNA transcripts detected by GRO-seq in endothelial cells, 728 (75%) were also expressed based on H3K36me3 ChIP-seq data of which 656 (90%) were intragenic and 72 (10%) intergenic (Fig. 1D). We next evaluated the correspondence with mature miRNA levels by comparing the summed pri-miRNA TSS activity per locus binned by the level of H3K36me3 histone marker (921 loci, Fig. S3B-C, see also Methods). The processing efficiency (estimated based on detection rate, Fig. S3B) to mature miRNA increased from 3 to 20% (small RNA-seq) and 16–38% (microarray) in the lowest five bins to 13–50% (small RNA-seq) and 23–90% (microarray) in the five highest bins. Examining the detected mature miRNA (violin plots in Fig. S3C, left for microarray and right for small RNA-seq) we also could distinguish an increasing trend in mature miRNA levels for highly expressed pri-miRNAs. Taken together, the H3K36me3 histone marker level corresponds well with primary transcript synthesis (GRO-seq), while the processing efficiency additionally contributes to the resulting mature miRNA level.

### 2.2 H3K36me3-based quantification of pri-miRNA expression allows assessing cell-type-specific contribution in tissue profiles

**Fig. 2:**
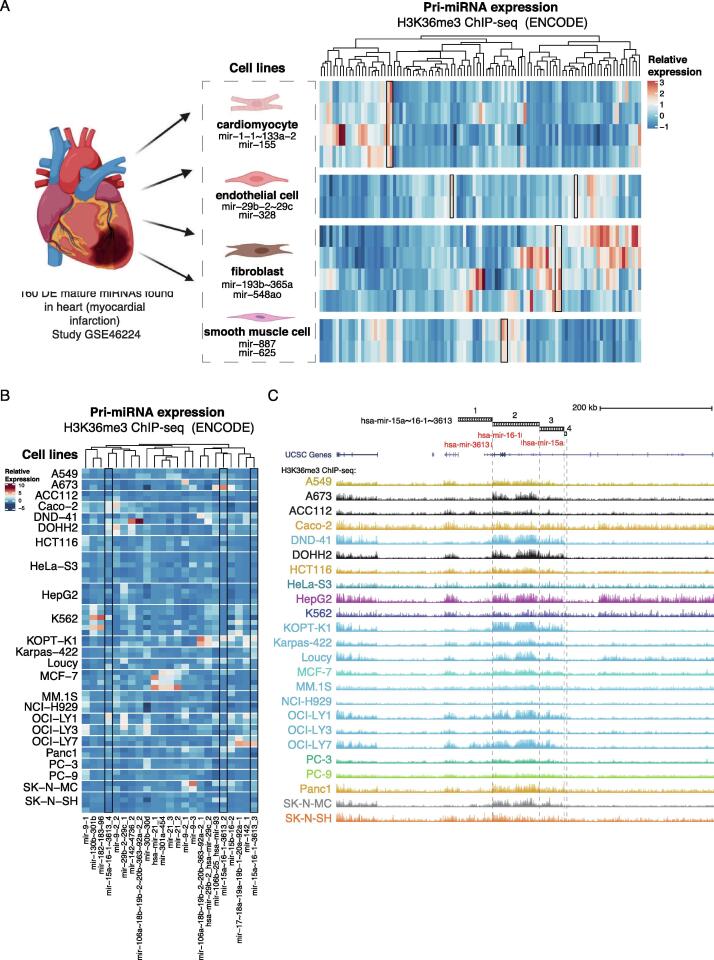
**Application of H3K36me3-based quantification in case studies.** (A) Pri-miRNA expression corresponding to mature miRNAs that differ in expression between normal heart and heart after myocardial infarction were quantified in cardiomyocytes, endothelial cells, fibroblast and smooth muscle cells H3K36me3 ChIP-seq data. RPKM values are row-scaled to show relative TSS activity across samples (red tones correspond to a high level, blue to low level). (B) Transcriptional activity of cancer-related candidate pri-miRNAs clustered based on different TSS activity across cancer cell lines provided by the ENCODE project. The pri-miRNAs shown were selected based on a list of 17 significant mature miRNAs that recurrently negatively correlated with their target (gene)s across TCGA cancer types presented in [22]. RPKM values are row-scaled and denoted from high to low in red and blue colors, respectively. (C) Annotation tracks for pri-miRNAs (black boxes), mature miRNAs (red) and UCSC genes (purple) followed by signal profiles of H3K36me3 cancer cell lines visualized at the hsa-mir-15a~16–1~3613 locus (chr13:50,570,551–50,570,637). (For interpretation of the references to color in this figure legend, the reader is referred to the web version of this article.)

The ENCODE project generated a resource of ChIP-seq across multiple human cell types, to enable functional studies of the non-coding genome. As a first application to leverage our H3K36me3 ChIP-seq based quantification of pri-miRNA locus activities, we focused on comparing their transcription level across cell types in a given tissue context. We selected data from the heart tissue where key tissue-resident cell types include cardiomyocytes, smooth muscle cells, endothelial cells and fibroblasts. We selected miRNAs that change their expression level before and after myocardial infarction (GEO accession number: GSE46224, 160 DE mature miRNAs [19]) and retrieved their genomic coordinates (considering that some mature miRNA are encoded from multiple gene loci). We then matched these miRNAs by genomic coordinate overlap with the corresponding pri-miRNA transcript variants and quantified their expression levels in cell types that are present in the cardiac tissue (ENCODE consortium accession numbers: Table S1). In total, 100/150 pri-miRNA transcripts were detected across the cell types studied (Fig. 2, refer to Table S2 for pri-miRNA annotation in panel A). Clustering of the pri-miRNA expression levels revealed that each cell type could differentially contribute to the heart tissue miRnome. Notably, the highest expression in cardiomyocytes characterized only a quarter of the analyzed miRNA genes and many of these disease-altered miRNAs had the highest level in fibroblasts (Fig. 2A). Cardiomyocyte miRNAs included the hsa-mir-1~133a-2 cluster and hsa-mir-155. Endothelial cells expressed at high levels the hsa-mir-29b-2~29c-2 cluster and mir-328, while hsa-mir-887 and hsa-mir-625 were distinctive of smooth muscle cells, in agreement with FANTOM5 [20], [21]. Our analysis highlighted two pri-miRNA loci, hsa-mir-193b~365a and mir-548ao, to correspond to the most abundant disease-associated miRNA in fibroblasts. Overall, our analysis underscores the cell type diversity in miRNA gene transcriptional activities and the value of integrating the ENCODE resource into miRnome analysis to identify cell-type-specific contributions of the non-coding genome in disease.

ENCODE has also become a unique resource for cancer genomics, with the potential to systematically characterize transcriptional and post-transcriptional regulatory networks. Integrating this rich resource with patient profiles can inform the selection of appropriate cell line models for further functional studies [22]. We selected as an example 17 miRNAs that were previously shown to recurrently negatively correlate with cancer gene signatures across TCGA cancer types [23]. Similarly as above, we retrieved the corresponding pri-miRNA TSS elements and quantified pri-miRNA expression across cancer cell lines based on H3K36me3 ChIP-seq data. Cell lines clustered according to the pri-miRNA TSS activities, and we observed that both the basal transcriptional activity of pri-miRNA genes and their TSS utilization varied between cancer cell lines. This finding demonstrated that not all cancer cell lines shown have the same pri-miRNAs transcribed from the same TSS (Fig. 2B-C), suggesting that they are wired into distinct TF-regulatory networks. Using hsa-mir-15a~16–1~3613 with multiple TSSs as an example, these differential TSS utilization and transcriptional activities can be compared across cell lines (Fig. 2B-C). Overall, TSS2 of hsa-mir-15a~16–1~3613 shows the highest transcriptional activity. In the A673 cell line, the sharp increase in H3K36me3 signal at TSS2 identifies it as the main TSS used. In comparison, the upstream TSS3 of hsa-mir-15a~16–1~3613 shows the highest activity in DND-41, KOPT-K1 and OCI-LY7 lymphoid cell lines. In summary, our approach extends the current methodology to investigate the functional non-coding regions, providing a resource for the comparison of different pri-miRNA TSS activities across the entire ENCODE collection of H3K36me3 ChIP-seq and complementary other histone marker profiles produced by the ENCODE consortium.

### 2.3 Dysregulation of pri-miRNA expression in a cardiomyocyte disease model

**Fig. 3:**
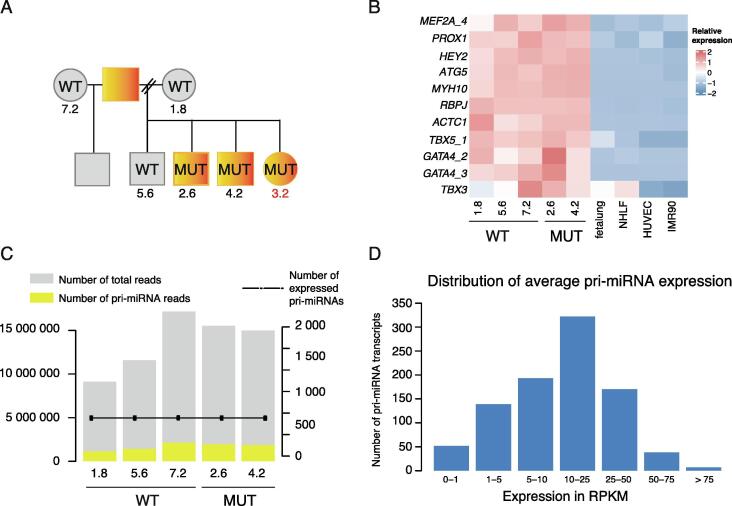
**iCM dataset allows the characterization of cardiac genes and pri-miRNA expression based on H3K36me3 profiles.** (A) Pedigree describing H3K36me3 profiles collected from a family where half of the individuals are carrying GATA4 mutation [16] referred to by sample numbering. Circles indicate females and squares males. All individuals carrying the mutation (MUT) have been diagnosed with septal defects. Healthy individuals are indicated with WT, wild-type familial control. MUT, *GATA4* G296S mutants. (B) Relative expression of heart development associated genes based on H3K36me3 levels in iCM compared to other cell lines. TSS activity values are row-scaled to show relative expression across samples (red tones correspond to a high level, blue to low level). (C) Library sizes (total number of reads, in grey) and the portion of reads that map to pri-miRNAs (in yellow) are shown as overlaid barplots. The y-axis on the left indicates the number of reads. The dotted line and the axis on the right indicate the number of pri-miRNAs detected enriched over the input signal per sample. (D) Distribution of the RPKM values of expressed pri-miRNAs in control iCM cells. (For interpretation of the references to color in this figure legend, the reader is referred to the web version of this article.)

The largest public repository of genome-wide data, NCBI GEO [24], [25], currently hosts 665 human H3K36me3 ChIP-seq samples across different biological conditions that includes data produced by the ENCODE consortium (Table S3). We selected for a case study ChIP-seq profiles from a dataset that characterized the effects of a TF mutation based on multiple genomic profiles acquired in patient-derived induced cardiomyocytes (iCM). The dataset represents a family where half of the individuals suffer from penetrant septal defects caused by GATA4 mutation (referred to as MUT, Fig. 3A). The original study elucidated the effects of GATA4 mutation on polyA mRNA transcripts based on RNA-seq and cardiac TF binding profiles [16]. Here, we aimed to extend the characterization of GATA4 effects by focusing on transcriptional regulation of cardiac pri-miRNAs using the H3K36me3 profiles collected.

The original study included the ChIP-seq data to complement the analysis of GATA4-regulated gene loci. Therefore, we first ensured the data quality for our purpose to assess cell-type-specific transcript expression based on the available ChIP-seq samples. We quantified the levels of heart development associated genes, collected based on literature, and compared their expression in the iCM cells to endothelial and fibroblast cultures (ENCODE consortium, accession numbers in Table S1) representing the other main cell types in the cardiac tissue environment (Fig. 3B). Despite the mutation, the heart developmental associated genes analyzed, except *TBX3*, were higher transcribed in all of the iCM samples compared to the reference cell types, in agreement with mRNA-seq and physiological characterization in the original study [16]. Second, we analyzed the relationship between the number of reads mapping to pri-miRNA loci (shown in yellow) and the number of pri-miRNA expressed per sample (dotted line) across the iCM datasets (Fig. 3C). Transcript detection was robust despite the variability in library sizes. In total, an average of 855 pri-miRNA transcripts out of 1,848 annotated pri-miRNA transcripts were expressed in the control iCM cells at day 32 of differentiation and the majority of the transcripts could be detected at 10–25 RPKM level, as shown in the histogram in Fig. 3D.

**Fig. 4:**
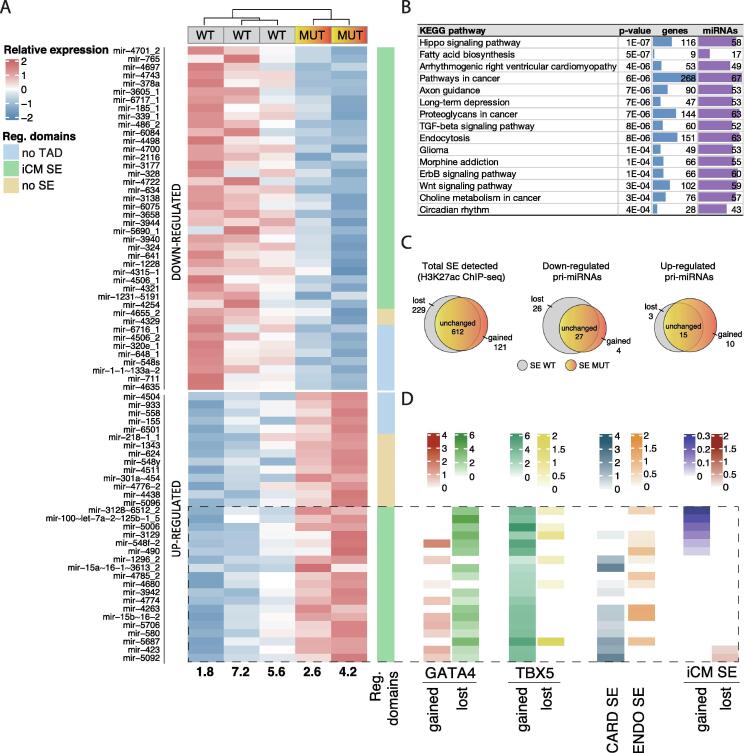
**Differentially expressed pri-miRNAs and functional annotation of putative regulatory regions.** (A) A heatmap of relative expression of DE pri-miRNAs. Values are row-scaled and denoted from high to low expression (in red and blue colors, respectively). Reg. domains sidebar contains pri-miRNAs regulatory domain annotation. (B) Pathway analysis of putative targets of mature forms of DE pri-miRNAs, ordered by the level of significance. The number of genes in each pathway is represented with blue bars and the number of mature miRNAs with purple bars. (C) Summary of H3K27ac peaks detected as SEs that are either unchanged, gained or lost between WT and MUT samples is shown as Venn diagrams: left, total SE changes genome-wide; middle, down-regulated or, right, up-regulated pri-miRNA regulatory domains. (D) GATA4 and TBX5 sidebars contain summarized TF peak numbers by up-regulated pri-miRNA locus that are either gained or lost between WT and MUT samples. Annotation of published SEs within each pri-miRNA regulatory domain is depicted in either purple (cardiac tissue SE) or orange (endothelial SE). iCM sidebar corresponds to annotated SEs within pri-miRNA regulatory domains that were either unchanged, gained, or lost between WT and MUT samples. (For interpretation of the references to color in this figure legend, the reader is referred to the web version of this article.)

To discern how pri-miRNA transcription is affected by the presence of the GATA4 G296S mutation, we performed statistical analysis comparing three samples (iPS-lines 1.8, 5.6 and 7.2) carrying intact GATA4 (WT) and two samples representing the mutation (denoted MUT, iPS-lines 2.6 and 4.2) (Fig. 3A). At day 32 of differentiation, we detected 75 DE pri-miRNA transcripts that passed a pre-filtering step (see Methods), were annotated in miRbase v.22 [26] and obtained a nominal p.value < 0.05. Among these DE pri-miRNAs, 42 were downregulated (from −1.2 to −0.29 log2 fold-change) and 33 were upregulated (from 0.28 to 1.877 log2 fold-change) (Fig. 4A).

In order to discern how changes in pri-miRNA levels may affect cellular functions, we analyzed which signalling pathways could be altered as a consequence of changes in pri-miRNA expression upon GATA4 G296S mutation. We assigned mature miRNA species to DE pri-miRNA based on public cardiac small RNA-seq data and performed pathway analysis based on their experimentally validated or predicted target genes available in TarBase (Fig. 4B) [27], [28].

Hippo signalling was the most significant pathway associated with mature forms derived from DE pri-miRNAs (Fig. S4A, a summary of experimentally confirmed miRNA targets). Disease pathways (Arrhythmogenic right ventricular cardiomyopathy, Long-term depression), and signaling pathways (ErbB, Wnt, Neurotrophin) were enriched in addition to Hippo signaling. The number of putative target genes and miRNAs targeting them also implicated some neuronal and cancer-related pathways (Proteoglycans in cancer, Pathways in cancer, Renal cell carcinoma, Axon guidance, Adherens junction, Endocytosis, and Colorectal cancer) that may potentially relate to calcium signalling and cardiac remodelling [29]. Taken together, the comparison of pri-miRNA transcriptional activities and integrating these with existing bioinformatics tools can guide further functional analysis of the miRNAs in the context of the implicated cellular pathways.

### 2.4 GATA4 G296S mutation impacts pri-miRNA regulation in human iCM

To leverage the collected ChIP-seq data further, we next asked what insights could be gained at the gene regulatory level, to discern how the mutated GATA4 impacted transcriptional regulation at the candidate DE pri-miRNA loci. Towards this end, we focused on contacts betweenenhancers and pri-miRNA promoters, restricted by chromatin structure [30]. We defined so-called regulatory domains for each pri-miRNA locus as the region of chromatin flanked by topologically associating domain (TAD) boundaries (Fig. 4A, regulatory domains sidebar, blue color, see Methods for coordinate retrieval based on Hi-C data from human cardiomyocytes [30], 13 DE pri-miRNAs were not located within the assigned TADs). Additionally, we detected iCM SEs based on the H3K27ac ChIP-seq profiles available directly from the same cellular models: 841 and 733 SEs were detected in WT and MUT samples, respectively.

In total, 51 of DE pri-miRNAs were within regulatory domains that contain SEs (Fig. 4A, green colour, regulatory domains sidebar). We summarized changes by DE pri-miRNA locus: the majority (2/3) of the iCM SEs were among downregulated pri-miRNAs. Moreover, iCM SEs were predominantly lost in downregulated pri-miRNA loci, while new iCM MUT-specific SEs mainly formed within upregulated pri-miRNA regulatory domains (Fig. 4C). The largest TF changes at the up-regulated pri-miRNA loci with iCM SEs corresponded to the loss of GATA4 and gain of TBX5 binding (Fig. 4D, GATA4 and TBX5 sidebars). It has been shown that failure in silencing of the endothelial program is a key step during cardiac development that may affect CHD outcome [16]. Thereafter, we compared the localization of iCM, cardiac and endothelial SEs in pri-miRNA regulatory domains [31]. The majority of these up-regulated pri-miRNA loci with iCM SEs were also associated with endothelial SEs (Fig. 4D).

**Fig. 5:**
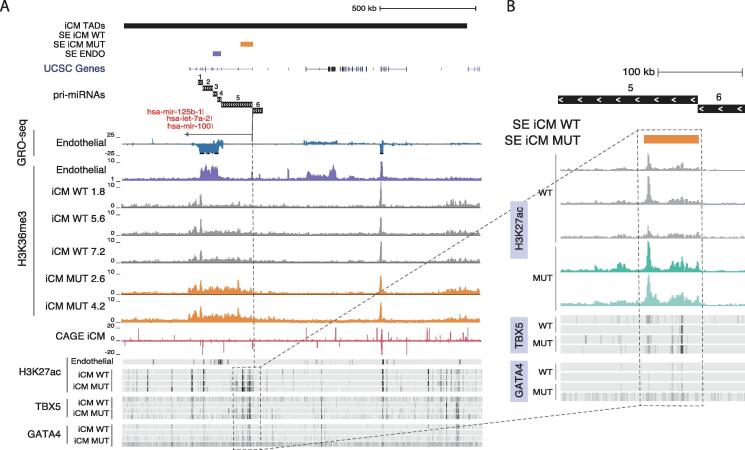
**Effects of pri-miRNA dysregulation exemplified at the hsa-mir-100~let-7a-2~mir-125b-1.** (A) Annotations’ tracks depict firstly iCM TADs from Hi-C data, secondly, SEs detected based on H3K27ac data in endothelial cells (purple bar), in iCM model (WT and MUT, orange bar) and UCSC genes. Signal profiles indicative of active transcription and chromatin state are visualized at the hsa-mir-100~let-7a-2~mir-125b-1 locus (chr11:121,535,430–123,465,441) as follows: GRO-seq (endothelial cells), H3K36me3 ChIP-seq (endothelial cells) followed by ChIP-seq tracks of iCM for H3K36me3, CAGE-seq, and iCM ChIP-seq tracks for H3K27ac, TBX5, and GATA4. (B) The region highlighted on the right (full signal track shown for H3K27ac) corresponds to the TSS5 region and the downstream SE detected based on iCM MUT signal. (For interpretation of the references to color in this figure legend, the reader is referred to the web version of this article.)

Next, we examined in more detail the top upregulated pri-miRNA locus, hsa-mir-100~let-7a-2~mir-125b-1 which also includes multiple targets among the top-ranked pathway (Hippo signaling). This pri-miRNA locus comprises several TSSs of which TSS5 was active in cardiomyocytes and upregulated in MUT samples according to H3K36me3 ChIP-seq signal (1.877 log2 fold-change) (Fig. 4A, 5A-B).

Based on the H3K27ac data, increased transcriptional activity coincides with an iCM MUT gained SE downstream TSS5 (Fig. 5A). Within the gained SE region (Fig. 5B), the H3K27ac signal mainly localizes in two regions: the signal is most elevated downstream the TSS (on the right), extending across >15,000 bp, characteristic of SE regions. Reflecting the changes in TF binding across pri-miRNA loci (Fig. 5A-B), TBX5 ChIP-seq signals increased in this region in MUT samples (Fig. 5A). These results together with those presented in Fig. 4 demonstrate that GATA4 mutation disrupts the transcriptional regulation of several pri-miRNA genes through loss of cardiac SEs and by inducing the formation of a new SE that facilitates binding of TBX5 and potentially other TFs. Quantifying the H3K36me3 signal level together with TF ChIP-seq and enhancer marker profiles could thus be utilized to connect the pri-miRNA TSS activity changes with upstream regulatory programs.

## 3 Discussion

MiRNAs are important players in complex regulatory networks that fine-tune cellular functions. Through the development of new methods to dissect in detail these networks, miRNA studies have been exponentially increasing in a variety of cellular contexts [1]. In spite of equal importance, their primary transcription and TF-mediated regulation is still widely unknown. Here we utilized genome-wide pri-miRNA TSS annotation that was previously generated by integrating data from 27 human cell types [3]. We showed that H3K36me3 ChIP-seq data could serve as a suitable data type for quantifying pri-miRNA expression levels, in addition to nascent transcriptome profiles. This approach allowed us to (1) assess the contribution of cell-type-specific pri-miRNA expression activity in tissue profiles and on pathway activities, (2) enable a wider integration of ENCODE data to study these non-coding genes and (3) provide novel insight into the transcriptional regulation of genome-wide pri-miRNA expression in different disease contexts.

The H3K36me3 histone marker is deposited co-transcriptionally by SETD2 in humans that travels with Pol II during transcription. Therefore, it serves as a marker for actively transcribed gene bodies, including those corresponding to pri-miRNAs [32], [33]. Previously, H3K36me3 histone modification has been widely profiled across cells by ENCODE. Independent studies have further established it as a marker to dissect active gene regions from inactive ones, also during cardiac differentiation [31], [34]. In agreement with these findings, our genome-wide comparison with GRO-seq found the highest correlation with H3K36me3 and H3K79me2 levels among the histone mark collection available in ENCODE. Wang et al. proposed that H3K79me2 histone modification is enriched in genomic regions with high gene expression and that contain a large number of genes with a housekeeping role [5]. When correspondence at the level of transcriptional activity bins was evaluated, we noticed that H3K36me3 profile resulted in a more comparable level of transcription to GRO-seq data, making it better suitable than H3K79me2. In the context of miRNA gene annotation, Liu and colleagues exploited H3K36me3 ChIP-seq data to support the identification of active TSSs of miRNAs based on GRO-seq, by evaluating the level of this marker before and after identified genomic TSSs [35]. Thus, once the coordinates are established, both GRO-seq and H3K36me3 data types enable the comparison of pri-miRNA levels across samples. Currently, there are 547 human GRO-seq samples and 665 human H3K36me3 ChIP-seq samples available in the NCBI GEO data repository (March 2021, Table S3) that allows the quantification of pri-miRNA expression as demonstrated here. Thus, our work considerably widened the use of publicly available data for characterizing pri-miRNA gene transcriptional activity. Additionally, caRNA-seq, mNET-seq, PRO-seq and TT-seq represent other data types that capture nascent RNA [36] and could be utilized similarly to capture pri-miRNA expression. Furthermore, these methods provide slightly different information (reviewed in [36]). Combination with H3K36me3 ChIP-seq could be informative to elucidate additional regulatory steps, such as co-transcriptional processing efficiency in loci encoding multiple miRNAs. Our comparison of pri-miRNA synthesis vs mature miRNA levels supports that this may serve as an additional regulatory step.

The limitations of using H3K36me3 data to quantify gene expression include lack of strand-specificity and increased background signal in comparison with nascent transcriptomes. It would be possible to address the former by modifying the TSS elements used in the pri-miRNA quantification by excluding the overlapping region between pri-miRNAs and genes in opposite strands. For regions where transcripts completely overlap, additional experiments such as CRISPR-mediated deletion of each transcript TSS separately could help discern which transcript is contributing to the overall signal. To deal with the variable background signal in distinguishing lower expressed pri-miRNA transcripts, tools that are applied to ChIP-seq peak identification can be used to discern at which genomic locations, the signal is enriched over the background signal [37], [38]. Furthermore, ensuring the quality of the sequencing data is another consideration that should be addressed prior to quantification of the signal levels. This data quality assessment could be performed following the quality standards proposed by ENCODE.

We show that H3K36me3-based quantification could enable assessing the contribution of different cell types present in a tissue environment (such as the cardiac environment) to the miRNA expression in normal and disease states. Pri-miRNA transcripts are further processed into hairpins called premature miRNAs exported from the nucleus to the cytoplasm and further processed into mature miRNA forms [2]. Pri-miRNAs and mature miRNAs have a similar relation as primary RNAs and mature RNAs, affected by synthesis, processing, and degradation rates. Furthermore, multiple transcript variants and genomic loci can influence the mature form expression level. Therefore, our workflow to quantify the pri-miRNA levels can lead to specific mature miRNA candidates, nevertheless, those need to be experimentally validated in order to correlate the pri-miRNA TSS activity to the mature miRNA output. Among disease-associated pri-miRNAs that were distinctive for cardiac cell types, hsa-mir-193b~365a cluster was found to be highly expressed in fibroblasts. The mature miRNA expression level has been shown to return to physiological levels after introducing Left Ventricular Assist Device into the patients’ hearts [19]. This intervention was shown to modulate non-cardiomyocyte cell phenotypes [39]. In this manner, discerning whether the differential expression originated from fibroblasts or cardiomyocytes through integrative data analysis, followed by further characterization in the appropriate cell models, could have important implications for targeted therapy to restore tissue homeostasis after myocardial infarction and surgery.

In this study, we further examined cancer pri-miRNA signatures from ENCODE data. Focusing on a candidate list of miRNAs associated with cancer gene signatures, we quantified their transcriptional activities across multiple cell lines. Elevated expression of miRNAs encoded from hsa-mir-15a~16–1~3613 locus has been associated with glucocorticoid resistance in acute lymphoblastic leukemia [40], whereas mir-15 deletion has been linked to overexpression of apoptotic gene BCL2 in cancer [41]. Based on our results, it would be important to further discern the contribution of differential TSS usage on the disease onset [41]. Overall, our analysis highlights the high variation between the different cancer cell lines in both the basal transcriptional activity and differential TSS utilization. Through the ENCODE data resource, it would now be possible to investigate how different stimuli, TFs and DNA sequence mutations impact the TSS activity, and in parallel to investigate epigenetic mechanisms that may act to silence such tumor suppressor miRNAs, through quantitative analysis of ChIP- and DNAse-seq signals across the cell lines.

Cellular homeostasis is subjected to complex regulation that is coordinated by shared or cell-type-specific signaling pathways, TFs and miRNAs. We showed here that H3K36me3-based quantification could distinguish cell-type-specific expression and extend the analysis of regulatory networks to include pri-miRNA transcripts. In our analysis of altered pri-miRNA regulation in cardiac disease, we detected 75 candidate DE pri-miRNAs. The mature miRNA forms derived from these loci have been linked to CHDs or heart diseases (reviewed by [12], [13]), thus further supporting the H3K36me3 level as a suitable measure of transcriptional changes. Among those, miR-1–1 was previously found to be down-regulated in cardiac tissue of patients with CHD/septal defects [42]. Our study confirms that this downregulation occurs directly in cardiomyocytes carrying the GATA4 G296S mutation. We acknowledge that this case study is limited in statistical power by the number of samples available. The primary purpose of H3K36me3 ChIP-seq data has been to capture chromatin dynamics, therefore future studies that aim to leverage this marker for statistical analysis should be designed with a higher number of replicates to have improved statistical power.

Focusing on putative targets corresponding to the mature forms of DE pri-miRNAs, our pathway analysis revealed several key cardiac pathways. Notably, several DE miRNAs were associated with Hippo signaling that has been shown to have a central role in organ development and tissue homeostasis by controlling proliferation, cell fate decisions and survival (considering heart development reviewed by [43]). Dysregulation of this pathway is associated with development defects including septal defects and YAP1, the target of the mature form of hsa-mir-100~let-7a-2~mir-125b-1, is down-regulated in the disease condition [44]. Our analysis provides a rationale to perform further mechanistic studies to characterize the interplay between altered TF recruitment and post-transcriptional regulation via miRNAs which can perturb cardiac cell homeostasis by modulating the expression of this pathway.

We have previously shown that pri-miRNA loci display a complex regulatory structure, where distinct TSS interacts with distal enhancers [3]. In a similar fashion that promoters for protein-coding genes, pri-miRNA TSS-to-enhancer contacts are also confined by chromatin architecture. Distal enhancers up- and downstream from promoter regions can be brought into close proximity and influence transcription and stimulate cell-type-specific differential TSS utilization within the same chromatin domain [3], [45]. Here, we explored regulatory interactions associated with differential pri-miRNA expression. Focusing on SEs located within regulatory domains of DE pri-miRNAs, we noted that a majority of DE pri-miRNA loci included SEs that were lost or gained in the MUT samples. Loss of SEs associated with downregulated pri-miRNAs. Furthermore, we observed the formation of new iCM SEs in MUT samples within regulatory domains with upregulated pri-miRNAs. Our analysis revealed that a major part of these gained iCM SEs had an increasing number of TBX5 binding sites. Specifically, among DE pri-miRNA, we annotate a novel SE downstream hsa-mir-100~let-7a-2~mir-125b-1 TSS5 that is gained in patients carrying GATA4 mutation and contains TBX5 gained peaks. These results demonstrated that the GATA4 mutation impacts the transcriptional regulation of pri-miRNAs. These studies could also be extended to include binding profiles for additional cardiac TFs to gain a better understanding of the mechanisms that contribute to the formation of these SE. Overall, the integrative analysis across different genomic data highlighted changes not only at pri-miRNA expression levels but also in fine-tuning of chromatin states between WT and MUT samples that affect pri-miRNA transcriptional regulation.

In conclusion, the approach we present can advance research on miRNA regulation based on existing and well-established genome-wide histone marker data. We assessed the pitfalls that arise from the main differences between GRO-seq and H3K36me3 ChIP-seq data and provided suggestions on how to consider them in the analysis and interpretation. Moreover, we improved the global understanding of factors affecting cardiac development, widened the knowledge of the transcriptional regulation of pri-miRNA expression in cardiomyocytes , and highlighted candidate miRNAs for further investigations. Together with other related studies, this paves the way to reconstruct TF-pri-miRNA networks that are fundamental in regulating cell identity. Methods for understanding cell-type-specific regulatory mechanisms that are dysregulated in disease pathogenesis could benefit drug discovery by assessing each component of the regulatory network, thus providing a rational approach to develop more efficient treatments.

## 4 Material and methods

### 4.1 Pre-processing ChIP- and GRO-seq data

iCM ChIP-seq data was pre-processed from SRA files that were previously downloaded from the NCBI SRA database with GSE accession number: GSE85631 and GSM identifiers listed in Table S1. The quality of the reads was assessed using FastQC tool (http://www.bioinformatics.babraham.ac.uk/projects/fastqc) and reads were filtered using FastX toolkit (http://hannonlab.cshl.edu/fastx_toolkit/) so that minimum 97% of all bases in one read have to have a minimum phred quality score of 10 [46]. The resulting reads were aligned to the human genome hg19 using bowtie-0.12.7 with parameters set to allow two mismatches, accepting maximum 3 locations per read in the genome and reporting the best alignment found. Additional ChIP-seq data used in this study was downloaded from the ENCODE consortium directory (ftp://hgdownload.cse.ucsc.edu/goldenPath/hg19/encodeDCC/, GEO identifiers are listed in Table S1). Next, the aligned .bam files were used to quantify pri-miRNA coordinates. BigWig files were created using HOMER software v.4.9.1 [47] (makeMultiWigHub.pl) to visualize the data in the UCSC genome browser [48].

### 4.2 Annotation and quantification of pri-miRNA transcripts

Reads mapping to pri-miRNA coordinates were quantified as described in [3] with slight modifications. Briefly, since some tissues (including cardiac) were only represented by one cell line model, we extended the pri-miRNA annotation by generating TSS elements from Refseq and UCSC knownGene annotations (2018) [49], [50] (gene transcript coordinates for intragenic and host-gene (HG) coordinates for intergenic pri-miRNAs) and added to the TSS elements representing further intergenic pri-miRNAs coordinates reported in our previous work based on 27 human cell types [3]. Known annotations were used similarly as the *de novo* transcripts generated from GRO-seq signal and TSS elements that overlapped with pre-miRNA (cluster) genomic coordinates were extracted as previously described. This extended the current annotation by 201 pri-miRNA transcripts (Table S4).

The resulting TSS elements were quantified using HOMER software v.4.9.1 (analyzeRepeats.pl with parameters -strand + -noadj-noCondensing-pc 3). Finally, we estimated the contribution of each TSS in a given locus to the overall transcriptional activity. This was done by subtracting the signal level of the upstream TSS element based on the RPKM values, as previously described [3]. To avoid introducing low expressed transcripts that would raise false positives in the DE analysis, transcripts were considered expressed if the H3K36me3 ChIP-seq signal was 5-fold enriched over the input signal (findPeaks.pl command from HOMER software v.4.9.1 with option -F 5) [47].

### 4.3 Benchmarking the use of H3K36me3 histone mark to detect pri-miRNA expression

Data available from endothelial cells was used to compare transcript expression level quantification between GRO- and ChIP-seq data. A total of 12 different datasets including H3K36me3 were included in the analysis (accession numbers in Table S1). Raw counts for each TSS element were normalized to CPM values. Expressed transcripts (CPM > 1 across all samples) were correlated pairwise using Pearson correlation. H3K36me3 and H3K79me2 signal was further analyzed from A549 (lung cancer), GM lines (lymphoblastoid), IMR-90 (fibroblast), and K562 (myeloid cancer) ChIP-seq data generated by ENCODE. To further evaluate the H3K36me3 and H3K79me2 signal in comparison with GRO-seq signal, we divided 9,053 TSS elements into 100 transcriptional activity groups (bins) based on GRO-seq mean expression and ordered these groups from lowest to highest expressed. Next, the mean histone marker signal for the same groups was calculated. Log transformed average TPM counts were plotted to assess the correspondence of GRO- versus ChIP-seq based transcriptional activity level in groups of genes that are similarly expressed (y-axis), following the approach in [4]. Pri-miRNA transcripts detected by GRO- and ChIP-seq were determined after data type-specific filtering: CPM > 0 for GRO-seq and signal 5-fold over the input for ChIP-seq (findPeaks.pl command from HOMER software v.4.9.1 with option -F 5) [47]. The portion of pri-miRNAs affected by expressed genes on the opposite strand was found using intersect command with parameters -S -f 0.50 (software BEDTools v2.27.1) [51].

We noted that H3K36me3 profiles often have a signal increase at the second annotated exon that corresponds to the first splicing site. The relationship has been observed previously [32], [52], [53] and may indicate a link between the histone marker level and co-transcriptional pre-mRNA processing efficiency. When the H3K36me3 signal is averaged across the transcript length, the size of the first intron may influence the obtained signal level. To overcome this, TSS elements where the first exon is excluded could be utilized. As the relevance of this signal elevation to co-transcriptional pre-miRNA processing has not yet been established, we chose to use the signal quantified from the entire annotated coordinate range.

### 4.4 Assessing the relationship between pri-miRNA transcripts and mature miRNA species

Mature miRNA level is affected by transcription (synthesis) level, transcript processing efficiency, and stability. We compared the level of miRNA expression between datasets that capture pri-miRNA expression (GRO-seq and H3K36me3 ChIP-seq data reflecting transcript synthesis) and datasets that capture mature miRNA expression (small RNA-seq from GSE136813 and microRNA microarray from GSE30512, Table S1). Mature miRNA annotations were converted between miRbase versions using the miRBaseConverter v.1.10.1 [54]. To analyze the relationship between the primary transcript and mature miRNA levels, we chose to keep only the predominant −3p and −5p form of each mature miRNA, and for pri-miRNA transcripts that could be processed to yield multiple different mature forms only the highest −3p or −5p expressed mature miRNA form was analyzed for simplicity. Furthermore, only well-annotated miRNAs (hsa- prefix in miRBase) were used. Summed TSS activity for these pri-miRNA loci (log2 RPKM) based on H3K36me3 ChIP-seq data was divided into 10 activity bins (921 pri-miRNA loci) The detection rate was calculated using both mature miRNA microarray and small RNA-sequencing data. Those pri-miRNA loci that had at least one mature miRNA detected were then plotted showing the signal distribution for each data type as violin plots (GRO-seq, H3K36me3 ChIP-seq, small RNA-seq and array).

### 4.5 Statistical analysis of pri-miRNA expression in iCM cells

The publicly available ChIP-seq data was obtained from iPS cardiomyocytes that were differentiated from induced pluripotent stem cells derived from patients suffering from CHD. The patients were previously genotyped to have a heterogenous G296S mutation in the cardiogenic TF GATA4 [16]. TSS elements representing pri-miRNA transcripts were overlapped with peaks found enriched by findPeaks command as described above and those lacking enriched peaks were discarded. Differentially expressed transcripts were calculated with limma R package that fits the data to a linear model and uses the voom transformation for introducing weights to capture the mean–variance relationship in count data [55]. Transcripts with a nominal p.value < 0.05 we included in the downstream analysis. To overcome the limitation of ChIP-seq data lacking strand-specificity, we excluded those pri-miRNAs with >25% overlap with an expressed gene on the opposite strand based on H3K36me3 data (software BEDTools v2.27.1 with parameters -S -f 0.25). After exclusion of sample 3.2, with insufficient library sequencing depth to provide enough sensitivity in the detection of pri-miRNA expression (Fig. S5A-B) [46], the low number of replicates was estimated to lead to a loss in sensitivity to detect DE genes. Therefore, we opted to assign DE genes using the nominal p-value from the genome-wide quantification (statistical model fitting included Refseq/UCSC and pri-miRNA genes, only the latter were reported). To limit the number of false-positive findings, we examined all DE pri-miRNAs based on the signal tracks and excluded 3 DE pri-miRNAs transcripts due to overlapping genes that were actively transcribed from the opposite strand, and six transcripts due to low and variable signal between the replicates.

### 4.6 Regulatory analysis of pri-miRNA loci

We defined regulatory domains as limited by TAD boundaries. Coordinates of TADs determined using Hi-C from cardiomyocytes were downloaded from GEO (GSE106687). Original coordinates were aligned to hg38 and we lifted them over to hg19 using UCSC liftOver tool [56]. Peak calling was performed for GATA4 and TBX5 ChIP-seq data using findPeaks command [47] with parameters -style factor -o auto. We excluded GATA4 and TBX5 binding peaks that were located ±250 bp from a TSS (promoter binding) of DE pri-miRNA before calculating the number of TF peaks within regulatory domains. The co-operation of GATA4 and TBX5 was found by using intersect command with default parameters (software BEDTools v2.27.1) [51]. The number of TF peaks were normalized to regulatory domain size for barplots. Portions of lost, unchanged, and gained TF peaks were determined based on the number of overlapping peaks between WT and MUT samples. Data was visualized using UCSC Genome Browser [48], [57]. SE annotation for mature cells was downloaded from the dbSUPER database for endothelial cell line and cardiomyocytes (Left Ventricle and Right Ventricle) [58]. Schematic illustrations were created with BioRender.com.

### 4.7 SE detection in iCM samples

H3K27ac tag directories were pooled for WT samples (1.8, 5.6 and 7.2) and MUT samples (2.6 and 4.2). SEs were detected in iCM cells by using HOMER software v.4.9.1 [47] (annotatePeaks.pl with parameters -style super -L 0).

### 4.8 Pathway analysis

We obtained mature miRNA −3p and −5p forms from miRBase v.20 [26] and filtered out the −3p or −5p form that was not expressed (RPKM < 0.5) based on the following cardiomyocyte small RNA-seq datasets: GSE108021 (samples GSM2887374, GSM2887375, and GSM2887376), GSE62913 and GSE60292, under physiological conditions. In order to perform pathway analysis, we used mirPath v.3 online tool, which contains experimentally validated and predicted miRNA gene targets, KEGG and GO pathways [59]. We selected the top 15 KEGG pathways ranked according to their significance (p.value < 0.05, Fisher's Exact Test). Experimentally validated gene targets were obtained from TarBase v.8 [60]. For each mature miRNA of the hsa-mir-100~let-7a-2~mir-125b-1 cluster, target genes were plotted into the hippo pathway map (accession hsa04390) using KEGG Mapper tool [61].

## Declaration of Competing Interest

The authors declare that they have no known competing financial interests or personal relationships that could have appeared to influence the work reported in this paper.
