# Supplementary material for: Genome-wide analysis of primary microRNA expression using H3K36me3 ChIP-seq data
Source: Comput Struct Biotechnol J. 2021 Apr 5;19:1944–55. doi: 10.1016/j.csbj.2021.03.035 (PMC8082160; doi:10.1016/j.csbj.2021.03.035)
Supplement: Supplementary data 1 [file mmc1.pdf]

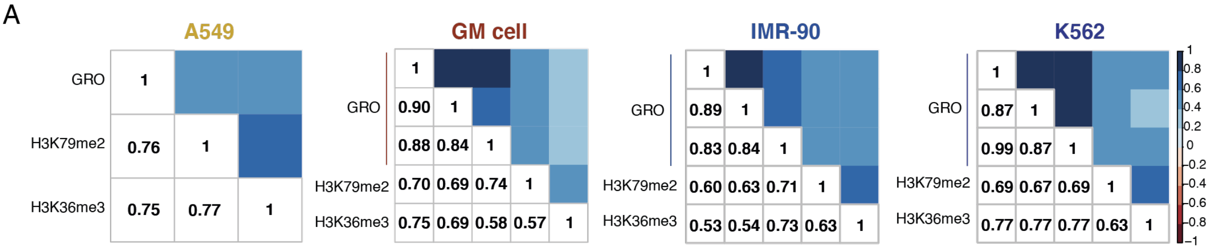


**Figure S1. Correlations of histone modifications and GRO-seq profiles in selected cell lines.** (A) Pearson correlation of primary transcript expression (counts per million, CPM) quantified based on GRO-seq and histone ChIP-seq data from A549, GM lines, IMR-90, and K562 is shown as in Fig. 1A (blue tones correspond to positive correlation, whereas red tones correspond to negative correlation from 1 to -1).

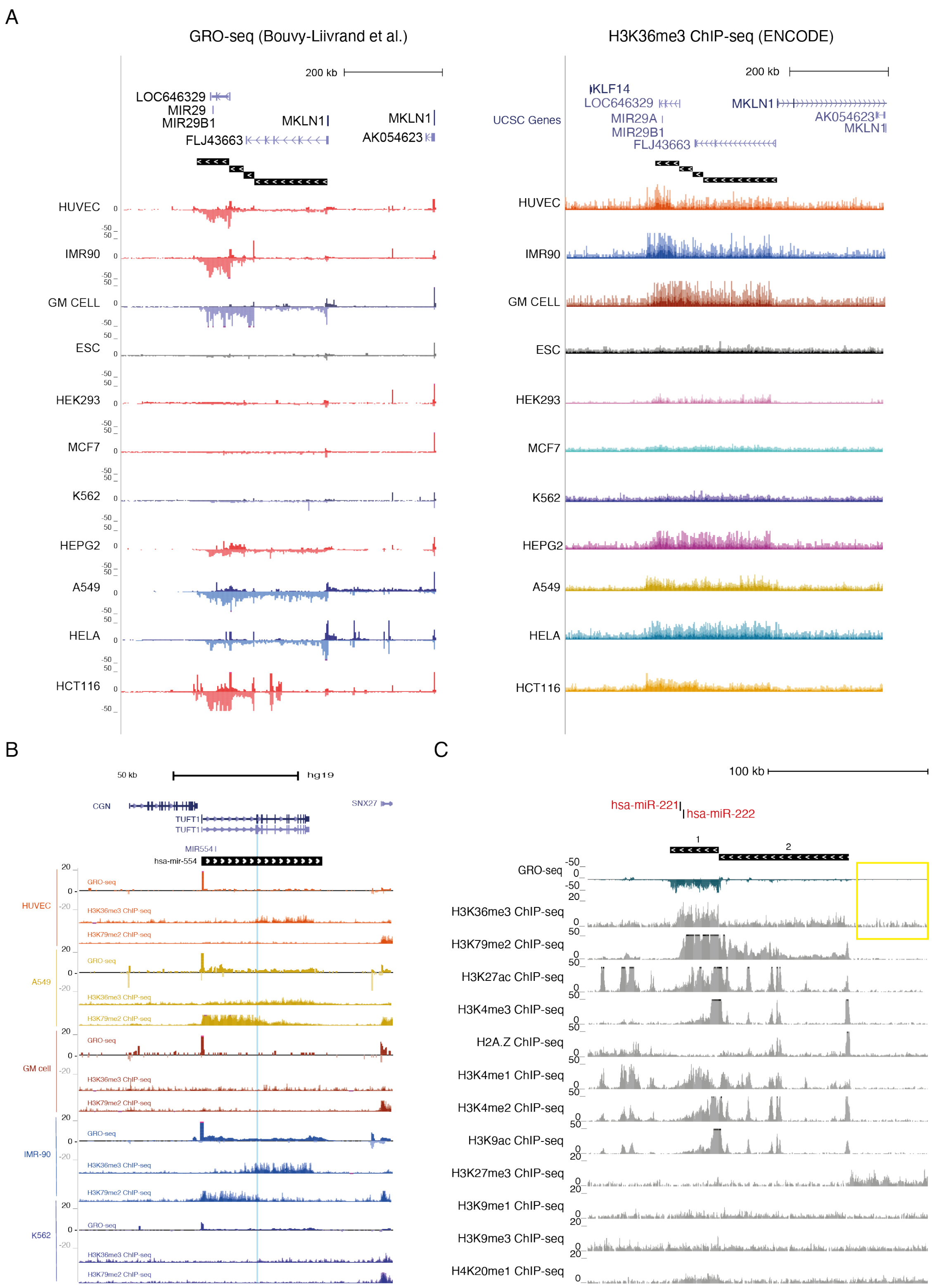


**Figure S2. Signal profiles in additional cell lines.** (A) GRO-seq profiles of cancer cell lines visualized at the hsa-mir-29a~hsa-mir-29b-1 locus (chr7:130 376 783–131 016 782) and H3K36me3 ChIP-seq signal visualized at the hsa-mir-29a~hsa-mir-29b-1 locus of the same cancer cell lines. (B) Signal profiles visualized at the hsa-mir-554 locus (chr1:151463644-151589254) as follows: GENCODE annotation, custom annotation tracks indicating the region used for quantification of output from each pri-miRNA TSS (black box with arrows pointing right as hsa-mir-554 is transcribed from the plus strand). Signal tracks are displayed in groups for each cell line shown (HUVEC, A549, GM cell, IMR-90 and K562). Each group is composed of GRO-seq, H3K36me3 and H3K79me2 ChIP-seq tracks. (C) Signal profiles visualized at the hsa-mir-221~222 locus (chrX:45584514-45719179) as follows: GRO-seq, followed by ChIP-seq tracks for H3K36me3, H3K79me2, H3K27ac, H3K4me3, H2A.Z, H3K4me1, H3K4me2, H3K9ac, H3K27me3, H3K9me1, H3K9me3, and H4K20me1.

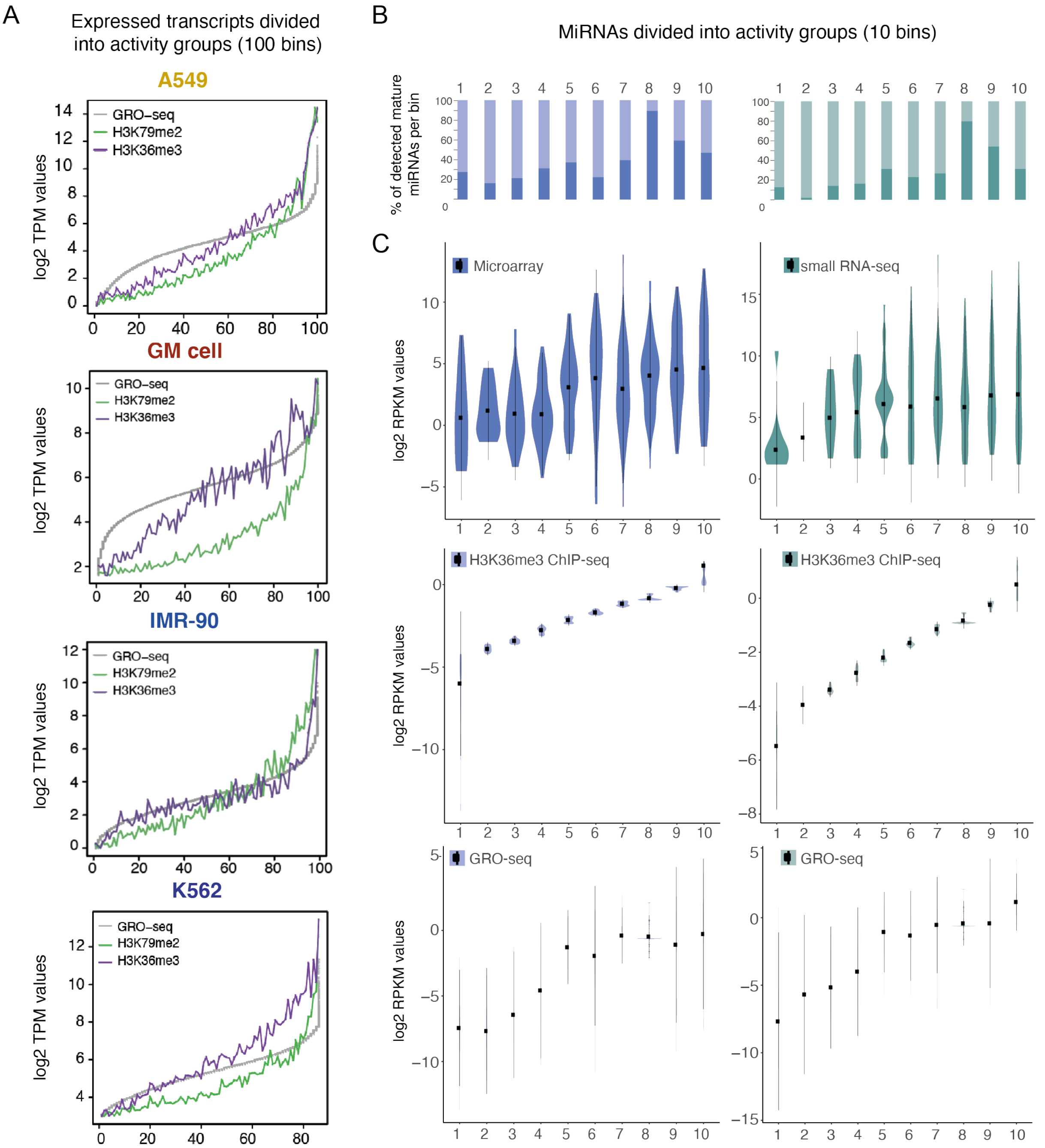


**Figure S3. Histone modifications ChIP-seq profiles in additional cell lines and quantification of mature and primary miRNAs in selected datasets.** (A) Expressed transcripts were separated into 100 bins according to their mean expression levels based on GRO-seq data (log2 transformed transcripts per million (TPM), grey line) as shown in Fig. 1C. Histone marker average tag density (H3K36me3 purple and H3K79me2 green, y-axis) was plotted for each bin (ordered from lower to higher expressed transcripts, x-axis) in A549, GM lines, IMR-90, and K562 cell lines. (B) Summed TSS activities based on H3K36me3 ChIP-seq data corresponding to each pri-miRNA locus were separated into 10 bins. The barplots describes the proportions of mature miRNA detected per bin by either microarray methodology (in dark blue color) or small RNA-seq assay (in dark green color). (C) Violin plots showing the log2 RPKM expression values (y-axis) are depicted for each bin (x-axis from low to higher expressed miRNAs) from each data type. On the left, the data shown corresponds to mature miRNAs that are detected by microarray (shown in panel B in dark blue). On the right mature miRNAs detected by small RNA-seq are shown similarly (shown in panel B in dark green).

A

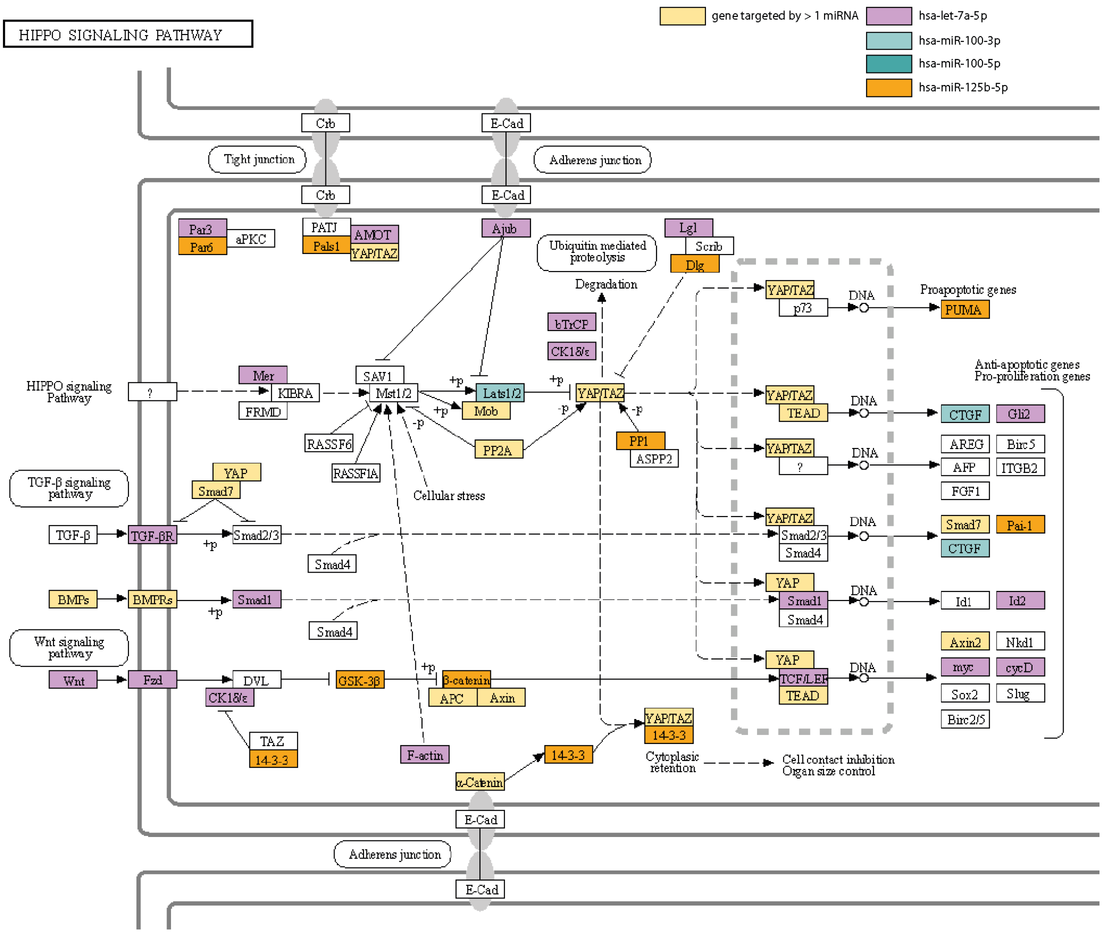


**Figure S4. Hippo signaling pathway.** Genes that are targeted by more than one miRNA, hsa-let-7a, hsa-miR-100-3p, hsa-miR-100-5p, and hsa-miR-125b-5p are colored yellow, violet, light green, green, and orange, respectively.

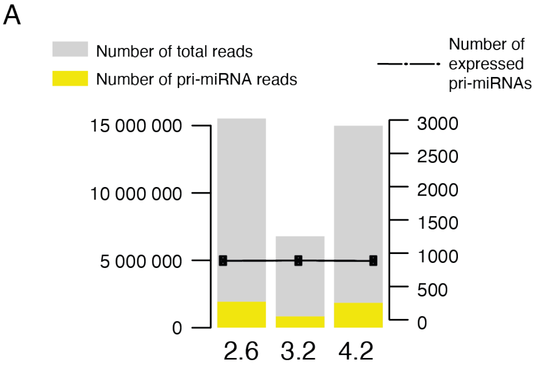


| | Pri-miRNA expressed | Pri-miRNA reads | Total reads - Library size |
|---|---|---|---|
| **2.6** | 885 | 1.927.323 | 15.524.052 |
| **3.2** | 890 | 842.690 | 6.786.820 |
| **4.2** | 882 | 1.845.485 | 14.989.588 |

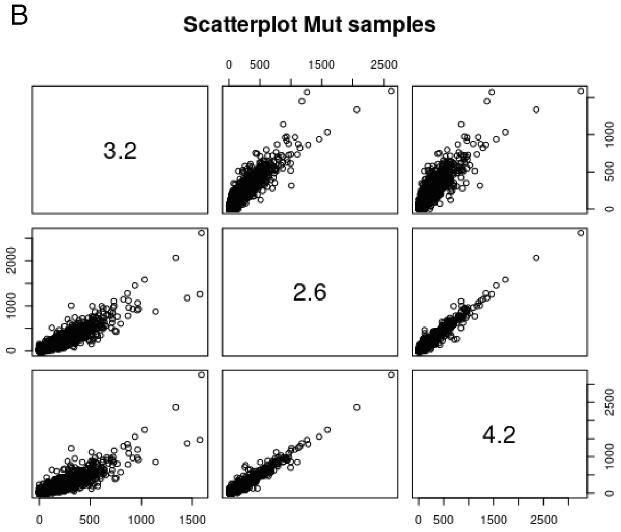


**Figure S5. 3.2 Sample exclusion.** (A) Library sizes (total number of reads, in grey) and the portion of reads that map to pri-miRNAs (in yellow) are shown as overlaid bar plots. The y-axis on the left indicates the number of reads. The dotted line and the axis on the right indicate the number of pri-miRNAs detected enriched over the input signal per sample. (B) Scatterplot comparing the three samples derived from individuals carrying a mutation in GATA4 TF.

## Table S1. Data used in this study.

| GSE id | GSM id | Cell.Type | Assay | Condition | Replicate | Genome Version | Source |
|---|---|---|---|---|---|---|---|
| GSE52642 | GSM1412749 | HUVEC | GRO-seq | none | 2 | hg19 | umbilical cord vein |
| GSE52642 | GSM1273483 | HUVEC | GRO-seq | none | 1 | hg19 | umbilical cord vein |
| GSE92375 | GSM2486423 | HUVEC | GRO-seq | none | 1 | hg19 | umbilical cord vein |
| GSE92375 | GSM2643991 | HUVEC | GRO-seq | none | 1 | hg19 | umbilical cord vein |
| GSE39878 | GSM980644 | GM12004 | GRO-seq | none | 1 | hg19 | B-lymphocyte |
| GSE39878 | GSM980645 | GM12750 | GRO-seq | none | 1 | hg19 | B-lymphocyte |
| GSE60454 | GSM1480326 | GM12878 | GRO-seq | none | 1 | hg19 | B-lymphocyte |
| GSE29611 | GSM1003556 | HUVEC | ChIP-seq | H2az | 1:2 | hg19 | umbilical cord vein |
| GSE29611 | GSM733690 | HUVEC | ChIP-seq | H3K4me1 | 1:3 | hg19 | umbilical cord vein |
| GSE29611 | GSM733683 | HUVEC | ChIP-seq | H3K4me2 | 1:2 | hg19 | umbilical cord vein |
| GSE29611 | GSM733673 | HUVEC | ChIP-seq | H3K4me3 | 1:3 | hg19 | umbilical cord vein |
| GSE29611 | GSM733735 | HUVEC | ChIP-seq | H3K9ac | 1:3 | hg19 | umbilical cord vein |
| GSE29611 | GSM733703 | HUVEC | ChIP-seq | H3K9me1 | 1:3 | hg19 | umbilical cord vein |
| GSE29611 | GSM1003517 | HUVEC | ChIP-seq | H3K9me3 | 1:2 | hg19 | umbilical cord vein |
| GSE29611 | GSM733691 | HUVEC | ChIP-seq | H3K27ac | 1:3 | hg19 | umbilical cord vein |
| GSE29611 | GSM733688 | HUVEC | ChIP-seq | H3K27me3 | 1:2 | hg19 | umbilical cord vein |
| GSE29611 | GSM733757 | HUVEC | ChIP-seq | H3K36me3 | 1:3 | hg19 | umbilical cord vein |
| GSE35583 | GSM945233 | HUVEC | ChIP-seq | H3K36me3 | 1:2 | hg19 | umbilical cord vein |
| GSE29611 | GSM1003555 | HUVEC | ChIP-seq | H3K79me2 | 1:2 | hg19 | umbilical cord vein |
| GSE29611 | GSM733640 | HUVEC | ChIP-seq | H4K20me1 | 1:3 | hg19 | umbilical cord vein |
| GSE29611 | GSM733699 | NHLF | ChIP-seq | H3K36me3 | 1:2 | hg19 | lung fibroblasts |
| GSE19465 | GSM621387 | fetal lung | ChIP-seq | H3K36me3 | 1 | hg19 | primary fetal lung tissue |
| GSE16256 | GSM521890 | IMR90 | ChIP-seq | H3K36me3 | 1 | hg19 | fetal lung fibroblast |
| GSE16256 | GSM521892 | IMR90 | ChIP-seq | H3K36me3 | 2 | hg19 | fetal lung fibroblast |
| GSE16256 | GSM521909 | IMR90 | ChIP-seq | H3K79me2 | 1 | hg19 | fetal lung fibroblast |
| GSE16256 | GSM521911 | IMR90 | ChIP-seq | H3K79me2 | 2 | hg19 | fetal lung fibroblast |
| GSE96288 | GSM2534256 | smooth_muscle_cell | ChIP-seq | H3K36me3 | 1 | hg19 | smooth muscle cell |
| GSE96288 | GSM2534257 | smooth_muscle_cell | ChIP-seq | H3K36me3 | 2 | hg19 | smooth muscle cell |
| GSE35583 | GSM945192 | cardiomyocyte | ChIP-seq | H3K36me3 | 1 | hg19 | cardiomyocte |
| GSE35583 | GSM945324 | cardiomyocyte | ChIP-seq | H3K36me3 | 2 | hg19 | cardiomyocte |
| GSE29611 | GSM1003456 | A549 | ChIP-seq | H3K36me3 | 1:2 | hg19 | lung cancer epithelial cell |
| GSE29611 | GSM1003512 | A549 | ChIP-seq | H3K79me2 | 1:2 | hg19 | lung cancer epithelial cell |
| GSE96251 | GSM2534184 | A673 | ChIP-seq | H3K36me3 | 1 | hg19 | Ewing's sarcoma cell |
| GSE96251 | GSM2534185 | A673 | ChIP-seq | H3K36me3 | 2 | hg19 | Ewing's sarcoma cell |
| GSE96454 | GSM2534596 | ACC112 | ChIP-seq | H3K36me3 | 1 | hg19 | adenoid cystic carcinoma |
| GSE96454 | GSM2534597 | ACC112 | ChIP-seq | H3K36me3 | 2 | hg19 | adenoid cystic carcinoma |
| GSE35583 | GSM945206 | Caco-2 | ChIP-seq | H3K36me3 | 1:2 | hg19 | colorectal adenocarcinoma cell |
| GSE29611 | GSM1003463 | DND-41 | ChIP-seq | H3K36me3 | 1:2 | hg19 | T cell leukemic cell |
| GSE86754 | GSM2308610 | DOHH2 | ChIP-seq | H3K36me3 | 1 | hg19 | non-Hodgkin's B-cell lymphoma |
| GSE35583 | GSM733736 | GM12878 | ChIP-seq | H3K36me3 | 1:2 | hg19 | B-lymphocyte |
| GSE29611 | GSM733736 | GM12878 | ChIP-seq | H3K79me2 | 1:2 | hg19 | B-lymphocyte |
| GSE86754 | GSM2308611 | DOHH2 | ChIP-seq | H3K36me3 | 2 | hg19 | non-Hodgkin's B-cell lymphoma |
| GSE95914 | GSM2527452 | HCT116 | ChIP-seq | H3K36me3 | 1 | hg19 | colon cancer epithelial cell |
| GSE86668 | GSM2308432 | HCT116 | ChIP-seq | H3K36me3 | 1 | hg19 | colon cancer epithelial cell |
| GSE86668 | GSM2308433 | HCT116 | ChIP-seq | H3K36me3 | 2 | hg19 | colon cancer epithelial cell |
| GSE29611 | GSM733711 | HeLa-S3 | ChIP-seq | H3K36me3 | 1:2 | hg19 | cervical cancer epithelial cell |
| GSE35583 | GSM945230 | HeLa-S3 | ChIP-seq | H3K36me3 | 1:2 | hg19 | cervical cancer epithelial cell |
| GSE29611 | GSM733669 | HeLa-S3 | ChIP-seq | H3K36me3 | 1:2 | hg19 | cervical cancer epithelial cell |
| GSE29611 | GSM733685 | HepG2 | ChIP-seq | H3K36me3 | 1:2 | hg19 | hepatocellular carcinoma cell |
| GSE35583 | GSM945211 | HepG2 | ChIP-seq | H3K36me3 | 1:2 | hg19 | hepatocellular carcinoma cell |
| GSE29611 | GSM733651 | K562 | ChIP-seq | H3K36me3 | 1:2 | hg19 | chronic myelogenous leukemic cell |
| GSE35583 | GSM945302 | K562 | ChIP-seq | H3K36me3 | 1:2 | hg19 | chronic myelogenous leukemic cell |
| GSE29611 | GSM733714 | K562 | ChIP-seq | H3K36me3 | 1:2 | hg19 | chronic myelogenous leukemic cell |
| GSE29611 | GSM733653 | K562 | ChIP-seq | H3K79me2 | 1:2 | hg19 | chronic myelogenous leukemic cell |
| GSE86763 | GSM2308628 | Karpas-422 | ChIP-seq | H3K36me3 | 1 | hg19 | non-Hodgkin's B-cell lymphoma |
| GSE86763 | GSM2308629 | Karpas-422 | ChIP-seq | H3K36me3 | 2 | hg19 | non-Hodgkin's B-cell lymphoma |
| GSE96444 | GSM2534580 | KOPT-K1 | ChIP-seq | H3K36me3 | 1 | hg19 | acute lymphoblastic leukemic T-cell |
| GSE96444 | GSM2534581 | KOPT-K1 | ChIP-seq | H3K36me3 | 2 | hg19 | acute lymphoblastic leukemic T-cell |
| GSE96410 | GSM2534508 | Loucy | ChIP-seq | H3K36me3 | 1 | hg19 | acute lymphoblastic leukemic T-cell |
| GSE96410 | GSM2534509 | Loucy | ChIP-seq | H3K36me3 | 2 | hg19 | acute lymphoblastic leukemic T-cell |
| GSE31755 | GSM970217 | MCF-7 | ChIP-seq | H3K36me3 | 1:2 | hg19 | breast cancer epithelial cell |
| ENCSR610IYQ | ENCBS789UPK | MCF-7 | ChIP-seq | H3K36me3 | 1 | hg19 | breast cancer epithelial cell |
| ENCSR610IYQ | ENCBS967MVZ | MCF-7 | ChIP-seq | H3K36me3 | 2 | hg19 | breast cancer epithelial cell |
| GSE95931 | GSM2527488 | MM.1S | ChIP-seq | H3K36me3 | 1 | hg19 | B-cell lymphoma |
| GSE95931 | GSM2527489 | MM.1S | ChIP-seq | H3K36me3 | 2 | hg19 | B-cell lymphoma |
| GSE96236 | GSM2534154 | NCI-H929 | ChIP-seq | H3K36me3 | 1 | hg19 | B-cell lymphoma |
| GSE96236 | GSM2534155 | NCI-H929 | ChIP-seq | H3K36me3 | 2 | hg19 | B-cell lymphoma |
| GSE86709 | GSM2308516 | OCI-LY1 | ChIP-seq | H3K36me3 | 1 | hg19 | non-Hodgkin's B-cell lymphoma |
| GSE86709 | GSM2308517 | OCI-LY1 | ChIP-seq | H3K36me3 | 2 | hg19 | non-Hodgkin's B-cell lymphoma |
| GSE86730 | GSM2308562 | OCI-LY3 | ChIP-seq | H3K36me3 | 1 | hg19 | B-cell lymphoma |

| | | | | | | | |
|---|---|---|---|---|---|---|---|
| GSE86730 | GSM2308563 | OCI-LY3 | ChIP-seq | H3K36me3 | 2 | hg19 | B-cell lymphoma |
| GSE86692 | GSM2308483 | OCI-LY7 | ChIP-seq | H3K36me3 | 1 | hg19 | B-cell lymphoma |
| GSE86692 | GSM2308484 | OCI-LY7 | ChIP-seq | H3K36me3 | 2 | hg19 | B-cell lymphoma |
| GSE86664 | GSM2308424 | Panc1 | ChIP-seq | H3K36me3 | 1 | hg19 | pancreatic carcinoma cell |
| GSE86664 | GSM2308425 | Panc1 | ChIP-seq | H3K36me3 | 2 | hg19 | pancreatic carcinoma cell |
| GSE96418 | GSM2534524 | PC-3 | ChIP-seq | H3K36me3 | 1 | hg19 | prostatic adenocarcinoma cell |
| GSE96418 | GSM2534525 | PC-3 | ChIP-seq | H3K36me3 | 2 | hg19 | prostatic adenocarcinoma cell |
| GSE96155 | GSM2533992 | PC-9 | ChIP-seq | H3K36me3 | 1 | hg19 | non-small lung cancer cell |
| GSE96155 | GSM2533993 | PC-9 | ChIP-seq | H3K36me3 | 2 | hg19 | non-small lung cancer cell |
| GSE101191 | GSM2700198 | SK-N-MC | ChIP-seq | H3K36me3 | 1 | hg19 | neuroblastoma cell |
| GSE101191 | GSM2700199 | SK-N-MC | ChIP-seq | H3K36me3 | 2 | hg19 | neuroblastoma cell |
| GSE35583 | GSM945209 | SK-N-SH | ChIP-seq | H3K36me3 | 1:2 | hg19 | neuroblastoma cell |
| GSE96503 | GSM2534703 | SK-N-SH | ChIP-seq | H3K36me3 | 1 | hg19 | neuroblastoma cell |
| GSE96503 | GSM2534704 | SK-N-SH | ChIP-seq | H3K36me3 | 2 | hg19 | neuroblastoma cell |
| GSE96503 | GSM2534705 | SK-N-SH | ChIP-seq | H3K36me3 | 3 | hg19 | neuroblastoma cell |
| GSE86673 | GSM2308442 | SK-N-SH | ChIP-seq | H3K36me3 | 1 | hg19 | neuroblastoma cell |
| GSE86673 | GSM2308443 | SK-N-SH | ChIP-seq | H3K36me3 | 2 | hg19 | neuroblastoma cell |
| GSE85631 | GSM2279959 | iCM | ChIP-seq | H3K36me3 | 1.8 | hg19 | iPS-derived cardiomyocytes |
| GSE85631 | GSM2280015 | iCM | ChIP-seq | H3K36me3 | 2.6 | hg19 | iPS-derived cardiomyocytes |
| GSE85631 | GSM2280023 | iCM | ChIP-seq | H3K36me3 | 4.2 | hg19 | iPS-derived cardiomyocytes |
| GSE85631 | GSM2279967 | iCM | ChIP-seq | H3K36me3 | 5.6 | hg19 | iPS-derived cardiomyocytes |
| GSE85631 | GSM2280000 | iCM | ChIP-seq | H3K36me3 | 7.2 | hg19 | iPS-derived cardiomyocytes |
| GSE85631 | GSM2280030 | iCM | ChIP-seq | H3K36me3 | 3.2 | hg19 | iPS-derived cardiomyocytes |
| GSE85631 | GSM2279955 | iCM | ChIP-seq | Gata4 | 1.8 | hg19 | iPS-derived cardiomyocytes |
| GSE85631 | GSM2280012 | iCM | ChIP-seq | Gata4 | 2.6 | hg19 | iPS-derived cardiomyocytes |
| GSE85631 | GSM2280020 | iCM | ChIP-seq | Gata4 | 4.2 | hg19 | iPS-derived cardiomyocytes |
| GSE85631 | GSM2279964 | iCM | ChIP-seq | Gata4 | 5.6 | hg19 | iPS-derived cardiomyocytes |
| GSE85631 | GSM2279957 | iCM | ChIP-seq | H3K27ac | 1.8 | hg19 | iPS-derived cardiomyocytes |
| GSE85631 | GSM2280013 | iCM | ChIP-seq | H3K27ac | 2.6 | hg19 | iPS-derived cardiomyocytes |
| GSE85631 | GSM2280021 | iCM | ChIP-seq | H3K27ac | 4.2 | hg19 | iPS-derived cardiomyocytes |
| GSE85631 | GSM2279965 | iCM | ChIP-seq | H3K27ac | 5.6 | hg19 | iPS-derived cardiomyocytes |
| GSE85631 | GSM2279998 | iCM | ChIP-seq | H3K27ac | 7.2 | hg19 | iPS-derived cardiomyocytes |
| GSE85631 | GSM2279961 | iCM | ChIP-seq | input | 1.8 | hg19 | iPS-derived cardiomyocytes |
| GSE85631 | GSM2280017 | iCM | ChIP-seq | input | 2.6 | hg19 | iPS-derived cardiomyocytes |
| GSE85631 | GSM2280025 | iCM | ChIP-seq | input | 4.2 | hg19 | iPS-derived cardiomyocytes |
| GSE85631 | GSM2279969 | iCM | ChIP-seq | input | 5.6 | hg19 | iPS-derived cardiomyocytes |
| GSE85631 | GSM2280001 | iCM | ChIP-seq | input | 7.2 | hg19 | iPS-derived cardiomyocytes |
| GSE85631 | GSM2279963 | iCM | ChIP-seq | Tbx5 | 1.8 | hg19 | iPS-derived cardiomyocytes |
| GSE85631 | GSM2280019 | iCM | ChIP-seq | Tbx5 | 2.6 | hg19 | iPS-derived cardiomyocytes |
| GSE85631 | GSM2280027 | iCM | ChIP-seq | Tbx5 | 4.2 | hg19 | iPS-derived cardiomyocytes |
| GSE85631 | GSM2279971 | iCM | ChIP-seq | Tbx5 | 5.6 | hg19 | iPS-derived cardiomyocytes |
| GSE106687 | GSM2845454 | CM | Hi-C | none | 1 | hg19 | cardiomyocyte |
| GSE106687 | GSM2845455 | CM | Hi-C | none | 2 | hg19 | cardiomyocyte |
| GSE136813 | GSM4058485 | HUVEC | small RNA-seq | none | 2 | hg19 | umbilical cord vein |
| GSE136813 | GSM4058486 | HUVEC | small RNA-seq | none | 3 | hg19 | umbilical cord vein |
| GSE30512 | GSM756817 | HUVEC | microarray | none | 1 | hg19 | umbilical cord vein |

## Table S2. Pri-miRNAs visualized in Fig 2A.

| Heatmap order | Cluster Cell | pri-miRNA |
|---|---|---|
| 1 | cardiomyocyte | hsa-mir-490_1 |
| 2 | fibroblast | hsa-mir-424_hsa-mir-450a-1_hsa-mir-450a-2_hsa-mir-450b_hsa-mir-503_hsa-mir-542_1 |
| 3 | fibroblast | hsa-mir-100_hsa-let-7a-2_hsa-mir-125b-1_2 |
| 4 | endothelial.cell | hsa-mir-1254-1 |
| 5 | fibroblast | hsa-mir-210_1 |
| 6 | fibroblast | hsa-mir-153-1_2 |
| 7 | endothelial.cell | hsa-mir-21_2 |
| 8 | fibroblast | hsa-mir-130a_1 |
| 9 | fibroblast | hsa-mir-106b_hsa-mir-25_hsa-mir-93_1 |
| 10 | fibroblast | hsa-mir-23b_hsa-mir-24-1_hsa-mir-27b_hsa-mir-6081_2 |
| 11 | fibroblast | hsa-mir-199a-1_1 |
| 12 | fibroblast | hsa-mir-1185-1_hsa-mir-1185-2_hsa-mir-1193_hsa-mir-1197_hsa-mir-127_hsa-mir-134_hsa-mir-136_hsa-mir-154_hsa-mir-299_hsa-mir-300_hsa-mir-323a_hsa-mir-323b_hsa-mir-329-1_hsa-mir-329-2_hsa-mir-337_hsa-mir-369_hsa-mir-370_hsa-mir-376a-1_hsa-mir-376a-2_hsa-mir-376b_hsa-mir-376c_hsa-mir-377_hsa-mir-379_hsa-mir-380_hsa-mir-381_hsa-mir-382_hsa-mir-409_hsa-mir-410_hsa-mir-411_hsa-mir-412_hsa-mir-431_hsa-mir-432_hsa-mir-433_hsa-mir-485_hsa-mir-487a_hsa-mir-487b_hsa-mir-493_hsa-mir-494_hsa-mir-495_hsa-mir-496_hsa-mir-539_hsa-mir-541_hsa-mir-543_hsa-mir-544a_hsa-mir-654_hsa-mir-655_hsa-mir-656_hsa-mir-665_hsa-mir-668_hsa-mir-758_hsa-mir-770_hsa-mir-889_1 |
| 13 | fibroblast | hsa-mir-330_1 |
| 14 | fibroblast | hsa-mir-2467_hsa-mir-4440_hsa-mir-4441_3 |
| 15 | fibroblast | hsa-mir-339_1 |
| 16 | fibroblast | hsa-mir-1301_1 |
| 17 | endothelial.cell | hsa-mir-144_hsa-mir-451a_hsa-mir-4732_1 |
| 18 | endothelial.cell | hsa-mir-4440_hsa-mir-4441_3 |
| 19 | endothelial.cell | hsa-mir-342_1 |
| 20 | fibroblast | hsa-mir-324_1 |
| 21 | endothelial.cell | hsa-mir-328_1 |
| 22 | endothelial.cell | hsa-let-7e_hsa-mir-125a_hsa-mir-99b_1 |
| 23 | endothelial.cell | hsa-mir-4800_1 |
| 24 | endothelial.cell | hsa-mir-148b_1 |
| 25 | endothelial.cell | hsa-mir-3614_1 |
| 26 | fibroblast | hsa-mir-548ao_1 |
| 27 | fibroblast | hsa-mir-193b_hsa-mir-365a_1 |
| 28 | fibroblast | hsa-mir-3679_1 |
| 29 | fibroblast | hsa-mir-193a_hsa-mir-365b_hsa-mir-4725_1 |
| 30 | fibroblast | hsa-mir-100_hsa-let-7a-2_hsa-mir-125b-1_4 |
| 31 | fibroblast | hsa-mir-23a_hsa-mir-24-2_hsa-mir-27a_1 |
| 32 | fibroblast | hsa-mir-103a-2_1 |
| 33 | fibroblast | hsa-mir-221_hsa-mir-222_1_1 |
| 34 | fibroblast | hsa-mir-100_hsa-let-7a-2_hsa-mir-125b-1_3 |
| 35 | fibroblast | hsa-mir-3938_3 |
| 36 | fibroblast | hsa-mir-2355_3 |
| 37 | smooth.muscle.cell | hsa-mir-221_hsa-mir-222_2 |
| 38 | smooth.muscle.cell | hsa-mir-5002_hsa-mir-6083_5 |
| 39 | smooth.muscle.cell | hsa-mir-101-2 |
| 40 | smooth.muscle.cell | hsa-mir-2278_hsa-mir-23b_hsa-mir-24-1_hsa-mir-27b_hsa-mir-6081_3 |
| 41 | smooth.muscle.cell | hsa-mir-342_2 |
| 42 | smooth.muscle.cell | hsa-mir-5579_hsa-mir-708_1 |
| 43 | smooth.muscle.cell | hsa-mir-887_1 |
| 44 | smooth.muscle.cell | hsa-mir-625_1 |
| 45 | cardiomyocyte | hsa-mir-181a-2_hsa-mir-181b-2_1 |
| 46 | cardiomyocyte | hsa-mir-101-1_hsa-mir-3671_1 |
| 47 | smooth.muscle.cell | hsa-mir-153-2_1 |
| 48 | smooth.muscle.cell | hsa-mir-378a_1 |
| 49 | cardiomyocyte | hsa-mir-4775_1 |
| 50 | smooth.muscle.cell | hsa-let-7a-2_hsa-mir-100_hsa-mir-125b-1_2 |
| 51 | fibroblast | hsa-mir-92b_1 |
| 52 | fibroblast | hsa-mir-195_hsa-mir-497_1 |
| 53 | endothelial.cell | hsa-let-7c_hsa-mir-125b-2_hsa-mir-99a_1 |
| 54 | smooth.muscle.cell | hsa-mir-138-1_1 |
| 55 | smooth.muscle.cell | hsa-mir-199a-2_hsa-mir-214_1 |
| 56 | smooth.muscle.cell | hsa-mir-423_1 |
| 57 | smooth.muscle.cell | hsa-mir-301a_hsa-mir-454_1 |
| 58 | endothelial.cell | hsa-mir-2116_1 |
| 59 | endothelial.cell | hsa-mir-29b-2_hsa-mir-29c_2 |
| 60 | endothelial.cell | hsa-mir-151a_1 |
| 61 | endothelial.cell | hsa-mir-339_4 |
| 62 | endothelial.cell | hsa-mir-141_hsa-mir-200c_1 |
| 63 | endothelial.cell | hsa-mir-1271_1 |
| 64 | endothelial.cell | hsa-mir-4786_1 |
| 65 | endothelial.cell | hsa-mir-5010_hsa-mir-548at_1 |
| 66 | endothelial.cell | hsa-mir-29b-2_hsa-mir-29c_1 |
| 67 | endothelial.cell | hsa-mir-769_1 |
| 68 | endothelial.cell | hsa-mir-339_3 |
| 69 | endothelial.cell | hsa-mir-195_hsa-mir-497_3 |
| 70 | endothelial.cell | hsa-let-7c_hsa-mir-125b-2_hsa-mir-99a_2 |
| 71 | endothelial.cell | hsa-mir-140_1 |

| | | |
|---|---|---|
| 72 | endothelial.cell | hsa-mir-302a_hsa-mir-302b_hsa-mir-302c_hsa-mir-302d_hsa-mir-367_1 |
| 73 | endothelial.cell | hsa-mir-378e_1 |
| 74 | endothelial.cell | hsa-mir-338_hsa-mir-657_1 |
| 75 | smooth.muscle.cell | hsa-mir-181a-1_hsa-mir-181b-1 |
| 76 | smooth.muscle.cell | hsa-mir-21_3 |
| 77 | smooth.muscle.cell | hsa-mir-15a_hsa-mir-16-1_hsa-mir-3613_3 |
| 78 | cardiomyocyte | hsa-mir-1-1_hsa-mir-133a-2_1 |
| 79 | cardiomyocyte | hsa-mir-374a_hsa-mir-374b_hsa-mir-421_hsa-mir-545_1 |
| 80 | cardiomyocyte | hsa-mir-1277_1 |
| 81 | cardiomyocyte | hsa-mir-361_1 |
| 82 | cardiomyocyte | hsa-mir-3938_1 |
| 83 | cardiomyocyte | hsa-let-7c_hsa-mir-125b-2_hsa-mir-99a_3 |
| 84 | cardiomyocyte | hsa-mir-3938_4 |
| 85 | cardiomyocyte | hsa-mir-218-2_hsa-mir-585_1 |
| 86 | cardiomyocyte | hsa-mir-744_1 |
| 87 | cardiomyocyte | hsa-mir-103a-1_1 |
| 88 | cardiomyocyte | hsa-mir-4797_1 |
| 89 | cardiomyocyte | hsa-mir-1285-1_1 |
| 90 | cardiomyocyte | hsa-mir-15b_hsa-mir-16-2_1 |
| 91 | cardiomyocyte | hsa-mir-17_hsa-mir-18a_hsa-mir-19a_hsa-mir-19b-1_hsa-mir-20a_hsa-mir-92a-1_1 |
| 92 | cardiomyocyte | hsa-mir-548l_1 |
| 93 | cardiomyocyte | hsa-mir-1-2_hsa-mir-133a-1_1 |
| 94 | fibroblast | hsa-mir-125b-1_1 |
| 95 | cardiomyocyte | hsa-mir-218-1_1 |
| 96 | cardiomyocyte | hsa-mir-320b-2_1 |
| 97 | cardiomyocyte | hsa-mir-155_1 |
| 98 | cardiomyocyte | hsa-mir-15a_hsa-mir-16-1_hsa-mir-3613_2 |
| 99 | cardiomyocyte | hsa-mir-548d-1_1 |
| 100 | cardiomyocyte | hsa-mir-32_1 |

## Table S3. GRO-seq and H3K36me3 datasets available in GEO (March 2021).

| Assay | GSEid | GSMid | Cell / Tissue type | Title |
|---|---|---|---|---|
| GRO-seq | GSE41009 | GSM1006728 | H1-hESC | GroSeq_hESC_1-3 |
| GRO-seq | GSE41009 | GSM1006729 | H1-hESC | GroSeq_0hr_1-2 |
| GRO-seq | GSE41009 | GSM1006730 | H1-hESC | GroSeq_1hr_1-2 |
| GRO-seq | GSE41009 | GSM1006731 | H1-hESC | GroSeq_48hr_1-2 |
| GRO-seq | GSE41323 | GSM1014631 | AC16 | GRO-seq_Vehicle_AC16_1 |
| GRO-seq | GSE41323 | GSM1014632 | AC16 | GRO-seq_Vehicle_AC16_2 |
| GRO-seq | GSE41323 | GSM1014633 | AC16 | GRO-seq_TNFa_10m_AC16_1 |
| GRO-seq | GSE41323 | GSM1014634 | AC16 | GRO-seq_TNFa_10m_AC16_2 |
| GRO-seq | GSE41323 | GSM1014635 | AC16 | GRO-seq_TNFa_30m_AC16_1 |
| GRO-seq | GSE41323 | GSM1014636 | AC16 | GRO-seq_TNFa_30m_AC16_2 |
| GRO-seq | GSE41324 | GSM1014637 | MCF7 | GRO-seq_Vehicle_MCF7_1 |
| GRO-seq | GSE41324 | GSM1014638 | MCF7 | GRO-seq_Vehicle_MCF7_2 |
| GRO-seq | GSE41324 | GSM1014639 | MCF7 | GRO-seq_Vehicle_MCF7_3 |
| GRO-seq | GSE41324 | GSM1014640 | MCF7 | GRO-seq_E2_10m_MCF7_1 |
| GRO-seq | GSE41324 | GSM1014641 | MCF7 | GRO-seq_E2_10m_MCF7_2 |
| GRO-seq | GSE41324 | GSM1014642 | MCF7 | GRO-seq_E2_10m_MCF7_3 |
| GRO-seq | GSE41324 | GSM1014643 | MCF7 | GRO-seq_E2_25m_MCF7_1 |
| GRO-seq | GSE41324 | GSM1014644 | MCF7 | GRO-seq_E2_25m_MCF7_2 |
| GRO-seq | GSE41324 | GSM1014645 | MCF7 | GRO-seq_E2_40m_MCF7_1 |
| GRO-seq | GSE41324 | GSM1014646 | MCF7 | GRO-seq_E2_40m_MCF7_2 |
| GRO-seq | GSE41324 | GSM1014647 | MCF7 | GRO-seq_E2_40m_MCF7_3 |
| GRO-seq | GSE43070 | GSM1055806 | IMR90 | Gro-Seq IMR90 |
| GRO-seq | GSE43070 | GSM1055807 | IMR90 | Gro-Seq IMR90+TNF-Œ± |
| GRO-seq | GSE43835 | GSM1067410 | MCF7 | GRO-seq_Vehicle (Re-Sequenced GSM678535 to greater depth) |
| GRO-seq | GSE43835 | GSM1067411 | MCF7 | GRO-seq_Vehicle (Re-Sequenced GSM678536 to greater depth) |
| GRO-seq | GSE43835 | GSM1067412 | MCF7 | GRO-seq_E2_10m (Re-Sequenced GSM678537 to greater depth) |
| GRO-seq | GSE43835 | GSM1067413 | MCF7 | GRO-seq_E2_10m (Re-Sequenced GSM678538 to greater depth) |
| GRO-seq | GSE43835 | GSM1067414 | MCF7 | GRO-seq_E2_40m (Re-Sequenced GSM678539 to greater depth) |
| GRO-seq | GSE43835 | GSM1067415 | MCF7 | GRO-seq_E2_40m (Re-Sequenced GSM678540 to greater depth) |
| GRO-seq | GSE38140 | GSM1124062 | HCT116 | 1hr DMSO treated HCT116 |
| GRO-seq | GSE47805 | GSM1159895 | LNCaP | siCTL_minusDHT_hs26l2_1 |
| GRO-seq | GSE47805 | GSM1159896 | LNCaP | siCTL_plusDHT_hs26l2_2 |
| GRO-seq | GSE47805 | GSM1159897 | LNCaP | siPYGO2_minusDHT_hs26l2_3 |
| GRO-seq | GSE47805 | GSM1159898 | LNCaP | siPYGO2_plusDHT_hs26l2_4 |
| GRO-seq | GSE47806 | GSM1159899 | LNCaP | minusDHT_ASO_CTL_hs15l7_1 |
| GRO-seq | GSE47806 | GSM1159900 | LNCaP | minusDHT_ASO_PCGEM1_hs15l4_3 |
| GRO-seq | GSE47806 | GSM1159901 | LNCaP | minusDHT_ASO_PRNCR1_hs15l7_3 |
| GRO-seq | GSE47806 | GSM1159902 | LNCaP | plusDHT_ASO_CTL_hs15l7_2 |
| GRO-seq | GSE47806 | GSM1159903 | LNCaP | plusDHT_ASO_PCGEM1_hs15l4_4 |
| GRO-seq | GSE47806 | GSM1159904 | LNCaP | plusDHT_ASO_PRNCR1_hs15l7_4 |
| GRO-seq | GSE51224 | GSM1240738 | AC16 | GRO-seq_Vehicle_1 |
| GRO-seq | GSE51224 | GSM1240739 | AC16 | GRO-seq_Vehicle_2 |
| GRO-seq | GSE51224 | GSM1240740 | AC16 | GRO-seq_TNFŒ±_10m_1 |
| GRO-seq | GSE51224 | GSM1240741 | AC16 | GRO-seq_TNFŒ±_10m_2 |
| GRO-seq | GSE51224 | GSM1240742 | AC16 | GRO-seq_TNFŒ±_30m_1 |
| GRO-seq | GSE51224 | GSM1240743 | AC16 | GRO-seq_TNFŒ±_30m_2 |
| GRO-seq | GSE51224 | GSM1240744 | AC16 | GRO-seq_TNFŒ±_120m_1 |
| GRO-seq | GSE51224 | GSM1240745 | AC16 | GRO-seq_TNFŒ±_120m_2 |
| GRO-seq | GSE51224 | GSM1240746 | AC16 | GRO-seq_control_1 |
| GRO-seq | GSE51224 | GSM1240747 | AC16 | GRO-seq_control_2 |
| GRO-seq | GSE51224 | GSM1240748 | AC16 | GRO-seq_Œ±-amanitin_1 |
| GRO-seq | GSE51224 | GSM1240749 | AC16 | GRO-seq_Œ±-amantin_2 |
| GRO-seq | GSE51633 | GSM1249869 | HEK293T | Gro-seq-siCTL-re1-HEK293T |
| GRO-seq | GSE51633 | GSM1249870 | HEK293T | Gro-seq-siJMJD6-1-re1-HEK293T |
| GRO-seq | GSE51633 | GSM1249871 | HEK293T | Gro-seq-siJMJD6-2-re1-HEK293T |
| GRO-seq | GSE51633 | GSM1249872 | HEK293T | Gro-seq-siBrd4-1-re1-HEK293T |
| GRO-seq | GSE51633 | GSM1249873 | HEK293T | Gro-seq-siBrd4-2-re1-HEK293T |
| GRO-seq | GSE51633 | GSM1249874 | HEK293T | Gro-seq-siCTL-re2-HEK293T |
| GRO-seq | GSE51633 | GSM1249875 | HEK293T | Gro-seq-siJMJD6-1-re2-HEK293T |
| GRO-seq | GSE51633 | GSM1249876 | HEK293T | Gro-seq-siJMJD6-2-re2-HEK293T |
| GRO-seq | GSE51633 | GSM1249877 | HEK293T | Gro-seq-siBrd4-1-re2-HEK293T |
| GRO-seq | GSE51633 | GSM1249878 | HEK293T | Gro-seq-siBrd4-2-re2-HEK293T |
| GRO-seq | GSE52642 | GSM1273483 | HUVEC | HUVEC-GRO-Seq-Notx-2h-rep1 |
| GRO-seq | GSE52642 | GSM1273484 | HUVEC | HUVEC-GRO-Seq-VEGFA-2h-rep1 |
| GRO-seq | GSE52642 | GSM1273485 | HUVEC | HAEC-GRO-Seq-Notx-2h |
| GRO-seq | GSE52642 | GSM1273486 | HUVEC | HAEC-GRO-Seq-VEGFA-2h |
| GRO-seq | GSE52935 | GSM1278354 | A549 | siCtrl-GRO-t0 |
| GRO-seq | GSE52935 | GSM1278355 | A549 | siCtrl-GRO-t4 |
| GRO-seq | GSE52935 | GSM1278356 | A549 | siSetx-GRO-t0 |
| GRO-seq | GSE52935 | GSM1278357 | A549 | siSetx-GRO-t4 |
| GRO-seq | GSE54946 | GSM1327095 | VCaP | 0h_GRO_seq |
| GRO-seq | GSE54946 | GSM1327096 | VCaP | 2h_GRO_seq |
| GRO-seq | GSE55903 | GSM1348226 | LNCaP | plusDHT_DMSO_hs47l8_1 |
| GRO-seq | GSE55903 | GSM1348227 | LNCaP | minusDHT_DMSO_hs47l8_2 |
| GRO-seq | GSE55903 | GSM1348228 | LNCaP | plusDHT_SD70_hs47l8_3 |

| | | | | |
|---|---|---|---|---|
| GRO-seq | GSE55903 | GSM1348229 | LNCaP | minusDHT_SD70_hs47l8_4 |
| GRO-seq | GSE57430 | GSM1382433 | A375 | 48h DMSO treated A375 cells replicate a |
| GRO-seq | GSE57430 | GSM1382434 | A375 | 48h DMSO treated A375 cells replicate b |
| GRO-seq | GSE57430 | GSM1382435 | A375 | 48h Leflunomide treated A375 cells replicate a |
| GRO-seq | GSE57430 | GSM1382436 | A375 | 48h Leflunomide treated A375 cells replicate b |
| GRO-seq | GSE57430 | GSM1382437 | A375 | 48h A771726 treated A375 cells replicate a |
| GRO-seq | GSE57430 | GSM1382438 | A375 | 48h A771726 treated A375 cells replicate b |
| GRO-seq | GSE40632 | GSM1405106 | HeLa | GRO_CTRL_EGF_rep1 |
| GRO-seq | GSE40632 | GSM1405107 | HeLa | GRO_DOX_EGF_rep1 |
| GRO-seq | GSE40632 | GSM1405108 | HeLa | GRO_CTRL_EGF_rep2 |
| GRO-seq | GSE40632 | GSM1405109 | HeLa | GRO_DOX_EGF_rep2 |
| GRO-seq | GSE52642 | GSM1412749 | HUVEC | HUVEC-GRO-Seq-Notx-2h-rep2 |
| GRO-seq | GSE52642 | GSM1412750 | HUVEC | HUVEC-GRO-Seq-VEGFA-2h-rep2 |
| GRO-seq | GSE59531 | GSM1438934 | MCF7 | GRO-seq_Veh_40m_rep1 |
| GRO-seq | GSE59531 | GSM1438935 | MCF7 | GRO-seq_Veh_40m_rep2 |
| GRO-seq | GSE59531 | GSM1438936 | MCF7 | GRO-seq_Veh_40m_rep3 |
| GRO-seq | GSE59531 | GSM1438937 | MCF7 | GRO-seq_E2_40m_rep1 |
| GRO-seq | GSE59531 | GSM1438938 | MCF7 | GRO-seq_E2_40m_rep2 |
| GRO-seq | GSE59531 | GSM1438939 | MCF7 | GRO-seq_E2_40m_rep3 |
| GRO-seq | GSE59531 | GSM1438940 | MCF7 | GRO-seq_TNFŒ±_40m_rep1 |
| GRO-seq | GSE59531 | GSM1438941 | MCF7 | GRO-seq_TNFŒ±_40m_rep2 |
| GRO-seq | GSE59531 | GSM1438942 | MCF7 | GRO-seq_TNFŒ±_40m_rep3 |
| GRO-seq | GSE59531 | GSM1438943 | MCF7 | GRO-seq_E2+TNFŒ±_40m_rep1 |
| GRO-seq | GSE59531 | GSM1438944 | MCF7 | GRO-seq_E2+TNFŒ±_40m_rep2 |
| GRO-seq | GSE59531 | GSM1438945 | MCF7 | GRO-seq_E2+TNFŒ±_40m_rep3 |
| GRO-seq | GSE60271 | GSM1470027 | MCF7 | GROSeq_siCTL_Veh_Exp1 |
| GRO-seq | GSE60271 | GSM1470028 | MCF7 | GROSeq_siCTL_E2_Exp1 |
| GRO-seq | GSE60271 | GSM1470029 | MCF7 | GROSeq_siGATA3_Veh_Exp1 |
| GRO-seq | GSE60271 | GSM1470030 | MCF7 | GROSeq_siGATA3_E2_Exp1 |
| GRO-seq | GSE60271 | GSM1470031 | MCF7 | GROSeq_shCTL_Veh_Exp2 |
| GRO-seq | GSE60271 | GSM1470032 | MCF7 | GROSeq_shCTL_RA_Exp2 |
| GRO-seq | GSE60271 | GSM1470033 | MCF7 | GROSeq_shCTL_E2_Exp2 |
| GRO-seq | GSE60271 | GSM1470034 | MCF7 | GROSeq_shRARs_Veh_Exp2 |
| GRO-seq | GSE60271 | GSM1470035 | MCF7 | GROSeq_shRARs_RA_Exp2 |
| GRO-seq | GSE60271 | GSM1470036 | MCF7 | GROSeq_shRARs_E2_Exp2 |
| GRO-seq | GSE60271 | GSM1470037 | MCF7 | GROSeq_shCTL_Veh_Exp3 |
| GRO-seq | GSE60271 | GSM1470038 | MCF7 | GROSeq_shCTL_E2_Exp3 |
| GRO-seq | GSE60271 | GSM1470039 | MCF7 | GROSeq_shAP2g_Veh_Exp3 |
| GRO-seq | GSE60271 | GSM1470040 | MCF7 | GROSeq_shAP2g_E2_Exp3 |
| GRO-seq | GSE60454 | GSM1480325 | K562 | K562 GRO-seq |
| GRO-seq | GSE60454 | GSM1480326 | GM12878 | GM12878 GRO-seq |
| GRO-seq | GSE62046 | GSM1518913 | HeLa | HeLa GRO-Seq rep1 |
| GRO-seq | GSE62046 | GSM1518914 | HeLa | HeLa GRO-Seq rep2 |
| GRO-seq | GSE62228 | GSM1523191 | MCF7 | EtOH-siCTL_repeat1_GROseq (v.s. EtOH-siNCAPG_GROseq) |
| GRO-seq | GSE62228 | GSM1523192 | MCF7 | E2-siCTL_repeat1_GROseq (v.s. E2-siNCAPG_GROseq) |
| GRO-seq | GSE62228 | GSM1523193 | MCF7 | EtOH-siNCAPG_GROseq |
| GRO-seq | GSE62228 | GSM1523194 | MCF7 | E2-siNCAPG_GROseq |
| GRO-seq | GSE62228 | GSM1523195 | MCF7 | EtOH-siCTL_repeat2_GROseq (v.s. EtOH-siNCAPD3_GROseq) |
| GRO-seq | GSE62228 | GSM1523196 | MCF7 | E2-siCTL_repeat2_GROseq (v.s. E2-siNCAPD3_GROseq) |
| GRO-seq | GSE62228 | GSM1523197 | MCF7 | EtOH-siNCAPD3_GROseq |
| GRO-seq | GSE62228 | GSM1523198 | MCF7 | E2-siNCAPD3_GROseq |
| GRO-seq | GSE63202 | GSM1543777 | LNCaP | LNCaP_GRO-seq_siControl_1h_vehicle_replicate-A |
| GRO-seq | GSE63202 | GSM1543778 | LNCaP | LNCaP_GRO-seq_siControl_1h_dht_replicate-A |
| GRO-seq | GSE63202 | GSM1543779 | LNCaP | LNCaP_GRO-seq_siTOP1_1h_vehicle_replicate-A |
| GRO-seq | GSE63202 | GSM1543780 | LNCaP | LNCaP_GRO-seq_siTOP1_1h_dht_replicate-A |
| GRO-seq | GSE63202 | GSM1543781 | LNCaP | LNCaP_GRO-seq_siControl_1h_vehicle_replicate-B |
| GRO-seq | GSE63202 | GSM1543782 | LNCaP | LNCaP_GRO-seq_siControl_1h_dht_replicate-B |
| GRO-seq | GSE63202 | GSM1543783 | LNCaP | LNCaP_GRO-seq_siMRE11_1h_vehicle_replicate-B |
| GRO-seq | GSE63202 | GSM1543784 | LNCaP | LNCaP_GRO-seq_siMRE11_1h_dht_replicate-B |
| GRO-seq | GSE63202 | GSM1543785 | LNCaP | LNCaP_GRO-seq_siNKX3.1_1h_vehicle_replicate-A |
| GRO-seq | GSE63202 | GSM1543786 | LNCaP | LNCaP_GRO-seq_siNKX3.1_1h_dht_replicate-A |
| GRO-seq | GSE63202 | GSM1543787 | LNCaP | LNCaP_GRO-seq_siControl_1h_vehicle_replicate-C |
| GRO-seq | GSE63202 | GSM1543788 | LNCaP | LNCaP_GRO-seq_siControl_1h_dht_replicate-C |
| GRO-seq | GSE63202 | GSM1543789 | LNCaP | LNCaP_GRO-seq_siMRE11_1h_vehicle_replicate-C |
| GRO-seq | GSE63202 | GSM1543790 | LNCaP | LNCaP_GRO-seq_siMRE11_1h_dht_replicate-C |
| GRO-seq | GSE63202 | GSM1543796 | LNCaP | LNCaP_GRO-seq_siControl_1h_vehicle_replicate-D |
| GRO-seq | GSE63202 | GSM1543797 | LNCaP | LNCaP_GRO-seq_siControl_1h_dht_replicate-D |
| GRO-seq | GSE63202 | GSM1543798 | LNCaP | LNCaP_GRO-seq_siTOP1_1h_vehicle_replicate-D |
| GRO-seq | GSE63202 | GSM1543799 | LNCaP | LNCaP_GRO-seq_siTOP1_1h_dht_replicate-D |
| GRO-seq | GSE63375 | GSM1553207 | HEK293 | Rpb1 WT GRO-seq control |
| GRO-seq | GSE63375 | GSM1553208 | HEK293 | Rpb1 WT GRO-seq DRB3hr |
| GRO-seq | GSE63375 | GSM1553209 | HEK293 | Rpb1 WT GRO-seq washout 10' |
| GRO-seq | GSE63375 | GSM1553210 | HEK293 | Rpb1 WT GRO-seq washout 20' |
| GRO-seq | GSE63375 | GSM1553211 | HEK293 | Rpb1 R749H GRO-seq control |
| GRO-seq | GSE63375 | GSM1553212 | HEK293 | Rpb1 R749H GRO-seq DRB3hr |
| GRO-seq | GSE63375 | GSM1553213 | HEK293 | Rpb1 R749H GRO-seq washout 10' |
| GRO-seq | GSE63375 | GSM1553214 | HEK293 | Rpb1 R749H GRO-seq washout 20' |
| GRO-seq | GSE63375 | GSM1553215 | HEK293 | Rpb1 H1106Y GRO-seq control |
| GRO-seq | GSE63375 | GSM1553216 | HEK293 | Rpb1 H1106Y GRO-seq DRB3hr |

| | | | | |
|---|---|---|---|---|
| GRO-seq | GSE63375 | GSM1553217 | HEK293 | Rpb1 H1106Y GRO-seq washout 10' |
| GRO-seq | GSE63375 | GSM1553218 | HEK293 | Rpb1 H1106Y GRO-seq washout 20' |
| GRO-seq | GSE63375 | GSM1553219 | HEK293 | Rpb1 E1126G GRO-seq control |
| GRO-seq | GSE63375 | GSM1553220 | HEK293 | Rpb1 E1126G GRO-seq DRB3hr |
| GRO-seq | GSE63375 | GSM1553221 | HEK293 | Rpb1 E1126G GRO-seq washout 10' |
| GRO-seq | GSE63375 | GSM1553222 | HEK293 | Rpb1 E1126G GRO-seq washout 20' |
| GRO-seq | GSE63375 | GSM1553223 | HEK293 | Rpb1 R749H RASL-seq Rep1 |
| GRO-seq | GSE63375 | GSM1553224 | HEK293 | Rpb1 R749H RASL-seq Rep2 |
| GRO-seq | GSE63375 | GSM1553225 | HEK293 | Rpb1 R749H RASL-seq Rep3 |
| GRO-seq | GSE63375 | GSM1553226 | HEK293 | Rpb1 E1126G RASL-seq Rep1 |
| GRO-seq | GSE63375 | GSM1553227 | HEK293 | Rpb1 E1126G RASL-seq Rep2 |
| GRO-seq | GSE63375 | GSM1553228 | HEK293 | Rpb1 E1126G RASL-seq Rep3 |
| GRO-seq | GSE63375 | GSM1553229 | HEK293 | Rpb1 H1106Y RASL-seq Rep1 |
| GRO-seq | GSE63375 | GSM1553230 | HEK293 | Rpb1 H1106Y RASL-seq Rep2 |
| GRO-seq | GSE63375 | GSM1553231 | HEK293 | Rpb1 H1106Y RASL-seq Rep3 |
| GRO-seq | GSE63375 | GSM1553232 | HEK293 | Rpb1 WT RASL-seq Rep1 |
| GRO-seq | GSE63375 | GSM1553233 | HEK293 | Rpb1 WT RASL-seq Rep2 |
| GRO-seq | GSE63375 | GSM1553234 | HEK293 | Rpb1 WT RASL-seq Rep3 |
| GRO-seq | GSE64758 | GSM1579367 | H1-hESC | GROseq untreated (+ chain) |
| GRO-seq | GSE64758 | GSM1579368 | H1-hESC | GROseq untreated (- chain) |
| GRO-seq | GSE64758 | GSM1579369 | H1-hESC | GROseq Wnt3a (+ chain) |
| GRO-seq | GSE64758 | GSM1579370 | H1-hESC | GROseq Wnt3a (- chain) |
| GRO-seq | GSE64758 | GSM1579371 | H1-hESC | GROseq Wnt3a+Activin (+ chain) |
| GRO-seq | GSE64758 | GSM1579372 | H1-hESC | GROseq Wnt3a+Activin (- chain) |
| GRO-seq | GSE64758 | GSM1579373 | H1-hESC | GROseq Activin (+ chain) |
| GRO-seq | GSE64758 | GSM1579374 | H1-hESC | GROseq Activin (- chain) |
| GRO-seq | GSE64758 | GSM1579375 | H1-hESC | GROseq siC untreated (+ chain) |
| GRO-seq | GSE64758 | GSM1579376 | H1-hESC | GROseq siC untreated (- chain) |
| GRO-seq | GSE64758 | GSM1579377 | H1-hESC | GROseq siC Wnt3a+Activin (+ chain) |
| GRO-seq | GSE64758 | GSM1579378 | H1-hESC | GROseq siC Wnt3a+Activin (- chain) |
| GRO-seq | GSE64758 | GSM1579379 | H1-hESC | GROseq siYAP untreated (+ chain) |
| GRO-seq | GSE64758 | GSM1579380 | H1-hESC | GROseq siYAP untreated (- chain) |
| GRO-seq | GSE64758 | GSM1579381 | H1-hESC | GROseq siYAP Wnt3a+Activin (+ chain) |
| GRO-seq | GSE64758 | GSM1579382 | H1-hESC | GROseq siYAP Wnt3a+Activin (- chain) |
| GRO-seq | GSE66448 | GSM1622612 | K562 | K562-GROseq-C-rep1 |
| GRO-seq | GSE66448 | GSM1622613 | K562 | K562-GROseq-C-rep2 |
| GRO-seq | GSE66448 | GSM1622614 | K562 | K562-GROseq-HS-rep1 |
| GRO-seq | GSE66448 | GSM1622615 | K562 | K562-GROseq-Hs-rep2 |
| GRO-seq | GSE66928 | GSM1634453 | U2OS | C.gro.rep1 |
| GRO-seq | GSE66928 | GSM1634454 | U2OS | Myc.gro.rep1 |
| GRO-seq | GSE66928 | GSM1634455 | U2OS | C.gro.rep2 |
| GRO-seq | GSE66928 | GSM1634456 | U2OS | Myc.gro.rep2 |
| GRO-seq | GSE67295 | GSM1643990 | MCF7 | Gro-Seq Veh Rep1 |
| GRO-seq | GSE67295 | GSM1643991 | MCF7 | Gro-Seq Veh Rep2 |
| GRO-seq | GSE67295 | GSM1643992 | MCF7 | Gro-Seq E2 Rep1 |
| GRO-seq | GSE67295 | GSM1643993 | MCF7 | Gro-Seq E2 Rep2 |
| GRO-seq | GSE67295 | GSM1643994 | MCF7 | Gro-Seq IL1b Rep1 |
| GRO-seq | GSE67295 | GSM1643995 | MCF7 | Gro-Seq IL1b Rep2 |
| GRO-seq | GSE67295 | GSM1643996 | MCF7 | Gro-Seq TNFa Rep1 |
| GRO-seq | GSE67295 | GSM1643997 | MCF7 | Gro-Seq TNFa Rep2 |
| GRO-seq | GSE67519 | GSM1648604 | Nalm6 | Nalm6-LUC 24h GRO-seq rep1 |
| GRO-seq | GSE67519 | GSM1648605 | Nalm6 | Nalm6-LUC 24h GRO-seq rep2 |
| GRO-seq | GSE67519 | GSM1648606 | Nalm6 | Nalm6-TA 0h GRO-seq rep1 |
| GRO-seq | GSE67519 | GSM1648607 | Nalm6 | Nalm6-TA 0h GRO-seq rep2 |
| GRO-seq | GSE67519 | GSM1648608 | Nalm6 | Nalm6-TA 4h GRO-seq rep1 |
| GRO-seq | GSE67519 | GSM1648609 | Nalm6 | Nalm6-TA 4h GRO-seq rep2 |
| GRO-seq | GSE67519 | GSM1648610 | Nalm6 | Nalm6-TA 12h GRO-seq rep1 |
| GRO-seq | GSE67519 | GSM1648611 | Nalm6 | Nalm6-TA 12h GRO-seq rep2 |
| GRO-seq | GSE67519 | GSM1648612 | Nalm6 | Nalm6-TA 24h GRO-seq rep1 |
| GRO-seq | GSE67519 | GSM1648613 | Nalm6 | Nalm6-TA 24h GRO-seq rep2 |
| GRO-seq | GSE67519 | GSM1648614 | Nalm6 | Nalm6-TAmut 24h GRO-seq rep1 |
| GRO-seq | GSE67519 | GSM1648615 | Nalm6 | Nalm6-TAmut 24h GRO-seq rep2 |
| GRO-seq | GSE67540 | GSM1649153 | Nalm6 | Nalm6 cell line [Gro-Seq] |
| GRO-seq | GSE67540 | GSM1649154 | Nalm6 | Nalm6 cell line co-cultured with stromal cells [Gro-Seq] |
| GRO-seq | GSE67540 | GSM1649155 | REH | REH cell line_1 [Gro-Seq] |
| GRO-seq | GSE67540 | GSM1649156 | REH | REH cell line_2 [Gro-Seq] |
| GRO-seq | GSE67540 | GSM1649157 | REH | REH cell line_3 [Gro-Seq] |
| GRO-seq | GSE67540 | GSM1649158 | REH | REH cell line co-cultured with stromal cells_1 [Gro-Seq] |
| GRO-seq | GSE67540 | GSM1649159 | REH | REH cell line co-cultured with stromal cells_2 [Gro-Seq] |
| GRO-seq | GSE67540 | GSM1649160 | REH | REH cell line co-cultured with stromal cells_3 [Gro-Seq] |
| GRO-seq | GSE67540 | GSM1649161 | REH | REH cell line co-cultured with differentiated (A) stromal cells [Gro-Seq] |
| GRO-seq | GSE67540 | GSM1649162 | REH | REH cell line co-cultured with differentiated (O) stromal cells [Gro-Seq] |
| GRO-seq | GSE69757 | GSM1708338 | HeLa | Gro-seq-siCTL-HeLa |
| GRO-seq | GSE69757 | GSM1708339 | HeLa | Gro-seq-siYY1-HeLa |
| GRO-seq | GSE70408 | GSM1727118 | HCT116 | GRO.seq_shPAF1.1_HCT116_rep1 |
| GRO-seq | GSE70408 | GSM1727119 | HCT116 | GRO.seq_shPAF1.1_HCT116_rep2 |
| GRO-seq | GSE70408 | GSM1727120 | HCT116 | GRO.seq_shSCR_HCT116_rep1 |
| GRO-seq | GSE70408 | GSM1727121 | HCT116 | GRO.seq_shSCR_HCT116_rep2 |
| GRO-seq | GSE71898 | GSM1847251 | MCF7 | MCF7_Akti1/2_Replicate1 (GRO-Seq) |

| | | | | |
|---|---|---|---|---|
| GRO-seq | GSE71898 | GSM1847252 | MCF7 | MCF7_Akti1/2_Replicate2 (GRO-Seq) |
| GRO-seq | GSE71898 | GSM1847253 | MCF7 | MCF7_DMSO_Replicate1 (GRO-Seq) |
| GRO-seq | GSE71898 | GSM1847254 | MCF7 | MCF7_DMSO_Replicate2 (GRO-Seq) |
| GRO-seq | GSE71898 | GSM1847255 | HCT116 | HCT116_Akti1/2_Replicate1 (GRO-Seq) |
| GRO-seq | GSE71898 | GSM1847256 | HCT116 | HCT116_Akti1/2_Replicate2 (GRO-Seq) |
| GRO-seq | GSE71898 | GSM1847257 | HCT116 | HCT116_DMSO_Replicate1 (GRO-Seq) |
| GRO-seq | GSE71898 | GSM1847258 | HCT116 | HCT116_DMSO_Replicate2 (GRO-Seq) |
| GRO-seq | GSE74142 | GSM1911184 | MCF7 | GRO-seq_LucKD_Veh_MCF-7_1 |
| GRO-seq | GSE74142 | GSM1911185 | MCF7 | GRO-seq_LucKD_Veh_MCF-7_2 |
| GRO-seq | GSE74142 | GSM1911186 | MCF7 | GRO-seq_PARP-1KD_Veh_MCF7_1 |
| GRO-seq | GSE74142 | GSM1911187 | MCF7 | GRO-seq_PARP-1KD_Veh_MCF7_2 |
| GRO-seq | GSE74142 | GSM2151684 | MCF7 | GRO-seq_LucKD_PJ34_MCF7 |
| GRO-seq | GSE81511 | GSM2155000 | MCF7 | Gro-Seq plus ICI |
| GRO-seq | GSE81511 | GSM2155001 | MCF7 | Gro-Seq plus ICI and Dex |
| GRO-seq | GSE81511 | GSM2155002 | MCF7 | Gro-Seq plus E2 |
| GRO-seq | GSE81511 | GSM2155003 | MCF7 | Gro-Seq plus E2 and Dex |
| GRO-seq | GSE82312 | GSM2188872 | MDA-MB-231 | GROseq.MDA231_DMSO_1 |
| GRO-seq | GSE82312 | GSM2188873 | MDA-MB-231 | GROseq.MDA231_DMSO_2 |
| GRO-seq | GSE82312 | GSM2188874 | MDA-MB-231 | GROseq.MDA231_JQ1_1 |
| GRO-seq | GSE82312 | GSM2188875 | MDA-MB-231 | GROseq.MDA231_JQ1_2 |
| GRO-seq | GSE84432 | GSM2235679 | VCaP | VCaP_GROseq_EtOH_rep1 |
| GRO-seq | GSE84432 | GSM2235680 | VCaP | VCaP_GROseq_EtOH_rep2 |
| GRO-seq | GSE84432 | GSM2235681 | VCaP | VCaP_GROseq_R1881_30min_rep1 |
| GRO-seq | GSE84432 | GSM2235682 | VCaP | VCaP_GROseq_R1881_30min_rep2 |
| GRO-seq | GSE84432 | GSM2235683 | VCaP | VCaP_GROseq_R1881_2h_rep1 |
| GRO-seq | GSE84432 | GSM2235684 | VCaP | VCaP_GROseq_R1881_2h_rep2 |
| GRO-seq | GSE84432 | GSM2262426 | VCaP | VCaP_GROseq_EtOH_for_DHT_rep1 |
| GRO-seq | GSE84432 | GSM2262427 | VCaP | VCaP_GROseq_EtOH_for_DHT_rep2 |
| GRO-seq | GSE84432 | GSM2262428 | VCaP | VCaP_GROseq_DHT_4h_rep1 |
| GRO-seq | GSE84432 | GSM2262429 | VCaP | VCaP_GROseq_DHT_4h_rep2 |
| GRO-seq | GSE86165 | GSM2296620 | HCT116 | HCT116, GRO-Seq, DMSO treated, replicate 1 |
| GRO-seq | GSE86165 | GSM2296622 | HCT116 | HCT116, GRO-Seq, DMSO treated, replicate 2 |
| GRO-seq | GSE86165 | GSM2296625 | HCT116 | HCT116, GRO-Seq, Nutlin treated, replicate 1 |
| GRO-seq | GSE86165 | GSM2296633 | HCT116 | HCT116, GRO-Seq, Nutlin treated, replicate 2 |
| GRO-seq | GSE86165 | GSM2296635 | MCF7 | MCF7, GRO-Seq, DMSO treated, replicate 1 |
| GRO-seq | GSE86165 | GSM2296637 | MCF7 | MCF7, GRO-Seq, DMSO treated, replicate 2 |
| GRO-seq | GSE86165 | GSM2296640 | MCF7 | MCF7, GRO-Seq, Nutlin treated, replicate 1 |
| GRO-seq | GSE86165 | GSM2296641 | MCF7 | MCF7, GRO-Seq, Nutlin treated, replicate 2 |
| GRO-seq | GSE86165 | GSM2296643 | SJSA | SJSA, GRO-Seq, DMSO treated, replicate 1 |
| GRO-seq | GSE86165 | GSM2296646 | SJSA | SJSA, GRO-Seq, DMSO treated, replicate 2 |
| GRO-seq | GSE86165 | GSM2296647 | SJSA | SJSA, GRO-Seq, Nutlin treated, replicate 1 |
| GRO-seq | GSE86165 | GSM2296648 | SJSA | SJSA, GRO-Seq, Nutlin treated, replicate 2 |
| GRO-seq | GSE91010 | GSM2419218 | MRC5VA | UV-.t10 |
| GRO-seq | GSE91010 | GSM2419219 | MRC5VA | UV-t25 |
| GRO-seq | GSE91010 | GSM2419220 | MRC5VA | UV-.t40 |
| GRO-seq | GSE91010 | GSM2419221 | MRC5VA | UV+.t10 |
| GRO-seq | GSE91010 | GSM2419222 | MRC5VA | UV+.t25 |
| GRO-seq | GSE91010 | GSM2419223 | MRC5VA | UV+.t40 |
| GRO-seq | GSE91011 | GSM2419224 | MRC5VA | Untreated |
| GRO-seq | GSE91011 | GSM2419225 | MRC5VA | UV-2h |
| GRO-seq | GSE91011 | GSM2419226 | MRC5VA | UV-5h |
| GRO-seq | GSE91011 | GSM2419227 | MRC5VA | UV-8h |
| GRO-seq | GSE91011 | GSM2419228 | MRC5VA | UV-10h |
| GRO-seq | GSE91011 | GSM2419229 | MRC5VA | UV-12h |
| GRO-seq | GSE91011 | GSM2419230 | MRC5VA | UV-24h |
| GRO-seq | GSE90035 | GSM2448893 | MCF7 | GRO-seq control sample |
| GRO-seq | GSE94479 | GSM2476252 | MCF7 | MCF7 GRO-seq G0/G1 |
| GRO-seq | GSE94479 | GSM2476253 | MCF7 | MCF7 GRO-seq S phase |
| GRO-seq | GSE94479 | GSM2476254 | MCF7 | MCF7 GRO-seq M phase |
| GRO-seq | GSE94872 | GSM2486801 | HUVEC | HUVEC GRO-seq normoxia replicate 1 |
| GRO-seq | GSE94872 | GSM2486802 | HUVEC | HUVEC GRO-seq normoxia replicate 2 |
| GRO-seq | GSE94872 | GSM2486803 | HUVEC | HUVEC GRO-seq normoxia replicate 3 |
| GRO-seq | GSE94872 | GSM2486804 | HUVEC | HUVEC GRO-seq normoxia replicate 4 |
| GRO-seq | GSE94872 | GSM2486805 | HUVEC | HUVEC GRO-seq hypoxia replicate 1 |
| GRO-seq | GSE94872 | GSM2486806 | HUVEC | HUVEC GRO-seq hypoxia replicate 2 |
| GRO-seq | GSE94872 | GSM2486807 | HUVEC | HUVEC GRO-seq hypoxia replicate 3 |
| GRO-seq | GSE94872 | GSM2486808 | HUVEC | HUVEC GRO-seq hypoxia replicate 4 |
| GRO-seq | GSE95122 | GSM2496535 | MDA-MB-231 | GRO-seq_231ERŒ±WT_0min_rep1 |
| GRO-seq | GSE95122 | GSM2496536 | MDA-MB-231 | GRO-seq_231ERŒ±WT_0min_rep2 |
| GRO-seq | GSE95122 | GSM2496537 | MDA-MB-231 | GRO-seq_231ERŒ±WT_0min_rep3 |
| GRO-seq | GSE95122 | GSM2496538 | MDA-MB-231 | GRO-seq_231ERŒ±WT_0min_rep4 |
| GRO-seq | GSE95122 | GSM2496539 | MDA-MB-231 | GRO-seq_231ERŒ±WT_20min_rep1 |
| GRO-seq | GSE95122 | GSM2496540 | MDA-MB-231 | GRO-seq_231ERŒ±WT_20min_rep2 |
| GRO-seq | GSE95122 | GSM2496541 | MDA-MB-231 | GRO-seq_231ERŒ±WT_20min_rep3 |
| GRO-seq | GSE95122 | GSM2496542 | MDA-MB-231 | GRO-seq_231ERŒ±WT_20min_rep4 |
| GRO-seq | GSE95122 | GSM2496543 | MDA-MB-231 | GRO-seq_231ERŒ±WT_45min_rep1 |
| GRO-seq | GSE95122 | GSM2496544 | MDA-MB-231 | GRO-seq_231ERŒ±WT_45min_rep2 |
| GRO-seq | GSE95122 | GSM2496545 | MDA-MB-231 | GRO-seq_231ERŒ±WT_45min_rep3 |
| GRO-seq | GSE95122 | GSM2496546 | MDA-MB-231 | GRO-seq_231ERŒ±WT_45min_rep4 |

| | | | | |
|---|---|---|---|---|
| GRO-seq | GSE95122 | GSM2496547 | MDA-MB-231 | GRO-seq_231ERŒ±LQ_0min_rep1 |
| GRO-seq | GSE95122 | GSM2496548 | MDA-MB-231 | GRO-seq_231ERŒ±LQ_0min_rep2 |
| GRO-seq | GSE95122 | GSM2496549 | MDA-MB-231 | GRO-seq_231ERŒ±LQ_0min_rep3 |
| GRO-seq | GSE95122 | GSM2496550 | MDA-MB-231 | GRO-seq_231ERŒ±LQ_0min_rep4 |
| GRO-seq | GSE95122 | GSM2496551 | MDA-MB-231 | GRO-seq_231ERŒ±LQ_20min_rep1 |
| GRO-seq | GSE95122 | GSM2496552 | MDA-MB-231 | GRO-seq_231ERŒ±LQ_20min_rep2 |
| GRO-seq | GSE95122 | GSM2496553 | MDA-MB-231 | GRO-seq_231ERŒ±LQ_20min_rep3 |
| GRO-seq | GSE95122 | GSM2496554 | MDA-MB-231 | GRO-seq_231ERŒ±LQ_20min_rep4 |
| GRO-seq | GSE95122 | GSM2496555 | MDA-MB-231 | GRO-seq_231ERŒ±LQ_45min_rep1 |
| GRO-seq | GSE95122 | GSM2496556 | MDA-MB-231 | GRO-seq_231ERŒ±LQ_45min_rep2 |
| GRO-seq | GSE95122 | GSM2496557 | MDA-MB-231 | GRO-seq_231ERŒ±LQ_45min_rep3 |
| GRO-seq | GSE95122 | GSM2496558 | MDA-MB-231 | GRO-seq_231ERŒ±LQ_45min_rep4 |
| GRO-seq | GSE96859 | GSM2545171 | 76NF2V | 76NF2V rep1 (GRO-Seq) |
| GRO-seq | GSE96859 | GSM2545172 | 76NF2V | 76NF2V rep2 (GRO-Seq) |
| GRO-seq | GSE96859 | GSM2545173 | 76NF2V | 76NF2V rep3 (GRO-Seq) |
| GRO-seq | GSE96859 | GSM2545174 | 76NF2V | 76NF2V rep4 (GRO-Seq) |
| GRO-seq | GSE96859 | GSM2545175 | MCF10A | MCF10A rep1 (GRO-Seq) |
| GRO-seq | GSE96859 | GSM2545176 | MCF10A | MCF10A rep2 (GRO-Seq) |
| GRO-seq | GSE96859 | GSM2545177 | MCF10A | MCF10A rep3 (GRO-Seq) |
| GRO-seq | GSE96859 | GSM2545178 | MCF10A | MCF10A rep4 (GRO-Seq) |
| GRO-seq | GSE96859 | GSM2545179 | MCF7 | MCF-7 rep1 (GRO-Seq) |
| GRO-seq | GSE96859 | GSM2545180 | MCF7 | MCF-7 rep2 (GRO-Seq) |
| GRO-seq | GSE96859 | GSM2545181 | MCF7 | MCF-7 rep3 (GRO-Seq) |
| GRO-seq | GSE96859 | GSM2545182 | ZR751 | ZR751 rep1 (GRO-Seq) |
| GRO-seq | GSE96859 | GSM2545183 | ZR751 | ZR751 rep2 (GRO-Seq) |
| GRO-seq | GSE96859 | GSM2545184 | ZR751 | ZR751 rep3 (GRO-Seq) |
| GRO-seq | GSE96859 | GSM2545185 | ZR751 | ZR751 rep4 (GRO-Seq) |
| GRO-seq | GSE96859 | GSM2545186 | MDA MB-361 | MDA MB-361 rep1 (GRO-Seq) |
| GRO-seq | GSE96859 | GSM2545187 | MDA MB-361 | MDA MB-361 rep2 (GRO-Seq) |
| GRO-seq | GSE96859 | GSM2545188 | MDA MB-361 | MDA MB-361 rep3 (GRO-Seq) |
| GRO-seq | GSE96859 | GSM2545189 | MDA MB-361 | MDA MB-361 rep4 (GRO-Seq) |
| GRO-seq | GSE96859 | GSM2545190 | UACC812 | UACC812 rep1 (GRO-Seq) |
| GRO-seq | GSE96859 | GSM2545191 | UACC812 | UACC812 rep2 (GRO-Seq) |
| GRO-seq | GSE96859 | GSM2545192 | UACC812 | UACC812 rep3 (GRO-Seq) |
| GRO-seq | GSE96859 | GSM2545193 | SKBR3 | SKBR3 rep1 (GRO-Seq) |
| GRO-seq | GSE96859 | GSM2545194 | SKBR3 | SKBR3 rep2 (GRO-Seq) |
| GRO-seq | GSE96859 | GSM2545195 | SKBR3 | SKBR3 rep3 (GRO-Seq) |
| GRO-seq | GSE96859 | GSM2545196 | SKBR3 | SKBR3 rep4 (GRO-Seq) |
| GRO-seq | GSE96859 | GSM2545197 | AU565 | AU565 rep1 (GRO-Seq) |
| GRO-seq | GSE96859 | GSM2545198 | AU565 | AU565 rep2 (GRO-Seq) |
| GRO-seq | GSE96859 | GSM2545199 | AU565 | AU565 rep3 (GRO-Seq) |
| GRO-seq | GSE96859 | GSM2545200 | AU565 | AU565 rep4 (GRO-Seq) |
| GRO-seq | GSE96859 | GSM2545201 | HCC1954 | HCC1954 rep1 (GRO-Seq) |
| GRO-seq | GSE96859 | GSM2545202 | HCC1954 | HCC1954 rep2 (GRO-Seq) |
| GRO-seq | GSE96859 | GSM2545203 | HCC1954 | HCC1954 rep3 (GRO-Seq) |
| GRO-seq | GSE96859 | GSM2545204 | HCC1954 | HCC1954 rep4 (GRO-Seq) |
| GRO-seq | GSE96859 | GSM2545205 | MB468 | MB468 rep1 (GRO-Seq) |
| GRO-seq | GSE96859 | GSM2545206 | MB468 | MB468 rep2 (GRO-Seq) |
| GRO-seq | GSE96859 | GSM2545207 | MB468 | MB468 rep3 (GRO-Seq) |
| GRO-seq | GSE96859 | GSM2545208 | MB468 | MB468 rep4 (GRO-Seq) |
| GRO-seq | GSE96859 | GSM2545209 | HCC1937 | HCC1937 rep1 (GRO-Seq) |
| GRO-seq | GSE96859 | GSM2545210 | HCC1937 | HCC1937 rep2 (GRO-Seq) |
| GRO-seq | GSE96859 | GSM2545211 | HCC1937 | HCC1937 rep3 (GRO-Seq) |
| GRO-seq | GSE96859 | GSM2545212 | HCC1937 | HCC1937 rep4 (GRO-Seq) |
| GRO-seq | GSE96859 | GSM2545213 | MDA-MB-231 | MDA MB-231 rep1 (GRO-Seq) |
| GRO-seq | GSE96859 | GSM2545214 | MDA-MB-231 | MDA MB-231 rep2 (GRO-Seq) |
| GRO-seq | GSE96859 | GSM2545215 | MDA-MB-231 | MDA MB-231 rep3 (GRO-Seq) |
| GRO-seq | GSE96859 | GSM2545216 | MDA-MB-231 | MDA MB-231 rep4 (GRO-Seq) |
| GRO-seq | GSE96859 | GSM2545217 | MDA-MB-436 | MDA MB-436 rep1 (GRO-Seq) |
| GRO-seq | GSE96859 | GSM2545218 | MDA-MB-436 | MDA MB-436 rep2 (GRO-Seq) |
| GRO-seq | GSE96859 | GSM2545219 | MDA-MB-436 | MDA MB-436 rep3 (GRO-Seq) |
| GRO-seq | GSE96859 | GSM2545220 | MDA-MB-436 | MDA MB-436 rep4 (GRO-Seq) |
| GRO-seq | GSE101560 | GSM2706105 | MCF7 | Gro-seq-siCTL-CTL-MCF7 |
| GRO-seq | GSE101560 | GSM2706106 | MCF7 | Gro-seq-siCTL-E2-MCF7 |
| GRO-seq | GSE101560 | GSM2706107 | MCF7 | Gro-seq-siJMJD6-CTL-MCF7 |
| GRO-seq | GSE101560 | GSM2706108 | MCF7 | Gro-seq-siJMJD6-E2-MCF7 |
| GRO-seq | GSE101803 | GSM2716125 | CUTLL1 | GRO-Seq analysis in CUTLL1 cells, steady state (DMSO), replicate 1 |
| GRO-seq | GSE101803 | GSM2716126 | CUTLL1 | GRO-Seq analysis in CUTLL1 cells, steady state (DMSO), replicate 2 |
| GRO-seq | GSE101803 | GSM2716127 | CUTLL1 | GRO-Seq analysis in CUTLL1 cells, steady state (DMSO), replicate 3 |
| GRO-seq | GSE101803 | GSM2716128 | CUTLL1 | GRO-Seq analysis in CUTLL1 cells, 0 minutes post Œ≥SI wash, replicate 1 |
| GRO-seq | GSE101803 | GSM2716129 | CUTLL1 | GRO-Seq analysis in CUTLL1 cells, 0 minutes post Œ≥SI wash, replicate 2 |
| GRO-seq | GSE101803 | GSM2716130 | CUTLL1 | GRO-Seq analysis in CUTLL1 cells, 0 minutes post Œ≥SI wash, replicate 3 |
| GRO-seq | GSE101803 | GSM2716131 | CUTLL1 | GRO-Seq analysis in CUTLL1 cells, 5 minutes post Œ≥SI wash, replicate 1 |
| GRO-seq | GSE101803 | GSM2716132 | CUTLL1 | GRO-Seq analysis in CUTLL1 cells, 5 minutes post Œ≥SI wash, replicate 2 |
| GRO-seq | GSE101803 | GSM2716133 | CUTLL1 | GRO-Seq analysis in CUTLL1 cells, 5 minutes post Œ≥SI wash, replicate 3 |
| GRO-seq | GSE101803 | GSM2716134 | CUTLL1 | GRO-Seq analysis in CUTLL1 cells, 10 minutes post Œ≥SI wash, replicate 1 |
| GRO-seq | GSE101803 | GSM2716135 | CUTLL1 | GRO-Seq analysis in CUTLL1 cells, 10 minutes post Œ≥SI wash, replicate 2 |
| GRO-seq | GSE101803 | GSM2716136 | CUTLL1 | GRO-Seq analysis in CUTLL1 cells, 10 minutes post Œ≥SI wash, replicate 3 |
| GRO-seq | GSE101803 | GSM2716137 | CUTLL1 | GRO-Seq analysis in CUTLL1 cells, 20 minutes post Œ≥SI wash, replicate 1 |

| | | | | |
|---|---|---|---|---|
| GRO-seq | GSE101803 | GSM2716138 | CUTLL1 | GRO-Seq analysis in CUTLL1 cells, 20 minutes post Œ≥SI wash, replicate 2 |
| GRO-seq | GSE101803 | GSM2716139 | CUTLL1 | GRO-Seq analysis in CUTLL1 cells, 20 minutes post Œ≥SI wash, replicate 3 |
| GRO-seq | GSE101803 | GSM2716140 | CUTLL1 | GRO-Seq analysis in CUTLL1 cells, 40 minutes post Œ≥SI wash, replicate 1 |
| GRO-seq | GSE101803 | GSM2716141 | CUTLL1 | GRO-Seq analysis in CUTLL1 cells, 40 minutes post Œ≥SI wash, replicate 2 |
| GRO-seq | GSE101803 | GSM2716142 | CUTLL1 | GRO-Seq analysis in CUTLL1 cells, 40 minutes post Œ≥SI wash, replicate 3 |
| GRO-seq | GSE101803 | GSM2716143 | CUTLL1 | GRO-Seq analysis in CUTLL1 cells, 80 minutes post Œ≥SI wash, replicate 1 |
| GRO-seq | GSE101803 | GSM2716144 | CUTLL1 | GRO-Seq analysis in CUTLL1 cells, 80 minutes post Œ≥SI wash, replicate 2 |
| GRO-seq | GSE101803 | GSM2716145 | CUTLL1 | GRO-Seq analysis in CUTLL1 cells, 80 minutes post Œ≥SI wash, replicate 3 |
| GRO-seq | GSE101803 | GSM2716146 | CUTLL1 | GRO-Seq analysis in CUTLL1 cells, 160 minutes post Œ≥SI wash, replicate 1 |
| GRO-seq | GSE101803 | GSM2716147 | CUTLL1 | GRO-Seq analysis in CUTLL1 cells, 160 minutes post Œ≥SI wash, replicate 2 |
| GRO-seq | GSE101803 | GSM2716148 | CUTLL1 | GRO-Seq analysis in CUTLL1 cells, 160 minutes post Œ≥SI wash, replicate 3 |
| GRO-seq | GSE101803 | GSM2716149 | CUTLL1 | GRO-Seq analysis in CUTLL1 cells, 320 minutes post Œ≥SI wash, replicate 1 |
| GRO-seq | GSE101803 | GSM2716150 | CUTLL1 | GRO-Seq analysis in CUTLL1 cells, 320 minutes post Œ≥SI wash, replicate 2 |
| GRO-seq | GSE101803 | GSM2716151 | CUTLL1 | GRO-Seq analysis in CUTLL1 cells, 320 minutes post Œ≥SI wash, replicate 3 |
| GRO-seq | GSE101803 | GSM2716152 | CUTLL1 | GRO-Seq analysis in CUTLL1 cells, 640 minutes post Œ≥SI wash, replicate 1 |
| GRO-seq | GSE101803 | GSM2716153 | CUTLL1 | GRO-Seq analysis in CUTLL1 cells, 640 minutes post Œ≥SI wash, replicate 2 |
| GRO-seq | GSE101803 | GSM2716154 | CUTLL1 | GRO-Seq analysis in CUTLL1 cells, 640 minutes post Œ≥SI wash, replicate 3 |
| GRO-seq | GSE102796 | GSM2746533 | SW480 | 0 hr TNF-a GRO-seq |
| GRO-seq | GSE102796 | GSM2746534 | SW480 | 16 hr TNF-a GRO-seq |
| GRO-seq | GSE102586 | GSM2746829 | CD34+_erthrocyte | CD34+_erthrocyte_veh |
| GRO-seq | GSE102586 | GSM2746830 | CD34+_erthrocyte | CD34+_erthrocyte_T4 |
| GRO-seq | GSE102586 | GSM2746831 | CD34+_erthrocyte | CD34+_erthrocyte_veh [hs80l2s3] |
| GRO-seq | GSE102586 | GSM2746832 | CD34+_erthrocyte | CD34+_erthrocyte_T4 [hs80l2s4] |
| GRO-seq | GSE104545 | GSM2803058 | RMG-1 | GROSEQ_CTRL_REP1 |
| GRO-seq | GSE104545 | GSM2803059 | RMG-1 | GROSEQ_DOX_REP1 |
| GRO-seq | GSE104545 | GSM2806555 | RMG-1 | GROSEQ_CTRL_REP2 |
| GRO-seq | GSE104545 | GSM2806556 | RMG-1 | GROSEQ_DOX_REP2 |
| GRO-seq | GSE99539 | GSM2876115 | MCF7 | GRO-MCF7_1,6-Hexanediol and Vehicle |
| GRO-seq | GSE99539 | GSM2876116 | MCF7 | GRO-MCF7_1,6-Hexanediol and Estrogen |
| GRO-seq | GSE99539 | GSM2876117 | MCF7 | GRO-MCF7_2,5-Hexanediol and Vehicle |
| GRO-seq | GSE99539 | GSM2876118 | MCF7 | GRO-MCF7_2,5-Hexanediol and Estrogen |
| GRO-seq | GSE109690 | GSM3100195 | HeLa | HeLa WT, GRO-seq |
| GRO-seq | GSE109690 | GSM3100196 | HeLa | HeLa Clone KO1.1, GRO-seq |
| GRO-seq | GSE109690 | GSM3100197 | HeLa | HeLa Clone KO1.2, GRO-seq |
| GRO-seq | GSE115603 | GSM3184722 | MCF7 | GRO-seq, siCTL_EtOH (repeat1) MCF7 |
| GRO-seq | GSE115603 | GSM3184723 | MCF7 | GRO-seq, siCTL_E2 (repeat1) MCF7 |
| GRO-seq | GSE115603 | GSM3184724 | MCF7 | GRO-seq, siRAD21_EtOH (repeat1) MCF7 |
| GRO-seq | GSE115603 | GSM3184725 | MCF7 | GRO-seq, siRAD21_E2 (repeat1) MCF7 |
| GRO-seq | GSE115603 | GSM3184726 | MCF7 | GRO-seq, siCTL_EtOH (repeat2) MCF7 |
| GRO-seq | GSE115603 | GSM3184727 | MCF7 | GRO-seq, siCTL_E2 (repeat2) MCF7 |
| GRO-seq | GSE115603 | GSM3184728 | MCF7 | GRO-seq, siRAD21_EtOH (repeat2) MCF7 |
| GRO-seq | GSE115603 | GSM3184729 | MCF7 | GRO-seq, siRAD21_E2 (repeat2) MCF7 |
| GRO-seq | GSE115603 | GSM3184730 | MCF7 | GRO-seq, siCTL_EtOH (repeat3) MCF7 |
| GRO-seq | GSE115603 | GSM3184731 | MCF7 | GRO-seq, siCTL_E2 (repeat3) MCF7 |
| GRO-seq | GSE115603 | GSM3184732 | MCF7 | GRO-seq, siRAD21_EtOH (repeat3) MCF7 |
| GRO-seq | GSE115603 | GSM3184733 | MCF7 | GRO-seq, siRAD21_E2 (repeat3) MCF7 |
| GRO-seq | GSE115603 | GSM3184734 | MCF7 | GRO-seq, Wildtype MCF7_EtOH |
| GRO-seq | GSE115603 | GSM3184735 | MCF7 | GRO-seq, Wildtype MCF7_E2 |
| GRO-seq | GSE115603 | GSM3184736 | MCF7 | GRO-seq, TFF1p-KO MCF7_clone28_EtOH |
| GRO-seq | GSE115603 | GSM3184737 | MCF7 | GRO-seq, TFF1p-KO MCF7_clone28_E2 |
| GRO-seq | GSE115603 | GSM3184738 | MCF7 | GRO-seq, TFF1p-KO MCF7_clone25_EtOH |
| GRO-seq | GSE115603 | GSM3184739 | MCF7 | GRO-seq, TFF1p-KO MCF7_clone25_E2 |
| GRO-seq | GSE115894 | GSM3192671 | CUTLL1 | CUTLL1 DMSO rep1 (GRO-seq) |
| GRO-seq | GSE115894 | GSM3192672 | CUTLL1 | CUTLL1 DMSO rep2 (GRO-seq) |
| GRO-seq | GSE115894 | GSM3192673 | CUTLL1 | CUTLL1 00h rep1 (GRO-seq) |
| GRO-seq | GSE115894 | GSM3192674 | CUTLL1 | CUTLL1 00h rep2 (GRO-seq) |
| GRO-seq | GSE115894 | GSM3192675 | CUTLL1 | CUTLL1 01h rep1 (GRO-seq) |
| GRO-seq | GSE115894 | GSM3192676 | CUTLL1 | CUTLL1 01h rep2 (GRO-seq) |
| GRO-seq | GSE115894 | GSM3192677 | CUTLL1 | CUTLL1 02h rep1 (GRO-seq) |
| GRO-seq | GSE115894 | GSM3192678 | CUTLL1 | CUTLL1 02h rep2 (GRO-seq) |
| GRO-seq | GSE115894 | GSM3192679 | CUTLL1 | CUTLL1 03h rep1 (GRO-seq) |
| GRO-seq | GSE115894 | GSM3192680 | CUTLL1 | CUTLL1 03h rep2 (GRO-seq) |
| GRO-seq | GSE115894 | GSM3192681 | CUTLL1 | CUTLL1 04h rep1 (GRO-seq) |
| GRO-seq | GSE115894 | GSM3192682 | CUTLL1 | CUTLL1 04h rep2 (GRO-seq) |
| GRO-seq | GSE115894 | GSM3192683 | CUTLL1 | CUTLL1 05h rep1 (GRO-seq) |
| GRO-seq | GSE115894 | GSM3192684 | CUTLL1 | CUTLL1 05h rep2 (GRO-seq) |
| GRO-seq | GSE115894 | GSM3192685 | CUTLL1 | CUTLL1 06h rep1 (GRO-seq) |
| GRO-seq | GSE115894 | GSM3192686 | CUTLL1 | CUTLL1 06h rep2 (GRO-seq) |
| GRO-seq | GSE115894 | GSM3192687 | CUTLL1 | CUTLL1 08h rep1 (GRO-seq) |
| GRO-seq | GSE115894 | GSM3192688 | CUTLL1 | CUTLL1 08h rep2 (GRO-seq) |
| GRO-seq | GSE115894 | GSM3192689 | CUTLL1 | CUTLL1 10h rep1 (GRO-seq) |
| GRO-seq | GSE115894 | GSM3192690 | CUTLL1 | CUTLL1 10h rep2 (GRO-seq) |
| GRO-seq | GSE99544 | GSM3301936 | MCF7 | Gro-Seq minus TNFa |
| GRO-seq | GSE99544 | GSM3301937 | MCF7 | Gro-Seq plus TNFa |
| GRO-seq | GSE99544 | GSM3301938 | MCF7 | Gro-Seq minus TNFa + 1,6-Hexanediol |
| GRO-seq | GSE99544 | GSM3301939 | MCF7 | Gro-Seq plus TNFa + 1,6-Hexanediol |
| GRO-seq | GSE99544 | GSM3301940 | MCF7 | Groseq-minus E2 + 2,5-HD |
| GRO-seq | GSE99544 | GSM3301941 | MCF7 | Groseq-plus E2 + 2,5-HD |
| GRO-seq | GSE99544 | GSM3301942 | MCF7 | Groseq-plus E2 + 1,6-HD |

| | | | | |
|---|---|---|---|---|
| GRO-seq | GSE118530 | GSM3332057 | HUVEC | GRO-Seq_HUVEC_AdCMV |
| GRO-seq | GSE118530 | GSM3332058 | HUVEC | GRO-Seq_HUVEC_AdHIF1a |
| GRO-seq | GSE118530 | GSM3332059 | HUVEC | GRO-Seq_HUVEC_AdHIF2a |
| GRO-seq | GSE120105 | GSM3393668 | HepG2 | Control_GRO_rep1_HepG2_batch1 |
| GRO-seq | GSE120105 | GSM3393669 | HepG2 | Control_GRO_rep2_HepG2_batch1 |
| GRO-seq | GSE120105 | GSM3393670 | HepG2 | AGO1.KD_GRO_rep1_HepG2_batch1 |
| GRO-seq | GSE120105 | GSM3393671 | HepG2 | AGO1.KD_GRO_rep2_HepG2_batch1 |
| GRO-seq | GSE120105 | GSM3393672 | HepG2 | AGO2.KD_GRO_rep1_HepG2_batch1 |
| GRO-seq | GSE120105 | GSM3393673 | HepG2 | AGO2.KD_GRO_rep2_HepG2_batch1 |
| GRO-seq | GSE120105 | GSM3393674 | HepG2 | HNRNPL.KD_GRO_rep1_HepG2_batch1 |
| GRO-seq | GSE120105 | GSM3393675 | HepG2 | HNRNPL.KD_GRO_rep2_HepG2_batch1 |
| GRO-seq | GSE120105 | GSM3393676 | HepG2 | SRSF4.KD_GRO_rep1_HepG2_batch1 |
| GRO-seq | GSE120105 | GSM3393677 | HepG2 | SRSF4.KD_GRO_rep2_HepG2_batch1 |
| GRO-seq | GSE120105 | GSM3393678 | HepG2 | U2AF1.KD_GRO_rep1_HepG2_batch1 |
| GRO-seq | GSE120105 | GSM3393679 | HepG2 | U2AF1.KD_GRO_rep2_HepG2_batch1 |
| GRO-seq | GSE120105 | GSM3393680 | HepG2 | U2AF2.KD_GRO_rep1_HepG2_batch1 |
| GRO-seq | GSE120105 | GSM3393681 | HepG2 | U2AF2.KD_GRO_rep2_HepG2_batch1 |
| GRO-seq | GSE120105 | GSM3393682 | HepG2 | Control_GRO_rep1_HepG2_batch2 |
| GRO-seq | GSE120105 | GSM3393683 | HepG2 | Control_GRO_rep2_HepG2_batch2 |
| GRO-seq | GSE120105 | GSM3393684 | HepG2 | HNRNPH1.KD_GRO_rep1_HepG2_batch2 |
| GRO-seq | GSE120105 | GSM3393685 | HepG2 | HNRNPH1.KD_GRO_rep2_HepG2_batch2 |
| GRO-seq | GSE120105 | GSM3393686 | HepG2 | HNRNPK.KD_GRO_rep1_HepG2_batch2 |
| GRO-seq | GSE120105 | GSM3393687 | HepG2 | HNRNPK.KD_GRO_rep2_HepG2_batch2 |
| GRO-seq | GSE120105 | GSM3393688 | HepG2 | HNRNPLL.KD_GRO_rep1_HepG2_batch2 |
| GRO-seq | GSE120105 | GSM3393689 | HepG2 | HNRNPLL.KD_GRO_rep2_HepG2_batch2 |
| GRO-seq | GSE120105 | GSM3393690 | HepG2 | PCBP2.KD_GRO_rep1_HepG2_batch2 |
| GRO-seq | GSE120105 | GSM3393691 | HepG2 | PCBP2.KD_GRO_rep2_HepG2_batch2 |
| GRO-seq | GSE120105 | GSM3393692 | HepG2 | PTBP1.KD_GRO_rep1_HepG2_batch2 |
| GRO-seq | GSE120105 | GSM3393693 | HepG2 | PTBP1.KD_GRO_rep2_HepG2_batch2 |
| GRO-seq | GSE120105 | GSM3393694 | HepG2 | RBM22.KD_GRO_rep1_HepG2_batch2 |
| GRO-seq | GSE120105 | GSM3393695 | HepG2 | RBM22.KD_GRO_rep2_HepG2_batch2 |
| GRO-seq | GSE120105 | GSM3393696 | HepG2 | RBM25.KD_GRO_rep1_HepG2_batch2 |
| GRO-seq | GSE120105 | GSM3393697 | HepG2 | RBM25.KD_GRO_rep2_HepG2_batch2 |
| GRO-seq | GSE120105 | GSM3393698 | HepG2 | XRCC5.KD_GRO_rep1_HepG2_batch2 |
| GRO-seq | GSE120105 | GSM3393699 | HepG2 | XRCC5.KD_GRO_rep2_HepG2_batch2 |
| GRO-seq | GSE120105 | GSM3393700 | HepG2 | Control_GRO_rep1_HepG2_batch3 |
| GRO-seq | GSE120105 | GSM3393701 | HepG2 | Control_GRO_rep2_HepG2_batch3 |
| GRO-seq | GSE120105 | GSM3393702 | HepG2 | YY1.KD_GRO_rep1_HepG2_batch3 |
| GRO-seq | GSE120105 | GSM3393703 | HepG2 | YY1.KD_GRO_rep2_HepG2_batch3 |
| GRO-seq | GSE120105 | GSM3393704 | HepG2 | RBM25.KD_GRO_rep1_HepG2_batch3 |
| GRO-seq | GSE120105 | GSM3393705 | HepG2 | RBM25.KD_GRO_rep2_HepG2_batch3 |
| GRO-seq | GSE13518 | GSM340901 | IMR90 | GRO-seq rep 1 |
| GRO-seq | GSE13518 | GSM340902 | IMR90 | GRO-seq rep2 |
| GRO-seq | GSE99544 | GSM3555217 | IMR90 | Gro-Seq minus E2 |
| GRO-seq | GSE99544 | GSM3555218 | IMR90 | Groseq-plus E2 (30min) + 2,5-HD |
| GRO-seq | GSE99544 | GSM3555219 | IMR90 | Groseq-plus E2 (14hrs) + E2 1hr + 2,5-HD |
| GRO-seq | GSE125607 | GSM3578141 | MCF7 | shCtrl Veh GROseq |
| GRO-seq | GSE125607 | GSM3578142 | MCF7 | shCtrl E2 GROseq |
| GRO-seq | GSE125607 | GSM3578143 | MCF7 | shTEAD4 Veh GROseq |
| GRO-seq | GSE125607 | GSM3578144 | MCF7 | shTEAD4 E2 GROseq |
| GRO-seq | GSE125607 | GSM3578145 | MCF7 | shYAP1 Veh GROseq |
| GRO-seq | GSE125607 | GSM3578146 | MCF7 | shYAP1 E2 GROseq |
| GRO-seq | GSE139004 | GSM4126032 | MCF7 | MCF7.rep1 (GRO-seq) |
| GRO-seq | GSE139004 | GSM4126033 | MCF7 | MCF7.rep2 (GRO-seq) |
| GRO-seq | GSE139004 | GSM4126034 | MDA-MB-231 | MDA.MB.231.rep1 (GRO-seq) |
| GRO-seq | GSE139004 | GSM4126035 | MDA-MB-231 | MDA.MB.231.rep2 (GRO-seq) |
| GRO-seq | GSE115782 | GSM4162860 | NPC | NPC1-ExpA GRO-seq |
| GRO-seq | GSE115782 | GSM4162861 | NPC | NPC1-ExpB GRO-seq |
| GRO-seq | GSE129702 | GSM4272682 | A549 | GRO-seq-vehicle-treated A549cells |
| GRO-seq | GSE129702 | GSM4272683 | A549 | GRO-seq-cisplatin-treated A549cells |
| GRO-seq | GSE74142 | GSM5065646 | MCF7 | Luc KD_GRO-seq_E2 rep1 |
| GRO-seq | GSE74142 | GSM5065647 | MCF7 | Luc KD_GRO-seq_E2 rep2 |
| GRO-seq | GSE74142 | GSM5065648 | MCF7 | PARP-1 KD_GRO-seq_E2 rep1 |
| GRO-seq | GSE74142 | GSM5065649 | MCF7 | PARP-1 KD_GRO-seq_E2 rep2 |
| GRO-seq | GSE27463 | GSM678535 | MCF7 | GRO-seq_Vehicle_rep1 |
| GRO-seq | GSE27463 | GSM678536 | MCF7 | GRO-seq_Vehicle_rep2 |
| GRO-seq | GSE27463 | GSM678537 | MCF7 | GRO-seq_E2_10m_rep1 |
| GRO-seq | GSE27463 | GSM678538 | MCF7 | GRO-seq_E2_10m_rep2 |
| GRO-seq | GSE27463 | GSM678539 | MCF7 | GRO-seq_E2_40m_rep1 |
| GRO-seq | GSE27463 | GSM678540 | MCF7 | GRO-seq_E2_40m_rep2 |
| GRO-seq | GSE27463 | GSM678541 | MCF7 | GRO-seq_E2_160m_rep1 |
| GRO-seq | GSE27463 | GSM678542 | MCF7 | GRO-seq_E2_160m_rep2 |
| GRO-seq | GSE35268 | GSM874647 | A549 | GRO-Seq 0h |
| GRO-seq | GSE35268 | GSM874648 | A549 | GRO-Seq 12h WT |
| GRO-seq | GSE35268 | GSM874649 | A549 | GRO-Seq 12h delta-PAF |
| GRO-seq | GSE39878 | GSM980644 | GM12004 | GM12004_GROseq |
| GRO-seq | GSE39878 | GSM980645 | GM12750 | GM12750_GROseq |
| H3K36me3 | GSE16256 | GSM1013141 | NPC | Reference Epigenome: ChIP-Seq Analysis of H3K36me3 in Neural Progenitor Cells;<br>renlab.H3K36me3.NPC.03.01 |

| | | | | |
|---|---|---|---|---|
| H3K36me3 | GSE16256 | GSM1013142 | Thymus | Reference Epigenome: ChIP-Seq Analysis of H3K36me3 in Human Thymus Tissue; renlab.H3K36me3.STL001TH.01.01 |
| H3K36me3 | GSE16256 | GSM1013143 | Ovary | Reference Epigenome: ChIP-Seq Analysis of H3K36me3 in Human Ovary Tissue; renlab.H3K36me3.STL002OV.01.01 |
| H3K36me3 | GSE16256 | GSM1013144 | Right atrium | Reference Epigenome: ChIP-Seq Analysis of H3K36me3 in Human Right Atrium Tissue; renlab.H3K36me3.STL003RA.01.01 |
| H3K36me3 | GSE16256 | GSM1013145 | Right ventricle | Reference Epigenome: ChIP-Seq Analysis of H3K36me3 in Human Right Ventricle Tissue; renlab.H3K36me3.STL003RV.01.01 |
| H3K36me3 | GSE18927 | GSM1027293 | CD19 | Histone H3K36me3 ChIP-Seq of CD19 Primary Cells; Histone.DS22586 |
| H3K36me3 | GSE18927 | GSM1027294 | CD56 | Histone H3K36me3 ChIP-Seq of CD56 Primary Cells; Histone.DS22595 |
| H3K36me3 | GSE18927 | GSM1027295 | Fetal thymus | Histone H3K36me3 ChIP-Seq of Fetal Thymus; Histone.DS22603 |
| H3K36me3 | GSE43070 | GSM1055820 | IMR90 | ChIP-Seq H3K36me3 IMR90 |
| H3K36me3 | GSE43070 | GSM1055821 | IMR90 | ChIP-Seq H3K36me3 IMR90+TNF-Œ± |
| H3K36me3 | GSE18927 | GSM1058771 | Fetal intestine, Large | Histone H3K36me3 ChIP-Seq of Fetal Intestine, Large; Histone.DS22554 |
| H3K36me3 | GSE18927 | GSM1058772 | Fetal intestine, Small | Histone H3K36me3 ChIP-Seq of Fetal Intestine, Small; Histone.DS22557 |
| H3K36me3 | GSE18927 | GSM1058773 | Fetal muscle, Leg | Histone H3K36me3 ChIP-Seq of Fetal Muscle, Leg; Histone.DS22560 |
| H3K36me3 | GSE18927 | GSM1058774 | CD3 | Histone H3K36me3 ChIP-Seq of CD3 Primary Cells; Histone.DS22563 |
| H3K36me3 | GSE16256 | GSM1059437 | Lung | Reference Epigenome: ChIP-Seq Analysis of H3K36me3 in Human Lung Tissue; renlab.H3K36me3.STL001LG.01.01 |
| H3K36me3 | GSE16256 | GSM1059438 | Right ventricle | Reference Epigenome: ChIP-Seq Analysis of H3K36me3 in Human Right Ventricle Tissue; renlab.H3K36me3.STL001RV.01.01 |
| H3K36me3 | GSE16256 | GSM1059439 | Adrenal gland | Reference Epigenome: ChIP-Seq Analysis of H3K36me3 in Human Adrenal Gland Tissue; renlab.H3K36me3.STL002AD.01.01 |
| H3K36me3 | GSE16256 | GSM1059440 | Aorta | Reference Epigenome: ChIP-Seq Analysis of H3K36me3 in Human Aorta Tissue; renlab.H3K36me3.STL002AO.01.01 |
| H3K36me3 | GSE16256 | GSM1059441 | Bladder | Reference Epigenome: ChIP-Seq Analysis of H3K36me3 in Human Bladder Tissue; renlab.H3K36me3.STL001BL.01.01 |
| H3K36me3 | GSE16256 | GSM1059442 | Liver | Reference Epigenome: ChIP-Seq Analysis of H3K36me3 in Human Liver Tissue; renlab.H3K36me3.STL011LI.01.01 |
| H3K36me3 | GSE18927 | GSM1102788 | CD14 | Histone H3K36me3 ChIP-Seq of CD14 Primary Cells; Histone.DS22406 |
| H3K36me3 | GSE18927 | GSM1102789 | Fetal stomach | Histone H3K36me3 ChIP-Seq of Fetal Stomach; Histone.DS22600 |
| H3K36me3 | GSE18927 | GSM1102790 | CD4 | Histone H3K36me3 ChIP-Seq of CD4 Primary Cells; Histone.DS22906 |
| H3K36me3 | GSE18927 | GSM1102791 | CD8 | Histone H3K36me3 ChIP-Seq of CD8 Primary Cells; Histone.DS22910 |
| H3K36me3 | GSE18927 | GSM1102792 | Fetal placenta | Histone H3K36me3 ChIP-Seq of Fetal Placenta; Histone.DS23030 |
| H3K36me3 | GSE17312 | GSM1112805 | CD184 differentiated from HUES64 | ChIP-Seq analysis of H3K36me3 in human HUES64 derived CD184+ cells; DNA_Lib 842 |
| H3K36me3 | GSE16256 | GSM1120346 | Esophagus | Reference Epigenome: ChIP-Seq Analysis of H3K36me3 in Human Esophagus Tissue; renlab.H3K36me3.STL002EG.01.01 |
| H3K36me3 | GSE16256 | GSM1120347 | Small intestine | Reference Epigenome: ChIP-Seq Analysis of H3K36me3 in Human Small Intestine Tissue; renlab.H3K36me3.STL002SB.01.01 |
| H3K36me3 | GSE16256 | GSM1120348 | Spleen | Reference Epigenome: ChIP-Seq Analysis of H3K36me3 in Human Spleen Tissue; renlab.H3K36me3.STL002SX.01.01 |
| H3K36me3 | GSE18927 | GSM1125769 | Fetal intestine, Large | Histone H3K36me3 ChIP-Seq of Fetal Intestine, Large; Histone.DS23307 |
| H3K36me3 | GSE16368 | GSM1127068 | Breast fibroblast | H3K36me3 ChIP-Seq analysis of breast fibroblast from RM071 (A21644) |
| H3K36me3 | GSE16368 | GSM1127069 | Pancreas islet | H3K36me3 ChIP-Seq analysis of pancreas islet from ZGI 213 (A22473) |
| H3K36me3 | GSE16368 | GSM1127070 | UCSF-4 hESC | H3K36me3 ChIP-Seq analysis of UCSF-4 embryonic stem cell line (A19300) |
| H3K36me3 | GSE16368 | GSM1127075 | Eminence neural stem and progenitor cells | H3K36me3 ChIP-Seq analysis of ganglionic eminence neural stem and progenitor cells from HuFNSC04 (A19307) |
| H3K36me3 | GSE16368 | GSM1127131 | Peripheral mononuclear cell | H3K36me3 ChIP-Seq analysis of peripheral mononuclear cell from TC015 (A21630) |
| H3K36me3 | GSE16368 | GSM1127141 | Placenta (smooth chorionic membrane) | H3K36me3 ChIP-Seq analysis of placenta (smooth chorionic membrane) tissue from CTL02 (A21714) |
| H3K36me3 | GSE16368 | GSM1127144 | Placenta (amniotic membrane) | H3K36me3 ChIP-Seq analysis of placenta (amniotic membrane) tissue from CTL02 (A21721) |
| H3K36me3 | GSE16256 | GSM1127180 | Gastric | Reference Epigenome: ChIP-Seq Analysis of H3K36me3 in Human Gastric Tissue; renlab.H3K36me3.STL001GA.01.01 |
| H3K36me3 | GSE16256 | GSM1127181 | Psoas muscle | Reference Epigenome: ChIP-Seq Analysis of H3K36me3 in Human Psoas Muscle Tissue; renlab.H3K36me3.STL001PO.01.01 |
| H3K36me3 | GSE16256 | GSM1127182 | Pancreas | Reference Epigenome: ChIP-Seq Analysis of H3K36me3 in Human Pancreas Tissue; renlab.H3K36me3.STL002PA.01.01 |
| H3K36me3 | GSE18927 | GSM1160195 | Fetal muscle, Trunk | Histone H3K36me3 ChIP-Seq of Fetal Muscle, Trunk; Histone.DS23037 |
| H3K36me3 | GSE18927 | GSM1160196 | Fetal adrenal Gland | Histone H3K36me3 ChIP-Seq of Fetal Adrenal Gland; Histone.DS23068 |
| H3K36me3 | GSE18927 | GSM1220566 | Fetal spinal cord | Histone H3K36me3 ChIP-Seq of Fetal Spinal Cord; Histone.DS23075 |
| H3K36me3 | GSE50893 | GSM1233876 | GM10847 | GM10847_H3K36me3_1 |
| H3K36me3 | GSE50893 | GSM1233877 | GM10847 | GM10847_H3K36me3_2 |
| H3K36me3 | GSE50893 | GSM1233900 | GM12878 | GM12878_H3K36me3_1 |
| H3K36me3 | GSE50893 | GSM1233901 | GM12878 | GM12878_H3K36me3_2 |
| H3K36me3 | GSE50893 | GSM1233921 | GM12890 | GM12890_H3K36me3_1.2 |
| H3K36me3 | GSE50893 | GSM1233922 | GM12890 | GM12890_H3K36me3_1 |
| H3K36me3 | GSE50893 | GSM1233923 | GM12890 | GM12890_H3K36me3_2 |
| H3K36me3 | GSE50893 | GSM1233942 | GM12891 | GM12891_H3K36me3_1 |
| H3K36me3 | GSE50893 | GSM1233943 | GM12891 | GM12891_H3K36me3_2 |
| H3K36me3 | GSE50893 | GSM1233964 | GM12892 | GM12892_H3K36me3_1 |
| H3K36me3 | GSE50893 | GSM1233965 | GM12892 | GM12892_H3K36me3_2 |
| H3K36me3 | GSE50893 | GSM1233984 | GM18486 | GM18486_H3K36me3_1 |
| H3K36me3 | GSE50893 | GSM1233985 | GM18486 | GM18486_H3K36me3_2 |
| H3K36me3 | GSE50893 | GSM1233999 | GM18505 | GM18505_H3K36me3_1 |
| H3K36me3 | GSE50893 | GSM1234000 | GM18505 | GM18505_H3K36me3_2 |
| H3K36me3 | GSE50893 | GSM1234016 | GM18526 | GM18526_H3K36me3_1 |
| H3K36me3 | GSE50893 | GSM1234017 | GM18526 | GM18526_H3K36me3_2 |
| H3K36me3 | GSE50893 | GSM1234033 | GM18951 | GM18951_H3K36me3_1 |

| | | | | |
|---|---|---|---|---|
| H3K36me3 | GSE50893 | GSM1234034 | GM18951 | GM18951_H3K36me3_2 |
| H3K36me3 | GSE50893 | GSM1234050 | GM19099 | GM19099_H3K36me3_1 |
| H3K36me3 | GSE50893 | GSM1234051 | GM19099 | GM19099_H3K36me3_2 |
| H3K36me3 | GSE50893 | GSM1234067 | GM19193 | GM19193_H3K36me3_1 |
| H3K36me3 | GSE50893 | GSM1234068 | GM19193 | GM19193_H3K36me3_2 |
| H3K36me3 | GSE50893 | GSM1234086 | GM19238 | GM19238_H3K36me3_1 |
| H3K36me3 | GSE50893 | GSM1234087 | GM19238 | GM19238_H3K36me3_2 |
| H3K36me3 | GSE50893 | GSM1234108 | GM19239 | GM19239_H3K36me3_1 |
| H3K36me3 | GSE50893 | GSM1234109 | GM19239 | GM19239_H3K36me3_2 |
| H3K36me3 | GSE50893 | GSM1234130 | GM19240 | GM19240_H3K36me3_1 |
| H3K36me3 | GSE50893 | GSM1234131 | GM19240 | GM19240_H3K36me3_2 |
| H3K36me3 | GSE50893 | GSM1234151 | GM2255 | GM2255_H3K36me3_1 |
| H3K36me3 | GSE50893 | GSM1234152 | GM2255 | GM2255_H3K36me3_2 |
| H3K36me3 | GSE50893 | GSM1234169 | GM2588 | GM2588_H3K36me3_1 |
| H3K36me3 | GSE50893 | GSM1234170 | GM2588 | GM2588_H3K36me3_2 |
| H3K36me3 | GSE50893 | GSM1234187 | GM2610 | GM2610_H3K36me3_1 |
| H3K36me3 | GSE50893 | GSM1234188 | GM2610 | GM2610_H3K36me3_2 |
| H3K36me3 | GSE50893 | GSM1234205 | GM2630 | GM2630_H3K36me3_1 |
| H3K36me3 | GSE50893 | GSM1234206 | GM2630 | GM2630_H3K36me3_2 |
| H3K36me3 | GSE50893 | GSM1234227 | MS1 | SNYDER_H3K36me3_1 |
| H3K36me3 | GSE50893 | GSM1234228 | MS1 | SNYDER_H3K36me3_2.2 |
| H3K36me3 | GSE50893 | GSM1234229 | MS1 | SNYDER_H3K36me3_2 |
| H3K36me3 | GSE16256 | GSM1282364 | Right Atrium | Reference Epigenome: ChIP-Seq Analysis of H3K36me3 in Human Right Atrium Tissue; renlab.H3K36me3.STL003RA.02.01 |
| H3K36me3 | GSE54379 | GSM1314124 | KOPT-K1 | H3K36me3 ChIP-seq in KOPT-K1 |
| H3K36me3 | GSE54379 | GSM1314131 | KOPT-K1 | H3K36me3 ChIP-seq in KOPT-K1 persisters |
| H3K36me3 | GSE54379 | GSM1314138 | DND-41 | H3K36me3 ChIP-seq in DND-41 |
| H3K36me3 | GSE54379 | GSM1314145 | DND-41 | H3K36me3 ChIP-seq in DND-41 persisters |
| H3K36me3 | GSE64175 | GSM1565789 | Monocyte-derived dendritic cells | DC_MTB_H3K36me3_rep1 (ChIP-Seq) |
| H3K36me3 | GSE64175 | GSM1565796 | Monocyte-derived dendritic cells | DC_NI_H3K36me3_rep1 (ChIP-Seq) |
| H3K36me3 | GSE64175 | GSM1565803 | Monocyte-derived dendritic cells | DC_MTB_H3K36me3_rep2 (ChIP-Seq) |
| H3K36me3 | GSE64175 | GSM1565810 | Monocyte-derived dendritic cells | DC_NI_H3K36me3_rep2 (ChIP-Seq) |
| H3K36me3 | GSE16368 | GSM1582538 | Breast Fibroblast | H3K36me3 ChIP-Seq analysis of breast fibroblast from RM070 (A21651) |
| H3K36me3 | GSE65687 | GSM1603208 | T Cells | H3K36me3_Tat_ChIPSeq |
| H3K36me3 | GSE65687 | GSM1603209 | T Cells | H3K36me3_GFP_ChIPSeq |
| H3K36me3 | GSE70482 | GSM1782705 | K562 | ChIP-seq of 10 million K562 cells with H3K36ME3 antibody - replicate 1 |
| H3K36me3 | GSE71502 | GSM1835855 | Primary brain rhabdoid tumor | ChIP-seq_RT172_H3K36me3 |
| H3K36me3 | GSE71502 | GSM1835856 | Primary brain rhabdoid tumor | ChIP-seq_RT230_H3K36me3 |
| H3K36me3 | GSE71502 | GSM1835857 | Primary brain rhabdoid tumor | ChIP-seq_RT307_H3K36me3 |
| H3K36me3 | GSE71502 | GSM1835860 | Primary brain rhabdoid tumor | ChIP-seq_RT308_H3K36me3 |
| H3K36me3 | GSE71502 | GSM1835861 | Primary brain rhabdoid tumor | ChIP-seq_RT407_H3K36me3 |
| H3K36me3 | GSE53491 | GSM1922388 | IMR90 | Growing IMR90 H3K36me3 |
| H3K36me3 | GSE53491 | GSM1922390 | IMR90 | Checkpoint IMR90 H3K36me3 |
| H3K36me3 | GSE53491 | GSM1922392 | IMR90 | Ras IMR90 H3K36me3 |
| H3K36me3 | GSE53491 | GSM1922394 | IMR90 | Apo IMR90 H3K36 me3 |
| H3K36me3 | GSE75234 | GSM1946467 | TC28a2 | cell line WT k36me3 ChIP-Seq repeat 1 |
| H3K36me3 | GSE75234 | GSM1946468 | TC28a2 | cell line WT k36me3 ChIP-Seq repeat 2 |
| H3K36me3 | GSE75234 | GSM1946469 | TC28a2 | cell line K36M #1 k36me3 ChIP-Seq repeat 1 |
| H3K36me3 | GSE75234 | GSM1946470 | TC28a2 | cell line K36M #1 k36me3 ChIP-Seq repeat 2 |
| H3K36me3 | GSE75234 | GSM1946471 | TC28a2 | cell line K36M #2 k36me3 ChIP-Seq repeat 1 |
| H3K36me3 | GSE75234 | GSM1946472 | TC28a2 | cell line K36M #2 k36me3 ChIP-Seq repeat 2 |
| H3K36me3 | GSE75234 | GSM1946480 | Chondroblastoma | tissue WT k36me3 ChIP-Seq WT repeat 1 |
| H3K36me3 | GSE75234 | GSM1946481 | Chondroblastoma | tissue WT k36me3 ChIP-Seq WT repeat 2 |
| H3K36me3 | GSE75234 | GSM1946482 | Chondroblastoma | tissue K36M #1 k36me3 ChIP-Seq |
| H3K36me3 | GSE75234 | GSM1946483 | Chondroblastoma | tissue K36M #2 k36me3 ChIP-Seq |
| H3K36me3 | GSE65838 | GSM2228904 | Epidermal stem cells from foreskin | H3K36me3 Chip-Seq EpiSC |
| H3K36me3 | GSE65838 | GSM2228905 | Epidermal stem cells from foreskin | H3K36me3 Chip-Seq KC |
| H3K36me3 | GSE75270 | GSM2279164 | Embryonic kidney | NC_Rep2_H3K36me3 |
| H3K36me3 | GSE75270 | GSM2279165 | Embryonic kidney | siSPOP_Rep2_H3K36me3 |
| H3K36me3 | GSE75270 | GSM2279166 | Embryonic kidney | siSETD2_Rep2_H3K36me3 |
| H3K36me3 | GSE75270 | GSM2279167 | Embryonic kidney | siDouble_Rep2_H3K36me3 |
| H3K36me3 | GSE86664 | GSM2308424 | Panc1 | ChIP-seq from Panc1 (ENCLB555ABN) |
| H3K36me3 | GSE86664 | GSM2308425 | Panc1 | ChIP-seq from Panc1 (ENCLB555ABM) |
| H3K36me3 | GSE86668 | GSM2308432 | HCT116 | ChIP-seq from HCT116 (ENCLB555ABU) |
| H3K36me3 | GSE86668 | GSM2308433 | HCT117 | ChIP-seq from HCT116 (ENCLB555ABV) |
| H3K36me3 | GSE86673 | GSM2308442 | SK-N-SH | ChIP-seq from SK-N-SH (ENCLB555ACT) |
| H3K36me3 | GSE86673 | GSM2308443 | SK-N-SH | ChIP-seq from SK-N-SH (ENCLB555ACS) |
| H3K36me3 | GSE86692 | GSM2308483 | OCI-LY7 | ChIP-seq from OCI-LY7 (ENCLB769IJW) |
| H3K36me3 | GSE86692 | GSM2308484 | OCI-LY7 | ChIP-seq from OCI-LY7 (ENCLB942TTE) |
| H3K36me3 | GSE86697 | GSM2308493 | SUDHL6 | ChIP-seq from SUDHL6 (ENCLB190DEU) |
| H3K36me3 | GSE86697 | GSM2308494 | SUDHL6 | ChIP-seq from SUDHL6 (ENCLB035XAR) |
| H3K36me3 | GSE86709 | GSM2308516 | OCI-LY1 | ChIP-seq from OCI-LY1 (ENCLB380HWY) |
| H3K36me3 | GSE86709 | GSM2308517 | OCI-LY1 | ChIP-seq from OCI-LY1 (ENCLB371EZY) |

| | | | | |
|---|---|---|---|---|
| H3K36me3 | GSE86730 | GSM2308562 | OCI-LY3 | ChIP-seq from OCI-LY3 (ENCLB770PIT) |
| H3K36me3 | GSE86730 | GSM2308563 | OCI-LY3 | ChIP-seq from OCI-LY3 (ENCLB542IVG) |
| H3K36me3 | GSE86754 | GSM2308610 | DOHH2 | ChIP-seq from DOHH2 (ENCLB541DJK) |
| H3K36me3 | GSE86754 | GSM2308611 | DOHH2 | ChIP-seq from DOHH2 (ENCLB593ZGZ) |
| H3K36me3 | GSE86763 | GSM2308628 | Karpas-422 | ChIP-seq from Karpas-422 (ENCLB235TND) |
| H3K36me3 | GSE86763 | GSM2308629 | Karpas-422 | ChIP-seq from Karpas-422 (ENCLB045WMI) |
| H3K36me3 | GSE86769 | GSM2308641 | Fibroblast of arm | ChIP-seq from fibroblast of arm (ENCLB974HCB) |
| H3K36me3 | GSE86769 | GSM2308642 | Fibroblast of arm | ChIP-seq from fibroblast of arm (ENCLB839TOH) |
| H3K36me3 | GSE89976 | GSM2394707 | Human iPSCs | 201B7_H3K36me3 |
| H3K36me3 | GSE89976 | GSM2394711 | Human dermal fibroblast | TIG120_H3K36me3 |
| H3K36me3 | GSE93030 | GSM2442789 | HUVEC | H3K36me3 |
| H3K36me3 | GSE94726 | GSM2481429 | Calu3 | Calu3 H3K36me3 ChIP-seq |
| H3K36me3 | GSE94726 | GSM2481430 | HBE | HBE H3K36me3 ChIP-seq |
| H3K36me3 | GSE94804 | GSM2483406 | MCF7 | MCF7_H3K36me3_Rep1_ChIPSeq |
| H3K36me3 | GSE94804 | GSM2483407 | MCF7 | MCF7_H3K36me3_Rep2_ChIPSeq |
| H3K36me3 | GSE95891 | GSM2527401 | Hepatocyte | ChIP-seq from hepatocyte (ENCLB780JMR) |
| H3K36me3 | GSE95891 | GSM2527402 | Hepatocyte | ChIP-seq from hepatocyte (ENCLB161XYT) |
| H3K36me3 | GSE95914 | GSM2527452 | HCT116 | ChIP-seq from HCT116 (ENCLB571FUG) |
| H3K36me3 | GSE95914 | GSM2527453 | HCT116 | ChIP-seq from HCT116 (ENCLB901TMI) |
| H3K36me3 | GSE95923 | GSM2527469 | Gastrocnemius medialis | ChIP-seq from gastrocnemius medialis (ENCLB019XYQ) |
| H3K36me3 | GSE95923 | GSM2527470 | Gastrocnemius medialis | ChIP-seq from gastrocnemius medialis (ENCLB218LEF) |
| H3K36me3 | GSE95931 | GSM2527488 | MM.1S | ChIP-seq from MM.1S (ENCLB510QDT) |
| H3K36me3 | GSE95931 | GSM2527489 | MM.1S | ChIP-seq from MM.1S (ENCLB790RMK) |
| H3K36me3 | GSE95941 | GSM2527507 | Radial glial cell | ChIP-seq from radial glial cell (ENCLB460PBY) |
| H3K36me3 | GSE95941 | GSM2527508 | Radial glial cell | ChIP-seq from radial glial cell (ENCLB601QJE) |
| H3K36me3 | GSE95989 | GSM2527612 | Thyroid gland | ChIP-seq from thyroid gland (ENCLB734PTL) |
| H3K36me3 | GSE95989 | GSM2527613 | Thyroid gland | ChIP-seq from thyroid gland (ENCLB661MLR) |
| H3K36me3 | GSE95994 | GSM2527621 | Human iPSCs | ChIP-seq from induced pluripotent stem cell (ENCLB755YHD) |
| H3K36me3 | GSE95994 | GSM2527622 | Human iPSCs | ChIP-seq from induced pluripotent stem cell (ENCLB757FNZ) |
| H3K36me3 | GSE96142 | GSM2533965 | Bipolar spindle neuron | ChIP-seq from bipolar spindle neuron (ENCLB784BNS) |
| H3K36me3 | GSE96142 | GSM2533966 | Bipolar spindle neuron | ChIP-seq from bipolar spindle neuron (ENCLB393LQH) |
| H3K36me3 | GSE96144 | GSM2533969 | Gastrocnemius medialis | ChIP-seq from gastrocnemius medialis (ENCLB523JHM) |
| H3K36me3 | GSE96144 | GSM2533970 | Gastrocnemius medialis | ChIP-seq from gastrocnemius medialis (ENCLB177HSL) |
| H3K36me3 | GSE96144 | GSM2533971 | Gastrocnemius medialis | ChIP-seq from gastrocnemius medialis (ENCLB949DXQ) |
| H3K36me3 | GSE96144 | GSM2533972 | Gastrocnemius medialis | ChIP-seq from gastrocnemius medialis (ENCLB769OFX) |
| H3K36me3 | GSE96155 | GSM2533992 | PC-9 | ChIP-seq from PC-9 (ENCLB666EWZ) |
| H3K36me3 | GSE96155 | GSM2533993 | PC-9 | ChIP-seq from PC-9 (ENCLB830JFM) |
| H3K36me3 | GSE96165 | GSM2534013 | B cell | ChIP-seq from B cell (ENCLB480MDY) |
| H3K36me3 | GSE96165 | GSM2534014 | B cell | ChIP-seq from B cell (ENCLB896GWS) |
| H3K36me3 | GSE96198 | GSM2534077 | Heart left ventricle | ChIP-seq from heart left ventricle (ENCLB738MIE) |
| H3K36me3 | GSE96198 | GSM2534078 | Heart left ventricle | ChIP-seq from heart left ventricle (ENCLB574PHY) |
| H3K36me3 | GSE96217 | GSM2534116 | Body of pancreas | ChIP-seq from body of pancreas (ENCLB585JNL) |
| H3K36me3 | GSE96217 | GSM2534117 | Body of pancreas | ChIP-seq from body of pancreas (ENCLB614HZS) |
| H3K36me3 | GSE96236 | GSM2534154 | NCI-H929 | ChIP-seq from NCI-H929 (ENCLB727HCL) |
| H3K36me3 | GSE96236 | GSM2534155 | NCI-H929 | ChIP-seq from NCI-H929 (ENCLB427UUV) |
| H3K36me3 | GSE96247 | GSM2534176 | Neural progenitor | ChIP-seq from neural progenitor cell (ENCLB022NIZ) |
| H3K36me3 | GSE96247 | GSM2534177 | Neural progenitor | ChIP-seq from neural progenitor cell (ENCLB890WSL) |
| H3K36me3 | GSE96251 | GSM2534184 | A673 | ChIP-seq from A673 (ENCLB332YQI) |
| H3K36me3 | GSE96251 | GSM2534185 | A673 | ChIP-seq from A673 (ENCLB244JDS) |
| H3K36me3 | GSE96288 | GSM2534256 | Smooth muscle cell | ChIP-seq from smooth muscle cell (ENCLB046MKU) |
| H3K36me3 | GSE96288 | GSM2534257 | Smooth muscle cell | ChIP-seq from smooth muscle cell (ENCLB140OFC) |
| H3K36me3 | GSE96312 | GSM2534306 | Neuroepithelial stem cell | ChIP-seq from neuroepithelial stem cell (ENCLB678TOF) |
| H3K36me3 | GSE96312 | GSM2534307 | Neuroepithelial stem cell | ChIP-seq from neuroepithelial stem cell (ENCLB717TKR) |
| H3K36me3 | GSE96315 | GSM2534312 | Body of pancreas | ChIP-seq from body of pancreas (ENCLB110YUR) |
| H3K36me3 | GSE96315 | GSM2534313 | Body of pancreas | ChIP-seq from body of pancreas (ENCLB405JXR) |
| H3K36me3 | GSE96315 | GSM2534314 | Body of pancreas | ChIP-seq from body of pancreas (ENCLB267NCW) |
| H3K36me3 | GSE96331 | GSM2534348 | Neural cell | ChIP-seq from neural cell (ENCLB760LUD) |
| H3K36me3 | GSE96331 | GSM2534349 | Neural cell | ChIP-seq from neural cell (ENCLB099SCI) |
| H3K36me3 | GSE96406 | GSM2534499 | KMS-11 | ChIP-seq from KMS-11 (ENCLB241KFU) |
| H3K36me3 | GSE96406 | GSM2534500 | KMS-11 | ChIP-seq from KMS-11 (ENCLB351BJB) |
| H3K36me3 | GSE96410 | GSM2534508 | Loucy | ChIP-seq from Loucy (ENCLB650FNM) |
| H3K36me3 | GSE96410 | GSM2534509 | Loucy | ChIP-seq from Loucy (ENCLB628LUQ) |
| H3K36me3 | GSE96418 | GSM2534524 | PC-3 | ChIP-seq from PC-3 (ENCLB802IUL) |
| H3K36me3 | GSE96418 | GSM2534525 | PC-3 | ChIP-seq from PC-3 (ENCLB833SNG) |
| H3K36me3 | GSE96426 | GSM2534539 | Adrenal gland | ChIP-seq from adrenal gland (ENCLB177PZY) |
| H3K36me3 | GSE96426 | GSM2534540 | Adrenal gland | ChIP-seq from adrenal gland (ENCLB029CKM) |
| H3K36me3 | GSE96434 | GSM2534555 | H9-hESC | ChIP-seq from H9 (ENCLB006TOQ) |
| H3K36me3 | GSE96434 | GSM2534556 | H9-hESC | ChIP-seq from H9 (ENCLB540MMT) |
| H3K36me3 | GSE96444 | GSM2534580 | KOPT-K1 | ChIP-seq from KOPT-K1 (ENCLB541VFU) |
| H3K36me3 | GSE96444 | GSM2534581 | KOPT-K1 | ChIP-seq from KOPT-K1 (ENCLB856ENV) |
| H3K36me3 | GSE96454 | GSM2534596 | ACC112 | ChIP-seq from ACC112 (ENCLB667AMS) |
| H3K36me3 | GSE96454 | GSM2534597 | ACC112 | ChIP-seq from ACC112 (ENCLB190ALY) |
| H3K36me3 | GSE96503 | GSM2534703 | SK-N-SH | ChIP-seq from SK-N-SH (ENCLB471YFP) |
| H3K36me3 | GSE96503 | GSM2534704 | SK-N-SH | ChIP-seq from SK-N-SH (ENCLB691SDO) |
| H3K36me3 | GSE96503 | GSM2534705 | SK-N-SH | ChIP-seq from SK-N-SH (ENCLB800FFG) |
| H3K36me3 | GSE96507 | GSM2534712 | Thoracic aorta | ChIP-seq from thoracic aorta (ENCLB323KIY) |
| H3K36me3 | GSE96507 | GSM2534713 | Thoracic aorta | ChIP-seq from thoracic aorta (ENCLB819ZQQ) |
| H3K36me3 | GSE96513 | GSM2534724 | Cardiac muscle cell | ChIP-seq from cardiac muscle cell (ENCLB481SFE) |
| H3K36me3 | GSE96513 | GSM2534725 | Cardiac muscle cell | ChIP-seq from cardiac muscle cell (ENCLB862KDH) |

| | | | | |
|---|---|---|---|---|
| H3K36me3 | GSE96513 | GSM2534726 | Cardiac muscle cell | ChIP-seq from cardiac muscle cell (ENCLB376NHI) |
| H3K36me3 | GSE86697 | GSM2550029 | SUDHL6 | ChIP-seq from SUDHL6 (ENCLB149NCP) |
| H3K36me3 | GSE97827 | GSM2579051 | HEK293 | ChIP-seq H3K36me3 WT pol II Rep1 |
| H3K36me3 | GSE97827 | GSM2579052 | HEK293 | ChIP-seq H3K36me3 WT pol II Rep2 |
| H3K36me3 | GSE97827 | GSM2579053 | HEK293 | ChIP-seq H3K36me3 R749H pol II Rep1 |
| H3K36me3 | GSE97827 | GSM2579054 | HEK293 | ChIP-seq H3K36me3 R749H pol II Rep2 |
| H3K36me3 | GSE98714 | GSM2610542 | PrEC | PrEC H3K36me3 ChIP-seq |
| H3K36me3 | GSE98714 | GSM2610547 | LNCaP | LNCaP H3K36me3 ChIP-seq |
| H3K36me3 | GSE100040 | GSM2670997 | HCT116 | Histone H3K36me3 ChIP-seq WT Rep1 |
| H3K36me3 | GSE100040 | GSM2670998 | HCT116 | Histone H3K36me3 ChIP-seq Cdk7as Rep1 |
| H3K36me3 | GSE94804 | GSM2677938 | MCF7 | MCF7_H3K36me3_2K_Rep1_ChIPSeq |
| H3K36me3 | GSE94804 | GSM2677939 | MCF7 | MCF7_H3K36me3_2K_Rep2_ChIPSeq |
| H3K36me3 | GSE100997 | GSM2698552 | Gastrocnemius medialis | ChIP-seq from gastrocnemius medialis (ENCLB616PJK) |
| H3K36me3 | GSE100999 | GSM2698557 | Gastrocnemius medialis | ChIP-seq from esophagus squamous epithelium (ENCLB726TPD) |
| H3K36me3 | GSE101003 | GSM2698564 | Right lobe of liver | ChIP-seq from right lobe of liver (ENCLB679IKV) |
| H3K36me3 | GSE101011 | GSM2698588 | Peyer's patch | ChIP-seq from Peyer's patch (ENCLB894AZQ) |
| H3K36me3 | GSE101013 | GSM2698597 | Thoracic aorta | ChIP-seq from thoracic aorta (ENCLB617YGS) |
| H3K36me3 | GSE101015 | GSM2698600 | Neutrophil | ChIP-seq from neutrophil (ENCLB954RWK) |
| H3K36me3 | GSE101015 | GSM2698601 | Neutrophil | ChIP-seq from neutrophil (ENCLB889GSB) |
| H3K36me3 | GSE101026 | GSM2698624 | Adrenal gland | ChIP-seq from adrenal gland (ENCLB902CFJ) |
| H3K36me3 | GSE101032 | GSM2698632 | Tibial artery | ChIP-seq from tibial artery (ENCLB460WDH) |
| H3K36me3 | GSE101037 | GSM2698638 | Sigmoid colon | ChIP-seq from sigmoid colon (ENCLB331CFF) |
| H3K36me3 | GSE101047 | GSM2698675 | Tibial nerve | ChIP-seq from tibial nerve (ENCLB904CMO) |
| H3K36me3 | GSE101053 | GSM2698709 | Stomach | ChIP-seq from stomach (ENCLB742OEJ) |
| H3K36me3 | GSE101057 | GSM2698743 | Ascending aorta | ChIP-seq from ascending aorta (ENCLB084AUH) |
| H3K36me3 | GSE101191 | GSM2700198 | SK-N-MC | ChIP-seq from SK-N-MC (ENCLB841KOB) |
| H3K36me3 | GSE101191 | GSM2700199 | SK-N-MC | ChIP-seq from SK-N-MC (ENCLB306XER) |
| H3K36me3 | GSE101206 | GSM2700337 | Breast epithelium | ChIP-seq from breast epithelium (ENCLB259KXY) |
| H3K36me3 | GSE101227 | GSM2700497 | Spleen | ChIP-seq from spleen (ENCLB582YII) |
| H3K36me3 | GSE101264 | GSM2700591 | Ascending aorta | ChIP-seq from ascending aorta (ENCLB900MRP) |
| H3K36me3 | GSE101268 | GSM2700596 | Esophagus muscularis mucosa | ChIP-seq from esophagus muscularis mucosa (ENCLB919IPT) |
| H3K36me3 | GSE101347 | GSM2701567 | Thyroid gland | ChIP-seq from thyroid gland (ENCLB483WUJ) |
| H3K36me3 | GSE101352 | GSM2701630 | Upper lob of left lung | ChIP-seq from upper lobe of left lung (ENCLB813VIH) |
| H3K36me3 | GSE101366 | GSM2701747 | Thyroid gland | ChIP-seq from thyroid gland (ENCLB409QRH) |
| H3K36me3 | GSE101374 | GSM2701782 | Vagina | ChIP-seq from vagina (ENCLB483YHJ) |
| H3K36me3 | GSE101375 | GSM2701783 | Right atrium auricular region | ChIP-seq from right atrium auricular region (ENCLB868TGI) |
| H3K36me3 | GSE100040 | GSM2722166 | HCT116 | Histone H3K36me3 ChIP-seq WT Rep2 |
| H3K36me3 | GSE100040 | GSM2722167 | HCT116 | Histone H3K36me3 ChIP-seq Cdk7as Rep2 |
| H3K36me3 | GSE103554 | GSM2773923 | SCC25 | SCC25_1 H3K36me3 (biological replicate 1) |
| H3K36me3 | GSE103554 | GSM2773930 | SCC25 | SCC25_2 H3K36me3 (biological replicate 2) |
| H3K36me3 | GSE103554 | GSM2773937 | HGEP | HGEP_2 H3K36me3 (biological replicate 1) |
| H3K36me3 | GSE103554 | GSM2773944 | HGEP | HGEP_3 H3K36me3 (biological replicate 2) |
| H3K36me3 | GSE104481 | GSM2801177 | H23 | H23_H3K36me3_REP1 |
| H3K36me3 | GSE104481 | GSM2801188 | H23 | H23_H3K36me3_REP2 |
| H3K36me3 | GSE104334 | GSM2809623 | HCT116 | Rao-2017-CHIP015-H3K36me3-untreated |
| H3K36me3 | GSE104334 | GSM2809624 | HCT116 | Rao-2017-CHIP016-H3K36me3-treated |
| H3K36me3 | GSE107785 | GSM2879157 | Mesoderm stage cells differentiated from HES3 | H3K36me3-WT |
| H3K36me3 | GSE107785 | GSM2879158 | Mesoderm stage cells differentiated from HES3 | H3K36me3-KO |
| H3K36me3 | GSE109625 | GSM2983241 | HUVEC | H3K36me3-0hour |
| H3K36me3 | GSE109625 | GSM2983242 | HUVEC | H3K36me3-1hour |
| H3K36me3 | GSE109625 | GSM2983243 | HUVEC | H3K36me3-4hour |
| H3K36me3 | GSE109625 | GSM2983244 | HUVEC | H3K36me3-12hour |
| H3K36me3 | GSE110318 | GSM2987412 | HepG2 | HepG2 shCtrl H3K36me3-rep1 (ChIP-seq) |
| H3K36me3 | GSE110318 | GSM2987413 | HepG2 | HepG2 shSETD2 H3K36me3-rep1 (ChIP-seq) |
| H3K36me3 | GSE110318 | GSM2987416 | HepG2 | HepG2 shCtrl H3K36me3-rep2 (ChIP-seq) |
| H3K36me3 | GSE110318 | GSM2987417 | HepG2 | HepG2 shSETD2 H3K36me3-rep2 (ChIP-seq) |
| H3K36me3 | GSE110389 | GSM2990407 | HeLa | H3K36me3 ChIP-seq in G34L |
| H3K36me3 | GSE110389 | GSM2990410 | HeLa | H3K36me3 ChIP-seq in K36M |
| H3K36me3 | GSE110389 | GSM2990416 | HeLa | H3K36me3 ChIP-seq in WT |
| H3K36me3 | GSE110389 | GSM2990422 | HeLa | H3K36me3 ChIP-seq in G34W |
| H3K36me3 | GSE111000 | GSM3019941 | HepG2 | Traditional HepG2 H3K36me3 |
| H3K36me3 | GSE111482 | GSM3032098 | HSPC | H3K36me3 ChIP-seq on 4w cultured HSPC with CTR virus |
| H3K36me3 | GSE111482 | GSM3032099 | HSPC | H3K36me3 ChIP-seq on 4w cultured HSPC with MLLT3 virus |
| H3K36me3 | GSE103477 | GSM3111923 | MDM | MDM-H3K36me3-Mock-r1 |
| H3K36me3 | GSE103477 | GSM3111924 | MDM | MDM-H3K36me3-Mock-r2 |
| H3K36me3 | GSE103477 | GSM3111925 | MDM | MDM-H3K36me3-H5N1-r1 |
| H3K36me3 | GSE103477 | GSM3111926 | MDM | MDM-H3K36me3-H5N1-r2 |
| H3K36me3 | GSE103477 | GSM3111927 | MDM | MDM-H3K36me3-dNS1-r1 |
| H3K36me3 | GSE103477 | GSM3111928 | MDM | MDM-H3K36me3-dNS1-r2 |
| H3K36me3 | GSE103477 | GSM3111929 | MDM | MDM-H3K36me3-IFNb-r1 |
| H3K36me3 | GSE103477 | GSM3111930 | MDM | MDM-H3K36me3-IFNb-r2 |
| H3K36me3 | GSE110354 | GSM3227892 | HeLa S3 | H3K36me3_ChIP-seq_Parental_rep1 |
| H3K36me3 | GSE110354 | GSM3227893 | HeLa S3 | H3K36me3_ChIP-seq_Parental_rep2 |
| H3K36me3 | GSE110354 | GSM3227894 | HeLa S3 | H3K36me3_ChOR-seq_T0_rep1 |
| H3K36me3 | GSE110354 | GSM3227895 | HeLa S3 | H3K36me3_ChOR-seq_T0_rep2 |
| H3K36me3 | GSE118588 | GSM3333902 | EndoC-BH1 | EndoC_BH1_ChIP_seq_H3K36me3 |
| H3K36me3 | GSE120318 | GSM3398285 | Fetal lung | ChIP-seq from lung (ENCLB755OFI) |

| | | | | |
|---|---|---|---|---|
| H3K36me3 | GSE118629 | GSM3564261 | 22Rv1 | H3K36me3 ChIP-seq in 22Rv1 rep1 |
| H3K36me3 | GSE118629 | GSM3564262 | 22Rv1 | H3K36me3 ChIP-seq in 22Rv1 rep2 |
| H3K36me3 | GSE118629 | GSM3564267 | RWPE1 | H3K36me3 ChIP-seq in RWPE1 rep1 |
| H3K36me3 | GSE118629 | GSM3564268 | RWPE1 | H3K36me3 ChIP-seq in RWPE1 rep2 |
| H3K36me3 | GSE118629 | GSM3564273 | C42B | H3K36me3 ChIP-seq in C42B rep1 |
| H3K36me3 | GSE118629 | GSM3564274 | C42B | H3K36me3 ChIP-seq in C42B rep2 |
| H3K36me3 | GSE129031 | GSM3691944 | MSC | MSC_H3K36me3_Rep1 |
| H3K36me3 | GSE129031 | GSM3691950 | Chondrocytes derived via in vitro culture from MSC | CHON_H3K36me3_Rep1 |
| H3K36me3 | GSE129031 | GSM3691956 | MSC | MSC_H3K36me3_Rep2 |
| H3K36me3 | GSE129031 | GSM3691962 | Chondrocytes derived via in vitro culture from MSC | CHON_H3K36me3_Rep2 |
| H3K36me3 | GSE136475 | GSM4050984 | Esophagus muscularis mucosa | Histone ChIP-seq from esophagus muscularis mucosa (ENCLB847DDD) |
| H3K36me3 | GSE136475 | GSM4050985 | Esophagus muscularis mucosa | Histone ChIP-seq from esophagus muscularis mucosa (ENCLB367XLA) |
| H3K36me3 | GSE136508 | GSM4051109 | Upper lobe of left lung | Histone ChIP-seq from upper lobe of left lung (ENCLB468DDW) |
| H3K36me3 | GSE136508 | GSM4051110 | Upper lobe of left lung | Histone ChIP-seq from upper lobe of left lung (ENCLB692ITP) |
| H3K36me3 | GSE136518 | GSM4051128 | Heart left ventricle | Histone ChIP-seq from heart left ventricle (ENCLB545GKJ) |
| H3K36me3 | GSE136518 | GSM4051129 | Heart left ventricle | Histone ChIP-seq from heart left ventricle (ENCLB622JVK) |
| H3K36me3 | GSE136524 | GSM4051140 | Esophagus muscularis mucosa | Histone ChIP-seq from esophagus muscularis mucosa (ENCLB877RRK) |
| H3K36me3 | GSE136524 | GSM4051141 | Esophagus muscularis mucosa | Histone ChIP-seq from esophagus muscularis mucosa (ENCLB348QKR) |
| H3K36me3 | GSE16256 | GSM409312 | H1 hESC | Reference Epigenome: ChIP-Seq Analysis of H3K36me3 in hESC H1 (CDI-01) Cells; renlab.H3K36me3.CDI-01.01 |
| H3K36me3 | GSE139053 | GSM4128447 | Aska | Aska_shCt_H3K36me3_ChIP-Seq |
| H3K36me3 | GSE139053 | GSM4128449 | SYO1 | SYO1_shCt_H3K36me3_ChIP-Seq |
| H3K36me3 | GSE139691 | GSM4146344 | Sigmoid colon | Histone ChIP-seq from sigmoid colon (ENCLB385PBC) |
| H3K36me3 | GSE139691 | GSM4146345 | Sigmoid colon | Histone ChIP-seq from sigmoid colon (ENCLB616NMJ) |
| H3K36me3 | GSE139693 | GSM4146347 | Peyer's patch | Histone ChIP-seq from Peyer's patch (ENCLB671VIN) |
| H3K36me3 | GSE139693 | GSM4146348 | Peyer's patch | Histone ChIP-seq from Peyer's patch (ENCLB708THL) |
| H3K36me3 | GSE139695 | GSM4146351 | Esophagus squamous epithelium | Histone ChIP-seq from esophagus squamous epithelium (ENCLB803GPX) |
| H3K36me3 | GSE139695 | GSM4146352 | Esophagus squamous epithelium | Histone ChIP-seq from esophagus squamous epithelium (ENCLB246GZR) |
| H3K36me3 | GSE139709 | GSM4146387 | Tibial artery | Histone ChIP-seq from tibial artery (ENCLB461AUO) |
| H3K36me3 | GSE139709 | GSM4146388 | Tibial artery | Histone ChIP-seq from tibial artery (ENCLB747YVN) |
| H3K36me3 | GSE139731 | GSM4146446 | Tibial nerve | Histone ChIP-seq from tibial nerve (ENCLB121VDD) |
| H3K36me3 | GSE139731 | GSM4146447 | Tibial nerve | Histone ChIP-seq from tibial nerve (ENCLB961WYY) |
| H3K36me3 | GSE139737 | GSM4146457 | Upper lobe of left lung | Histone ChIP-seq from upper lobe of left lung (ENCLB934ZCF) |
| H3K36me3 | GSE139737 | GSM4146458 | Upper lobe of left lung | Histone ChIP-seq from upper lobe of left lung (ENCLB177PSJ) |
| H3K36me3 | GSE139742 | GSM4146465 | Prostate gland | Histone ChIP-seq from prostate gland (ENCLB846VZG) |
| H3K36me3 | GSE139742 | GSM4146466 | Prostate gland | Histone ChIP-seq from prostate gland (ENCLB501LTM) |
| H3K36me3 | GSE139742 | GSM4146467 | Prostate gland | Histone ChIP-seq from prostate gland (ENCLB182NZE) |
| H3K36me3 | GSE139742 | GSM4146468 | Prostate gland | Histone ChIP-seq from prostate gland (ENCLB911TVH) |
| H3K36me3 | GSE139747 | GSM4146478 | Breast epithelium | Histone ChIP-seq from breast epithelium (ENCLB284QRA) |
| H3K36me3 | GSE139747 | GSM4146479 | Breast epithelium | Histone ChIP-seq from breast epithelium (ENCLB777AFJ) |
| H3K36me3 | GSE139749 | GSM4146482 | Gastroesophageal sphincter | Histone ChIP-seq from gastroesophageal sphincter (ENCLB064YYG) |
| H3K36me3 | GSE139749 | GSM4146483 | Gastroesophageal sphincter | Histone ChIP-seq from gastroesophageal sphincter (ENCLB381CUO) |
| H3K36me3 | GSE139750 | GSM4146484 | Right atrium auricular region | Histone ChIP-seq from right atrium auricular region (ENCLB822STS) |
| H3K36me3 | GSE139750 | GSM4146485 | Right atrium auricular region | Histone ChIP-seq from right atrium auricular region (ENCLB170WND) |
| H3K36me3 | GSE139758 | GSM4146500 | Suprapubic skin | Histone ChIP-seq from suprapubic skin (ENCLB781HGP) |
| H3K36me3 | GSE139758 | GSM4146501 | Suprapubic skin | Histone ChIP-seq from suprapubic skin (ENCLB819BNH) |
| H3K36me3 | GSE139762 | GSM4146510 | Spleen | Histone ChIP-seq from spleen (ENCLB860THE) |
| H3K36me3 | GSE139762 | GSM4146511 | Spleen | Histone ChIP-seq from spleen (ENCLB150JHM) |
| H3K36me3 | GSE139762 | GSM4146512 | Spleen | Histone ChIP-seq from spleen (ENCLB780MOQ) |
| H3K36me3 | GSE139762 | GSM4146513 | Spleen | Histone ChIP-seq from spleen (ENCLB513FZJ) |
| H3K36me3 | GSE139802 | GSM4146597 | Stomach | Histone ChIP-seq from stomach (ENCLB818GZW) |
| H3K36me3 | GSE139802 | GSM4146598 | Stomach | Histone ChIP-seq from stomach (ENCLB627PPJ) |
| H3K36me3 | GSE142878 | GSM4247113 | Stomach | Histone ChIP-seq from stomach (ENCLB302NEB) |
| H3K36me3 | GSE142878 | GSM4247114 | Stomach | Histone ChIP-seq from stomach (ENCLB358YCU) |
| H3K36me3 | GSE142882 | GSM4247160 | Ovary | Histone ChIP-seq from ovary (ENCLB708IKO) |
| H3K36me3 | GSE142882 | GSM4247161 | Ovary | Histone ChIP-seq from ovary (ENCLB291XEE) |
| H3K36me3 | GSE142887 | GSM4247168 | Vagina | Histone ChIP-seq from vagina (ENCLB732WKH) |
| H3K36me3 | GSE142887 | GSM4247169 | Vagina | Histone ChIP-seq from vagina (ENCLB703FEC) |
| H3K36me3 | GSE142888 | GSM4247170 | Transverse colon | Histone ChIP-seq from transverse colon (ENCLB913MCD) |
| H3K36me3 | GSE142888 | GSM4247171 | Transverse colon | Histone ChIP-seq from transverse colon (ENCLB812XOG) |
| H3K36me3 | GSE142888 | GSM4247172 | Transverse colon | Histone ChIP-seq from transverse colon (ENCLB781XYA) |
| H3K36me3 | GSE142888 | GSM4247173 | Transverse colon | Histone ChIP-seq from transverse colon (ENCLB636AGV) |
| H3K36me3 | GSE142904 | GSM4247236 | Transverse colon | Histone ChIP-seq from transverse colon (ENCLB454KSS) |
| H3K36me3 | GSE142904 | GSM4247237 | Transverse colon | Histone ChIP-seq from transverse colon (ENCLB911PQY) |
| H3K36me3 | GSE142904 | GSM4247238 | Transverse colon | Histone ChIP-seq from transverse colon (ENCLB820OPP) |
| H3K36me3 | GSE142904 | GSM4247239 | Transverse colon | Histone ChIP-seq from transverse colon (ENCLB340GKS) |
| H3K36me3 | GSE142954 | GSM4247347 | Sigmoid colon | Histone ChIP-seq from sigmoid colon (ENCLB065XDU) |
| H3K36me3 | GSE142954 | GSM4247348 | Sigmoid colon | Histone ChIP-seq from sigmoid colon (ENCLB293SJZ) |
| H3K36me3 | GSE142956 | GSM4247351 | Sigmoid colon | Histone ChIP-seq from sigmoid colon (ENCLB433SBQ) |
| H3K36me3 | GSE142956 | GSM4247352 | Sigmoid colon | Histone ChIP-seq from sigmoid colon (ENCLB451VHR) |
| H3K36me3 | GSE142972 | GSM4247386 | Uterus | Histone ChIP-seq from uterus (ENCLB713FBA) |
| H3K36me3 | GSE142972 | GSM4247387 | Uterus | Histone ChIP-seq from uterus (ENCLB082YHM) |
| H3K36me3 | GSE142988 | GSM4247538 | Transverse colon | Histone ChIP-seq from transverse colon (ENCLB101KYS) |
| H3K36me3 | GSE142988 | GSM4247539 | Transverse colon | Histone ChIP-seq from transverse colon (ENCLB865RCV) |
| H3K36me3 | GSE142988 | GSM4247540 | Transverse colon | Histone ChIP-seq from transverse colon (ENCLB086LAE) |

| | | | | |
|---|---|---|---|---|
| H3K36me3 | GSE142988 | GSM4247541 | Transverse colon | Histone ChIP-seq from transverse colon (ENCLB434DYQ) |
| H3K36me3 | GSE143015 | GSM4248461 | Adrenal gland | Histone ChIP-seq from adrenal gland (ENCLB680IME) |
| H3K36me3 | GSE143015 | GSM4248462 | Adrenal gland | Histone ChIP-seq from adrenal gland (ENCLB781AZF) |
| H3K36me3 | GSE143037 | GSM4249125 | Tibial nerve | Histone ChIP-seq from tibial nerve (ENCLB389DOJ) |
| H3K36me3 | GSE143037 | GSM4249126 | Tibial nerve | Histone ChIP-seq from tibial nerve (ENCLB649WWR) |
| H3K36me3 | GSE143066 | GSM4250640 | Gastrocnemius medialis | Histone ChIP-seq from gastrocnemius medialis (ENCLB393NVY) |
| H3K36me3 | GSE143066 | GSM4250641 | Gastrocnemius medialis | Histone ChIP-seq from gastrocnemius medialis (ENCLB120PIP) |
| H3K36me3 | GSE143067 | GSM4250642 | Peyer's patch | Histone ChIP-seq from Peyer's patch (ENCLB706DPE) |
| H3K36me3 | GSE143067 | GSM4250643 | Peyer's patch | Histone ChIP-seq from Peyer's patch (ENCLB852VRU) |
| H3K36me3 | GSE143072 | GSM4250652 | Transverse colon | Histone ChIP-seq from transverse colon (ENCLB752BTM) |
| H3K36me3 | GSE143072 | GSM4250653 | Transverse colon | Histone ChIP-seq from transverse colon (ENCLB510VIZ) |
| H3K36me3 | GSE143072 | GSM4250654 | Transverse colon | Histone ChIP-seq from transverse colon (ENCLB078JPU) |
| H3K36me3 | GSE143072 | GSM4250655 | Transverse colon | Histone ChIP-seq from transverse colon (ENCLB707WDM) |
| H3K36me3 | GSE143090 | GSM4250689 | Spleen | Histone ChIP-seq from spleen (ENCLB932PMD) |
| H3K36me3 | GSE143090 | GSM4250690 | Spleen | Histone ChIP-seq from spleen (ENCLB756CFA) |
| H3K36me3 | GSE143090 | GSM4250691 | Spleen | Histone ChIP-seq from spleen (ENCLB551NYK) |
| H3K36me3 | GSE143090 | GSM4250692 | Spleen | Histone ChIP-seq from spleen (ENCLB503JXA) |
| H3K36me3 | GSE143097 | GSM4250709 | Esophagus muscularis mucosa | Histone ChIP-seq from esophagus muscularis mucosa (ENCLB330AYL) |
| H3K36me3 | GSE143110 | GSM4250736 | Tibial nerve | Histone ChIP-seq from tibial nerve (ENCLB971LWT) |
| H3K36me3 | GSE143110 | GSM4250737 | Tibial nerve | Histone ChIP-seq from tibial nerve (ENCLB553FLQ) |
| H3K36me3 | GSE143113 | GSM4250742 | Thyroid gland | Histone ChIP-seq from thyroid gland (ENCLB460FLA) |
| H3K36me3 | GSE143113 | GSM4250743 | Thyroid gland | Histone ChIP-seq from thyroid gland (ENCLB558BYX) |
| H3K36me3 | GSE143113 | GSM4250744 | Thyroid gland | Histone ChIP-seq from thyroid gland (ENCLB814UNT) |
| H3K36me3 | GSE143118 | GSM4250754 | Esophagus squamous epithelium | Histone ChIP-seq from esophagus squamous epithelium (ENCLB793FWQ) |
| H3K36me3 | GSE143118 | GSM4250755 | Esophagus squamous epithelium | Histone ChIP-seq from esophagus squamous epithelium (ENCLB051VTH) |
| H3K36me3 | GSE16368 | GSM428296 | H1 hESC | H3K36me3 ChIP-Seq analysis of H1 embryonic stem cell line (HS1032) |
| H3K36me3 | GSE17312 | GSM433176 | H1 hESC | ChIP-Seq analysis of H3K36me3 in human H1 cells, 30HCBAAXX090610-7-S |
| H3K36me3 | GSE148722 | GSM4477555 | Synovial sarcoma spheroids 1 | H3K36me3 ChIP-seq in synovial sarcoma spheroids 1 [SS1.spheres.H3K36me3] |
| H3K36me3 | GSE148722 | GSM4477565 | Synovial sarcoma spheroids 2 | H3K36me3 ChIP-seq in synovial sarcoma spheroids 2 [SS2.spheres.H3K36me3] |
| H3K36me3 | GSE148722 | GSM4477573 | Synovial sarcoma spheroids 3 | H3K36me3 ChIP-seq in synovial sarcoma spheroids 3 [SS3.spheres.H3K36me3] |
| H3K36me3 | GSE148722 | GSM4477581 | Synovial sarcoma spheroids 4 | H3K36me3 ChIP-seq in synovial sarcoma spheroids 4 [SS4.spheres.H3K36me3] |
| H3K36me3 | GSE149295 | GSM4495713 | T cell | T_cell_ChIP_H3K36me3_rep1 |
| H3K36me3 | GSE149295 | GSM4495714 | T cell | T_cell_ChIP_T_H3K36me3_rep2 |
| H3K36me3 | GSE16368 | GSM450268 | H1 hESC | H3K36me3 ChIP-Seq analysis of H1 embryonic stem cell line (HS1347) |
| H3K36me3 | GSE150195 | GSM4542952 | HeLa | ChIP-seq_H3K36me_naive |
| H3K36me3 | GSE150195 | GSM4542953 | HeLa | ChIP-seq_H3K36me3_priming |
| H3K36me3 | GSE150195 | GSM4542954 | HeLa | ChIP-seq_H3K36me3_2_days_washout |
| H3K36me3 | GSE150195 | GSM4542955 | HeLa | ChIP-seq_H3K36me3_7_days_washout |
| H3K36me3 | GSE135295 | GSM4551176 | SupT1 | Chip-seq SupT1/WT H3K36me3 rep1 |
| H3K36me3 | GSE135295 | GSM4551177 | SupT1 | Chip-seq SupT1/WT H3K36me3 rep2 |
| H3K36me3 | GSE135295 | GSM4551181 | Jurkat | Chip-seq Jurkat/WT H3K36me3 rep1 |
| H3K36me3 | GSE135295 | GSM4551182 | Jurkat | Chip-seq Jurkat/WT H3K36me3 rep2 |
| H3K36me3 | GSE135295 | GSM4551186 | Jurkat | Chip-seq Jurkat/LEDGINS H3K36me3 rep1 |
| H3K36me3 | GSE135295 | GSM4551187 | Jurkat | Chip-seq Jurkat/LEDGINS H3K36me3 rep2 |
| H3K36me3 | GSE152448 | GSM4616031 | H9 hESC | ChIP-seq_WT_H3K36me3 |
| H3K36me3 | GSE152448 | GSM4616034 | H9 hESC | ChIP-seq_UTX_KO1_H3K36me3 |
| H3K36me3 | GSE152448 | GSM4616037 | H9 hESC | ChIP-seq_UTX_KO2_H3K36me3 |
| H3K36me3 | GSE16256 | GSM466737 | H1 hESC | Reference Epigenome: ChIP-Seq Analysis of H3K36me3 in hESC H1 (hESC-01) Cells; renlab.H3K36me3.hESC-01.01 |
| H3K36me3 | GSE157308 | GSM4761256 | Hudep2 | 1440319_H3K36me3_H2-D3 |
| H3K36me3 | GSE157308 | GSM4761257 | Hudep2 | 1440320_H3K36me3_H2-D3 |
| H3K36me3 | GSE19465 | GSM486705 | CD34 | ChIP-Seq analysis of H3K36me3 in human CD34 cells, 30CAVAAXX081115-8-S |
| H3K36me3 | GSE19465 | GSM486714 | CD34 | ChIP-Seq analysis of H3K36me3 in human CD34 cells, 30EBJAAXX090206-7-S |
| H3K36me3 | GSE163315 | GSM4977047 | RPE | RPE_ChIP_H3K36me3_Rep1 |
| H3K36me3 | GSE163315 | GSM4977048 | RPE | RPE_ChIP_H3K36me3_Rep2 |
| H3K36me3 | GSE163548 | GSM4981436 | Primary FLS | 335: H3K36me3 ChIP-seq, Primary FLS, Unstimulated |
| H3K36me3 | GSE163548 | GSM4981444 | Primary FLS | 368: H3K36me3 ChIP-seq, Primary FLS, Unstimulated |
| H3K36me3 | GSE163548 | GSM4981452 | Primary FLS | 384: H3K36me3 ChIP-seq, Primary FLS, Unstimulated |
| H3K36me3 | GSE163548 | GSM4981460 | Primary FLS | 341: H3K36me3 ChIP-seq, Primary FLS, Unstimulated |
| H3K36me3 | GSE163548 | GSM4981468 | Primary FLS | 343: H3K36me3 ChIP-seq, Primary FLS, Unstimulated |
| H3K36me3 | GSE163548 | GSM4981476 | Primary FLS | 316: H3K36me3 ChIP-seq, Primary FLS, Unstimulated |
| H3K36me3 | GSE163548 | GSM4981484 | Primary FLS | 88: H3K36me3 ChIP-seq, Primary FLS, Unstimulated |
| H3K36me3 | GSE167624 | GSM5111605 | Spleen | Histone ChIP-seq from spleen (ENCLB305QYU) |
| H3K36me3 | GSE167624 | GSM5111606 | Spleen | Histone ChIP-seq from spleen (ENCLB588JXV) |
| H3K36me3 | GSE167646 | GSM5111706 | Spleen | Histone ChIP-seq from heart right ventricle (ENCLB817HXJ) |
| H3K36me3 | GSE167646 | GSM5111707 | Spleen | Histone ChIP-seq from heart right ventricle (ENCLB692ZBC) |
| H3K36me3 | GSE167667 | GSM5111815 | Spleen | Histone ChIP-seq from WERI-Rb-1 (ENCLB876FPG) |
| H3K36me3 | GSE167667 | GSM5111816 | Spleen | Histone ChIP-seq from WERI-Rb-1 (ENCLB124UCU) |
| H3K36me3 | GSE167668 | GSM5111817 | Spleen | Histone ChIP-seq from spleen (ENCLB428LLR) |
| H3K36me3 | GSE167668 | GSM5111818 | Spleen | Histone ChIP-seq from spleen (ENCLB667IGV) |
| H3K36me3 | GSE167668 | GSM5111819 | Spleen | Histone ChIP-seq from spleen (ENCLB906PJM) |
| H3K36me3 | GSE167682 | GSM5111870 | Heart left ventricle | Histone ChIP-seq from heart left ventricle (ENCLB754SZO) |
| H3K36me3 | GSE167682 | GSM5111871 | Heart left ventricle | Histone ChIP-seq from heart left ventricle (ENCLB228BFT) |
| H3K36me3 | GSE167684 | GSM5111874 | Tibial artery | Histone ChIP-seq from tibial artery (ENCLB658ZVD) |
| H3K36me3 | GSE167684 | GSM5111875 | Tibial artery | Histone ChIP-seq from tibial artery (ENCLB788YIU) |
| H3K36me3 | GSE167685 | GSM5111876 | Gastroesophageal sphincter | Histone ChIP-seq from gastroesophageal sphincter (ENCLB965OUM) |
| H3K36me3 | GSE167685 | GSM5111877 | Gastroesophageal sphincter | Histone ChIP-seq from gastroesophageal sphincter (ENCLB190UYK) |

| | | | | |
|---|---|---|---|---|
| H3K36me3 | GSE167687 | GSM5111880 | Brain microvascular endothelial primary cell | Histone ChIP-seq from brain microvascular endothelial cell (ENCLB092YUL) |
| H3K36me3 | GSE167687 | GSM5111881 | Brain microvascular endothelial primary cell | Histone ChIP-seq from brain microvascular endothelial cell (ENCLB655BNS) |
| H3K36me3 | GSE167693 | GSM5111899 | Heart left ventricle | Histone ChIP-seq from heart left ventricle (ENCLB016HUQ) |
| H3K36me3 | GSE167693 | GSM5111900 | Heart left ventricle | Histone ChIP-seq from heart left ventricle (ENCLB468UMX) |
| H3K36me3 | GSE167698 | GSM5111909 | BE2C | Histone ChIP-seq from BE2C (ENCLB602RFE) |
| H3K36me3 | GSE167698 | GSM5111910 | BE2C | Histone ChIP-seq from BE2C (ENCLB845RJF) |
| H3K36me3 | GSE167700 | GSM5111931 | RWPE2 | Histone ChIP-seq from RWPE2 (ENCLB843XFX) |
| H3K36me3 | GSE167700 | GSM5111932 | RWPE2 | Histone ChIP-seq from RWPE2 (ENCLB259TAC) |
| H3K36me3 | GSE167706 | GSM5111988 | HAP-1 | Histone ChIP-seq from HAP-1 (ENCLB997OFS) |
| H3K36me3 | GSE167706 | GSM5111989 | HAP-1 | Histone ChIP-seq from HAP-1 (ENCLB980NPH) |
| H3K36me3 | GSE167712 | GSM5112002 | MG63 | Histone ChIP-seq from MG63 (ENCLB221IFK) |
| H3K36me3 | GSE167712 | GSM5112003 | MG63 | Histone ChIP-seq from MG63 (ENCLB549VYE) |
| H3K36me3 | GSE167720 | GSM5112016 | Pancreas | Histone ChIP-seq from pancreas (ENCLB114BHE) |
| H3K36me3 | GSE167720 | GSM5112017 | Pancreas | Histone ChIP-seq from pancreas (ENCLB753CHF) |
| H3K36me3 | GSE167728 | GSM5112044 | Lower lobe of left lung | Histone ChIP-seq from lower lobe of left lung (ENCLB238TEA) |
| H3K36me3 | GSE167728 | GSM5112045 | Lower lobe of left lung | Histone ChIP-seq from lower lobe of left lung (ENCLB333IRM) |
| H3K36me3 | GSE167744 | GSM5112163 | Uterus | Histone ChIP-seq from uterus (ENCLB887JKG) |
| H3K36me3 | GSE167744 | GSM5112164 | Uterus | Histone ChIP-seq from uterus (ENCLB602OAG) |
| H3K36me3 | GSE167754 | GSM5112248 | Heart right ventricle | Histone ChIP-seq from heart right ventricle (ENCLB876QQO) |
| H3K36me3 | GSE167754 | GSM5112249 | Heart right ventricle | Histone ChIP-seq from heart right ventricle (ENCLB548TTQ) |
| H3K36me3 | GSE167769 | GSM5112396 | Left lung | Histone ChIP-seq from left lung (ENCLB627PWQ) |
| H3K36me3 | GSE167769 | GSM5112397 | Left lung | Histone ChIP-seq from left lung (ENCLB095PXQ) |
| H3K36me3 | GSE167808 | GSM5112808 | Lower lobe of left lung | Histone ChIP-seq from lower lobe of left lung (ENCLB749QXM) |
| H3K36me3 | GSE167808 | GSM5112809 | Lower lobe of left lung | Histone ChIP-seq from lower lobe of left lung (ENCLB394RWB) |
| H3K36me3 | GSE167821 | GSM5112840 | SJSA1 | Histone ChIP-seq from SJSA1 (ENCLB592LUO) |
| H3K36me3 | GSE167821 | GSM5112841 | SJSA1 | Histone ChIP-seq from SJSA1 (ENCLB961XKU) |
| H3K36me3 | GSE167840 | GSM5113886 | Spleen | Histone ChIP-seq from spleen (ENCLB710LIX) |
| H3K36me3 | GSE167840 | GSM5113887 | Spleen | Histone ChIP-seq from spleen (ENCLB325HKR) |
| H3K36me3 | GSE167850 | GSM5113986 | Tibial artery | Histone ChIP-seq from tibial artery (ENCLB287EMX) |
| H3K36me3 | GSE167850 | GSM5113987 | Tibial artery | Histone ChIP-seq from tibial artery (ENCLB804PGE) |
| H3K36me3 | GSE167854 | GSM5113997 | SJCRH30 | Histone ChIP-seq from SJCRH30 (ENCLB374BDE) |
| H3K36me3 | GSE167854 | GSM5113998 | SJCRH30 | Histone ChIP-seq from SJCRH30 (ENCLB711MBM) |
| H3K36me3 | GSE16256 | GSM521890 | IMR90 | Reference Epigenome: ChIP-Seq Analysis of H3K36me3 in IMR90 Cells; renlab.H3K36me3.IMR90-01.01 |
| H3K36me3 | GSE16256 | GSM521892 | IMR90 | Reference Epigenome: ChIP-Seq Analysis of H3K36me3 in IMR90 Cells; renlab.H3K36me3.IMR90-02.01 |
| H3K36me3 | GSE19465 | GSM537612 | CD3 | ChIP-Seq analysis of H3K36me3 in human CD3 cells; 30B91AAXX080917-1-S |
| H3K36me3 | GSE19465 | GSM537621 | CD19 | ChIP-Seq analysis of H3K36me3 in human CD19 cells; 30CAVAAXX081115-6-S |
| H3K36me3 | GSE19465 | GSM537630 | CD19 | ChIP-Seq analysis of H3K36me3 in human CD19 cells; 30EBJAAXX090206-5-S |
| H3K36me3 | GSE17312 | GSM537640 | WA-7 | ChIP-Seq analysis of H3K36me3 in human WA-7 cells; 30H88AAXX090617-3-S |
| H3K36me3 | GSE19465 | GSM537651 | CD34, Mobilized | ChIP-Seq analysis of H3K36me3 in human Mobilized CD34 cells; 42LNLAAXX090804-6-S |
| H3K36me3 | GSE19465 | GSM537655 | CD34, Cultured | ChIP-Seq analysis of H3K36me3 in human cultured CD34 cells; 42LP1AAXX090808-7-S |
| H3K36me3 | GSE19465 | GSM537661 | Pancreatic islets | ChIP-Seq analysis of H3K36me3 in human pancreatic islets normal cells; 42LTMAAXX090725-8-S |
| H3K36me3 | GSE19465 | GSM537666 | CD3 | ChIP-Seq analysis of H3K36me3 in human CD3 cells; 42M0GAAXX091022-8-S |
| H3K36me3 | GSE17312 | GSM537669 | hES-I3 | ChIP-Seq analysis of H3K36me3 in human hES-I3 cells; 42MNCAAXX100106-5-S |
| H3K36me3 | GSE17312 | GSM537672 | hES-I3 | ChIP-Seq analysis of H3K36me3 in human hES I3 TESR cells; 42NF9AAXX091210-1-S |
| H3K36me3 | GSE19465 | GSM537675 | hiPS-18c | ChIP-Seq analysis of H3K36me3 in human hiPS-18c cells; 42TPLAAXX100219-2-S |
| H3K36me3 | GSE17312 | GSM537684 | H1 hESC | ChIP-Seq analysis of H3K36me3 in human H1 cells; 42LR0AAXX091002-6-S |
| H3K36me3 | GSE19465 | GSM537690 | hiPS-15b | ChIP-Seq analysis of H3K36me3 in human hiPS-15b cells; 61BWEAAXX100226-5-S |
| H3K36me3 | GSE19465 | GSM537699 | hLiver | ChIP-Seq analysis of H3K36me3 in human hLiver cells; 61C9RAAXX100224-7-S |
| H3K36me3 | GSE19465 | GSM537704 | hiPS-20b | ChIP-Seq analysis of H3K36me3 in human hiPS-20b cells; 61GKJAAXX100226-8-S |
| H3K36me3 | GSE19465 | GSM537708 | hLiver | ChIP-Seq analysis of H3K36me3 in human hLiver cells; 61GKJAAXX100226-7-S |
| H3K36me3 | GSE16368 | GSM543035 | CD4 naive | H3K36me3 ChIP-Seq analysis of peripheral cd4 naive from TC001 (HS1989) |
| H3K36me3 | GSE20303 | GSM566172 | HeLa | H3K36me3_HeLa |
| H3K36me3 | GSE16256 | GSM605309 | H1 hESC | Reference Epigenome: ChIP-Seq Analysis of H3K36me3 in hESC Cells; renlab.H3K36me3.hESC-03.01 |
| H3K36me3 | GSE16256 | GSM605310 | H9 hESC | Reference Epigenome: ChIP-Seq Analysis of H3K36me3 in hESC H9 Cells; renlab.H3K36me3.hESC.H9.01.01 |
| H3K36me3 | GSE16368 | GSM613873 | Breast myoepithelial cell | H3K36me3 ChIP-Seq analysis of breast myoepithelial cell from RM066 (HS2443) |
| H3K36me3 | GSE16368 | GSM613880 | Peripheral mononuclear cell | H3K36me3 ChIP-Seq analysis of peripheral mononuclear cell from TC010 (HS2624) |
| H3K36me3 | GSE16368 | GSM613888 | Breast myoepithelial cell | H3K36me3 ChIP-Seq analysis of breast myoepithelial cell from RM080 (HS2763) |
| H3K36me3 | GSE19465 | GSM621387 | Fetal lung | ChIP-Seq analysis of H3K36me3 human fetal lung cells (superseded by GSE120318) |
| H3K36me3 | GSE19465 | GSM621392 | Fetal heart | ChIP-Seq analysis of H3K36me3 in human fetal heart cells; Lib:XZ:20100129:21--ChIP:XZ:20100125:21:fetal heart:H3K36me3 |
| H3K36me3 | GSE19465 | GSM621410 | Fetal brain | ChIP-Seq analysis of H3K36me3 in human fetal brain cells; Lib:XZ:20100129:16--ChIP:XZ:20100125:16:fetal brain:H3K36me3 |
| H3K36me3 | GSE19465 | GSM621415 | Fetal kidney | ChIP-Seq analysis of H3K36me3 in human fetal kidney cells; Lib:XZ:20100129:09--ChIP:XZ:20100125:09:fetal kidney:H3K36me3 |
| H3K36me3 | GSE19465 | GSM621419 | BM-MSC | ChIP-Seq analysis of H3K36me3 in human BM-MSC cells; Lib:MC:20100706:03--ChIP:MC:20100628:03:BM-MSC:H3K36Me3 |
| H3K36me3 | GSE19465 | GSM621421 | Duodenum mucosa | ChIP-Seq analysis of H3K36me3 in Duodenum mucosa cells; Lib:XZ:20100628:03--ChIP:XZ:20100621:03:Duodenum mucosa:H3K36Me3 |

| | | | | |
|---|---|---|---|---|
| H3K36me3 | GSE19465 | GSM621446 | ADMSC | ChIP-Seq analysis of H3K36me3 in human ADMSC cells; Lib:XZ:20100107:04--ChIP:XZ:20100104:02:ADMSC:H3K36Me3 |
| H3K36me3 | GSE19465 | GSM621449 | Treg | ChIP-Seq analysis of H3K36me3 in human Treg cells; Lib:RI:20100422:20--ChIP:MA:20100416:20:Treg:H3K36Me3 |
| H3K36me3 | GSE19465 | GSM621459 | CD34, Mobilized | ChIP-Seq analysis of H3K36me3 in Mobilized CD34 cells; Lib:RI:20100308:10--ChIP:MA:20100216:10:Mobilized CD34:H3K36Me3 |
| H3K36me3 | GSE19465 | GSM621461 | Adipose | ChIP-Seq analysis of H3K36me3 in AdiposeNuclei cells; Lib:XZ:20100107:09--ChIP:XZ:20100104:07:AdiposeNuclei:H3K36Me3 |
| H3K36me3 | GSE19465 | GSM621634 | Kidney | ChIP-Seq analysis of H3K36me3 in human adult kidney cells; Lib:XZ:20100423:14--ChIP:XZ:70100407:03:adult kidney:H3K36Me3 |
| H3K36me3 | GSE19465 | GSM621644 | Muscle | ChIP-Seq analysis of H3K36me3 in Skeletal muscle cells; Lib:XZ:20100423:04--ChIP:XZ:20100331:04:Skeletal muscle:H3K36Me3 |
| H3K36me3 | GSE19465 | GSM621646 | Mucosa | ChIP-Seq analysis of H3K36me3 in rectal mucosa cells; Lib:XZ:20100423:28--ChIP:XZ:70100407:17:rectal mucosa:H3K36Me3 |
| H3K36me3 | GSE19465 | GSM621649 | Stomach smooth muscle | ChIP-Seq analysis H3K36me3 stomach smooth muscle;Lib:XZ:20100423:21--ChIP:XZ:70100407:10:stomach smooth muscle:H3K36Me3 |
| H3K36me3 | GSE19465 | GSM621656 | Rectal smooth muscle | ChIP-Seq analysis of H3K36me3 rectal smooth muscle;Lib:XZ:20100503:03--ChIP:XZ:20100420:03:rectal smooth muscle:H3K36Me3 |
| H3K36me3 | GSE19465 | GSM621666 | CD34, Mobilized | ChIP-Seq analysis of H3K36me3; mobilized CD34 cells; Lib:RI:20100517:07--ChIP:Thanh:20100504:07:Mobilized CD34:H3K36Me3 |
| H3K36me3 | GSE19465 | GSM621672 | Colonic mucosa | ChIP-Seq analysis of H3K36me3 in colonic mucosa cells; Lib:XZ:20100513:03--ChIP:XZ:20100503:03:colonic mucosa:H3K36Me3 |
| H3K36me3 | GSE19465 | GSM621684 | Skeletal muscle | ChIP-Seq analysis of H3K36me3 in Skeletal muscle cells; Lib:XZ:20100628:10--ChIP:XZ:20100621:10:Skeletal muscle:H3K36Me3 |
| H3K36me3 | GSE19465 | GSM621687 | Rectal mucosa | ChIP-Seq analysis of H3K36me3 in rectal mucosa cells; Lib:XZ:20100503:10--ChIP:XZ:20100420:10:rectal mucosa:H3K36Me3 |
| H3K36me3 | GSE19465 | GSM621693 | Skeletal muscle | ChIP-Seq analysis of H3K36me3 in Skeletal muscle cells; Lib:XZ:20100628:17--ChIP:XZ:20100621:17:Skeletal muscle:H3K36Me3 |
| H3K36me3 | GSE25249 | GSM621739 | hSKM | ChIP-Seq analysis of H3K36me3 in human hSKM cells; Lib:MC:20100222:04--ChIP:MC:20100216:04:hSKM:H3K36Me3 |
| H3K36me3 | GSE25249 | GSM621749 | hSKM | ChIP-Seq analysis of H3K36me3 in human hSKM cells; Lib:MC:20100511:12--ChIP:MC:20100505:12:hSKM:H3K36Me3 |
| H3K36me3 | GSE25249 | GSM621754 | hSKM | ChIP-Seq analysis of H3K36me3 in human hSKM cells; Lib:MC:20100222:11--ChIP:MC:20100216:11:hSKM:H3K36Me3 |
| H3K36me3 | GSE16256 | GSM667623 | H9 hESC | Reference Epigenome: ChIP-Seq Analysis of H3K36me3 in hESC H9 Cells; renlab.H3K36me3.hESC.H9.02.01 |
| H3K36me3 | GSE16368 | GSM669593 | CD8 naive | H3K36me3 ChIP-Seq analysis of peripheral cd8 naive from TC001 (HS2387) |
| H3K36me3 | GSE16368 | GSM669597 | Luminal epithelial cell | H3K36me3 ChIP-Seq analysis of breast luminal epithelial cell from RM080 (HS2795) |
| H3K36me3 | GSE17312 | GSM669892 | HUES6 | ChIP-Seq analysis of H3K36me3 in HUES 6 cells; Lib:XZ:20101004:10--ChIP:XZ:20100930:10:ES cell HUES_6:H3K36Me3 |
| H3K36me3 | GSE17312 | GSM669898 | HUES6 | ChIP-Seq analysis of H3K36me3 in HUES 6 cells; Lib:XZ:20101004:03--ChIP:XZ:20100930:03:ES cell HUES_6:H3K36Me3 |
| H3K36me3 | GSE19465 | GSM669912 | Fetal lung | ChIP-Seq analysis of H3K36me3 in fetal lung cells; Lib:XZ:20100731:02--ChIP:XZ:20100726:02:fetal lung:H3K36Me3 |
| H3K36me3 | GSE19465 | GSM669914 | Chondrocytes derived via in vitro culture from MSC | ChIP-Seq analysis of H3K36me3; chondr. dif cls; Lib:MC:20100729:03--ChIP:MC:20100726:03:chondrogenic dif cells:H3K36Me3 |
| H3K36me3 | GSE19465 | GSM669931 | hLiver | ChIP-Seq analysis of H3K36me3 in hLiver cells; Lib:XZ:20100827:02--ChIP:XZ:20100824:02:hLiver:H3K36Me3 |
| H3K36me3 | GSE19465 | GSM669932 | HUES64 | ChIP-Seq analysis of H3K36me3 in HUES64 cells; Lib:MC:20101025:03--ChIP:MC:20101020:03:ES cell HUES_64:H3K36Me3 |
| H3K36me3 | GSE19465 | GSM669935 | Chondrocyte | ChIP-Seq analysis of H3K36me3; chondr. dif cls; Lib:MC:20100729:10--ChIP:MC:20100726:10:chondrogenic dif cells:H3K36Me3 |
| H3K36me3 | GSE19465 | GSM669947 | Substantia nigra | ChIP-Seq analysis of H3K36me3; substantia nigra cells; Lib:XZ:20100921:03--ChIP:XZ:20100914:03:substantia nigra:H3K36Me3 |
| H3K36me3 | GSE19465 | GSM669951 | BM-MSC | ChIP-Seq analysis of H3K36me3 in BM-MSC cells; Lib:MC:20100713:10--ChIP:MC:20100706:10:BM-MSC:H3K36Me3 |
| H3K36me3 | GSE19465 | GSM669952 | Adipose | ChIP-Seq analysis of H3K36me3 in AdiposeNuclei cells; Lib:XZ:20100731:03--ChIP:XZ:20100726:03:AdiposeNuclei:H3K36Me3 |
| H3K36me3 | GSE19465 | GSM669955 | CD34, cultured | ChIP-Seq analysis of H3K36me3 in Cultured CD34 cells; Lib:RI:20101012:05--ChIP:MA:20100927:05:CD34 expanded:H3K36Me3 |
| H3K36me3 | GSE19465 | GSM669979 | Adipose | ChIP-Seq analysis of H3K36me3 in AdiposeNuclei cells; Lib:XZ:20100731:17--ChIP:XZ:20100726:17:AdiposeNuclei:H3K36Me3 |
| H3K36me3 | GSE19465 | GSM669982 | Mid frontal lobe | ChIP-Seq analysis of H3K36me3; mid frontal lobe cls; Lib:XZ:20100924:03--ChIP:XZ:20100914:03:mid frontal lobe:H3K36Me3 |
| H3K36me3 | GSE19465 | GSM669985 | Cingulate gyrius | ChIP-Seq analysis of H3K36me3 in cingulate gyrus cells; Lib:XZ:20100924:10--ChIP:XZ:20100914:10:cingulate gyrus:H3K36Me3 |
| H3K36me3 | GSE19465 | GSM669996 | BM-MSC | ChIP-Seq analysis of H3K36me3 in BM-MSC cells; Lib:MC:20100713:03--ChIP:MC:20100706:03:BM-MSC:H3K36Me3 |
| H3K36me3 | GSE19465 | GSM669997 | Adipose | ChIP-Seq analysis of H3K36me3 in AdiposeNuclei cells; Lib:XZ:20100731:24--ChIP:XZ:20100726:24:AdiposeNuclei:H3K36Me3 |
| H3K36me3 | GSE19465 | GSM670002 | Hippocampus mid | ChIP-Seq analysis of H3K36me3; hippocampus mid cls; Lib:XZ:20100921:10--ChIP:XZ:20100914:10:hippocampus middle:H3K36Me3 |
| H3K36me3 | GSE19465 | GSM670011 | Temporal lobe | ChIP-Seq analysis of H3K36me3;inf temp lobe cls; Lib:XZ:20100924:17--ChIP:XZ:20100914:17:inferior temporal lobe:H3K36Me3 |
| H3K36me3 | GSE19465 | GSM670012 | Chondrocyte | ChIP-Seq analysis of H3K36me3; chondr. dif cls; Lib:MC:20100726:03--ChIP:MC:20100713:03:chondrogenic dif cells:H3K36Me3 |
| H3K36me3 | GSE19465 | GSM670014 | Adipose | ChIP-Seq analysis of H3K36me3 in AdiposeNuclei cells; Lib:XZ:20100731:10--ChIP:XZ:20100726:10:AdiposeNuclei:H3K36Me3 |
| H3K36me3 | GSE19465 | GSM670026 | Anterior caudate cells | ChIP-Seq analysis of H3K36me3; ant. caudate cls; Lib:XZ:20100921:18--ChIP:XZ:20100914:18:anterior caudate |

| | | | | |
|---|---|---|---|---|
| H3K36me3 | GSE19465 | GSM670037 | BM-MSC | ChIP-Seq analysis of H3K36me3 in BM-MSC cells; Lib:MC:20100706:10--ChIP:MC:20100628:10:BM-MSC:H3K36Me3 |
| H3K36me3 | GSE19465 | GSM670039 | HUES48 | ChIP-Seq analysis of H3K36me3 in HUES48 cells; Lib:MC:20101025:09--ChIP:MC:20101020:09:ES cell HUES_48:H3K36Me3 |
| H3K36me3 | GSE16368 | GSM693275 | Breast variant human mammary epithelial cell | H3K36me3 ChIP-Seq analysis of breast variant human mammary epithelial cell from RM035 (HS1993) |
| H3K36me3 | GSE16256 | GSM706069 | iPS 19.11 | Reference Epigenome: ChIP-Seq Analysis of H3K36me3 in iPS 19.11 Cells; renlab.H3K36me3.iPS-19.11_01.01 |
| H3K36me3 | GSE16256 | GSM706070 | iPS 6.9 | Reference Epigenome: ChIP-Seq Analysis of H3K36me3 in iPS 6.9 Cells; renlab.H3K36me3.iPS-6.9_01.01 |
| H3K36me3 | GSE17312 | GSM706843 | CD34, Mobilized | ChIP-Seq analysis of H3K36me3 in human Mobilized CD34 cells; DNA_Lib 261 |
| H3K36me3 | GSE16368 | GSM707002 | Brain (germinal matrix) | H3K36me3 ChIP-Seq analysis of brain (germinal matrix) tissue from HuFGM01 (A03391) |
| H3K36me3 | GSE16368 | GSM707007 | Cortex neural stem and progenitor cells | H3K36me3 ChIP-Seq analysis of cortex neural stem and progenitor cells from HuFNSC02 (A03285) |
| H3K36me3 | GSE16368 | GSM707012 | Ganglionic eminence neural stem and progenitor cells | H3K36me3 ChIP-Seq analysis of ganglionic eminence neural stem and progenitor cells from HuFNSC02 (A03481) |
| H3K36me3 | GSE16256 | GSM752972 | Mesendoderm differentiated from H1 hESC | Reference Epigenome: ChIP-Seq Analysis of H3K36me3 in BMP4 Mesendoderm Cells; renlab.H3K36me3.BMP4M.01.01 |
| H3K36me3 | GSE16256 | GSM752973 | Mesendoderm differentiated from H1 hESC | Reference Epigenome: ChIP-Seq Analysis of H3K36me3 in BMP4 Mesendoderm Cells; renlab.H3K36me3.BMP4M.02.01 |
| H3K36me3 | GSE16256 | GSM752974 | iPS 19.11 | Reference Epigenome: ChIP-Seq Analysis of H3K36me3 in iPS 19.11 Cells; renlab.H3K36me3.iPS-19.11.02.01 |
| H3K36me3 | GSE16256 | GSM752975 | iPS 6.9 | Reference Epigenome: ChIP-Seq Analysis of H3K36me3 in iPS 6.9 Cells; renlab.H3K36me3.iPS-6.9.02.01 |
| H3K36me3 | GSE16256 | GSM753432 | Trophoblast differentiated from H1 hESC | Reference Epigenome: ChIP-Seq Analysis of H3K36me3 in BMP4 Trophoblast Cells; renlab.H3K36me3.BMP4T.01.01 |
| H3K36me3 | GSE16256 | GSM753433 | Trophoblast differentiated from H1 hESC | Reference Epigenome: ChIP-Seq Analysis of H3K36me3 in BMP4 Trophoblast Cells; renlab.H3K36me3.BMP4T.02.01 |
| H3K36me3 | GSE16256 | GSM753434 | MSC | Reference Epigenome: ChIP-Seq Analysis of H3K36me3 in Mesenchymal Stem Cells; renlab.H3K36me3.MSC.01.01 |
| H3K36me3 | GSE29282 | GSM763416 | OCI-LY1 | OCI-LY1_H3K36me3_CHIP |
| H3K36me3 | GSE30895 | GSM766169 | HeLa | H3K36me3 ChIP-seq |
| H3K36me3 | GSE30895 | GSM766170 | HeLa | H3K36me3 ChIP-seq in SSA treated cells |
| H3K36me3 | GSE16256 | GSM767346 | MSC | Reference Epigenome: ChIP-Seq Analysis of H3K36me3 in Mesenchymal Stem Cells; renlab.H3K36me3.MSC.02.01 |
| H3K36me3 | GSE17312 | GSM772743 | ADMSC | ChIP-Seq analysis of H3K36me3 in human ADMSC Diff Day20 cells; Lib EpiLabSPL Row 1752 |
| H3K36me3 | GSE17312 | GSM772751 | HUES64 | ChIP-Seq analysis of H3K36me3 in human HUES64 cells; Lib EpiLabSPL Row 1721 |
| H3K36me3 | GSE17312 | GSM772763 | ADMSC | ChIP-Seq analysis of H3K36me3 in human ADMSC undiff Day0 cells; Lib EpiLabSPL Row 1746 |
| H3K36me3 | GSE17312 | GSM772768 | Angular gyrus cells | ChIP-Seq analysis of H3K36me3 in human angular gyrus cells; Lib EpiLabSPL Row 1838 |
| H3K36me3 | GSE17312 | GSM772795 | Neural progenitors derived from H9 hESC | ChIP-Seq analysis H3K36me3 hNP; Lib:RI:20101012:11--ChIP:MA:20100927:11:hNP (ES H9 derived Nuronal Progenitors):H3K36Me3 |
| H3K36me3 | GSE17312 | GSM772798 | HUES48 | ChIP-Seq analysis of H3K36me3 in human HUES48 cells; Lib EpiLabSPL Row 1715 |
| H3K36me3 | GSE17312 | GSM772803 | Neurons derived from H9 hESC | ChIP-Seq analysis of H3K36me3 in hN cells; Lib:RI:20101012:17--ChIP:MA:20100927:17:hN (ES H9 derived Neurons):H3K36Me3 |
| H3K36me3 | GSE17312 | GSM772814 | CD15 | ChIP-Seq analysis of H3K36me3 in human CD15 cells; Lib EpiLabSPL Row 1800 |
| H3K36me3 | GSE17312 | GSM772818 | ADMSC | ChIP-Seq analysis of H3K36me3 in human ADMSC Diff Day20 cells; Lib EpiLabSPL Row 1736 |
| H3K36me3 | GSE17312 | GSM772820 | ADMSC | ChIP-Seq analysis of H3K36me3 in human ADMSC undiff Day0 cells; Lib EpiLabSPL Row 1730 |
| H3K36me3 | GSE17312 | GSM772825 | Chondrocytes derived via in vitro culture from MSC | ChIP-Seq analysis of H3K36me3 in human chondrogenic dif cells; Lib EpiLabSPL Row 1388 |
| H3K36me3 | GSE17312 | GSM772828 | Anterior caudate cells | ChIP-Seq analysis of H3K36me3 in human anterior caudate cells; DNA_Lib 601 |
| H3K36me3 | GSE17312 | GSM772838 | Duodenum smooth muscle cells | ChIP-Seq analysis of H3K36me3 in human Duodenum smooth muscle cells; DNA_Lib 458 |
| H3K36me3 | GSE17312 | GSM772846 | hiPS-20b | ChIP-Seq analysis of H3K36me3 in human hiPS-20b cells; DNA_Lib 355 |
| H3K36me3 | GSE17312 | GSM772865 | CD34, Mobilized | ChIP-Seq analysis of H3K36me3 in human Mobilized CD34 cells; DNA_Lib 257 |
| H3K36me3 | GSE17312 | GSM772866 | CD4+ CD25- T Cells | ChIP-Seq analysis of H3K36me3 in human CD25- Th cells; DNA_Lib 361 |
| H3K36me3 | GSE17312 | GSM772872 | CD8 Naive Cells | ChIP-Seq analysis of H3K36me3 in human CD45RA CD8 cells; DNA_Lib 667 |
| H3K36me3 | GSE17312 | GSM772877 | CD8+ memory T cells | ChIP-Seq analysis of H3K36me3 in human CD45RO CD8 cells; DNA_Lib 545 |
| H3K36me3 | GSE17312 | GSM772891 | Stomach smooth muscle cells | ChIP-Seq analysis of H3K36me3 in human stomach smooth muscle cells; DNA_Lib 377 |
| H3K36me3 | GSE17312 | GSM772892 | CD4+ CD25- T Cells | ChIP-Seq analysis of H3K36me3 in human CD25- IL17- Th stim MACS cells; DNA_Lib 711 |
| H3K36me3 | GSE17312 | GSM772893 | CD4+ CD25- CD45RO+ memory T Cells | ChIP-Seq analysis of H3K36me3 in human CD25- CD45RO+ mem cells; DNA_Lib 718 |
| H3K36me3 | GSE17312 | GSM772895 | Naive CD8 T cells | ChIP-Seq analysis of H3K36me3 in human CD45RA CD8 cells; DNA_Lib 499 |
| H3K36me3 | GSE17312 | GSM772912 | CD184+ endoderm differentiated in vitro from HUES64 | ChIP-Seq analysis of H3K36me3 in human HUES64 derived CD184+ cells; DNA_Lib 263 |
| H3K36me3 | GSE17312 | GSM772918 | Naive CD4 T cells | ChIP-Seq analysis of H3K36me3 in human CD45RA CD4 cells; DNA_Lib 525 |
| H3K36me3 | GSE17312 | GSM772923 | CD4+ CD25- CD45RA+ naive T Cells | ChIP-Seq analysis of H3K36me3 in human CD25- CD45RA+ naive cells; DNA_Lib 725 |
| H3K36me3 | GSE17312 | GSM772926 | CD4+ CD45RO+ memory T cells | ChIP-Seq analysis of H3K36me3 in human CD45RO CD4 cells; DNA_Lib 483 |

| | | | | |
|---|---|---|---|---|
| H3K36me3 | GSE17312 | GSM772932 | Naive CD4 T cells | ChIP-Seq analysis of H3K36me3 in human CD45RA CD4 cells; DNA_Lib 660 |
| H3K36me3 | GSE17312 | GSM772936 | Stomach mucosa cells | ChIP-Seq analysis of H3K36me3 in human stomach mucosa cells; DNA_Lib 319 |
| H3K36me3 | GSE17312 | GSM772945 | CD4+ CD25+ CD127- Treg | ChIP-Seq analysis of H3K36me3 in human CD25+ CD127- Treg cells; DNA_Lib 312 |
| H3K36me3 | GSE17312 | GSM772957 | CD8+ memory T cells | ChIP-Seq analysis of H3K36me3 in human CD45RO CD8 cells; DNA_Lib 532 |
| H3K36me3 | GSE17312 | GSM772961 | Angular gyrus cells | ChIP-Seq analysis of H3K36me3 in human angular gyrus cells; DNA_Lib 631 |
| H3K36me3 | GSE17312 | GSM772964 | CD4+ CD45RO+ memory T cells | ChIP-Seq analysis of H3K36me3 in human CD45RO CD4 cells; DNA_Lib 539 |
| H3K36me3 | GSE17312 | GSM772975 | Colon smooth muscle cells | ChIP-Seq analysis of H3K36me3 in human colon smooth muscle cells; DNA_Lib 326 |
| H3K36me3 | GSE17312 | GSM772981 | CD4+ CD25int CD127+ T memory cell | ChIP-Seq analysis of H3K36me3 in human CD25int CD127+ Tmem cells; DNA_Lib 732 |
| H3K36me3 | GSE17312 | GSM772982 | Inferior temporal lobe cells | ChIP-Seq analysis of H3K36me3 in human inferior temporal lobe cells; DNA_Lib 628 |
| H3K36me3 | GSE17312 | GSM773000 | Kidney | ChIP-Seq analysis of H3K36me3 in human kidney cells; DNA_Lib 337 |
| H3K36me3 | GSE17312 | GSM773009 | Cingulate gyrus cells | ChIP-Seq analysis of H3K36me3 in human cingulate gyrus cells; DNA_Lib 615 |
| H3K36me3 | GSE17312 | GSM773013 | Mid frontal lobe cells | ChIP-Seq analysis of H3K36me3 in human mid frontal lobe cells; DNA_Lib 610 |
| H3K36me3 | GSE17312 | GSM773018 | Hippocampus middle cells | ChIP-Seq analysis of H3K36me3 in human hippocampus middle cells; DNA_Lib 583 |
| H3K36me3 | GSE17312 | GSM773027 | hiPS-18a | ChIP-Seq analysis of H3K36me3 in human hiPS-18a cells; DNA_Lib 347 |
| H3K36me3 | GSE17312 | GSM773042 | CD34, Mobilized | ChIP-Seq analysis of H3K36me3 in Mobilized CD34 cells; Lib:RI:20100603:12--ChIP:MA:20100517:12:Mobilized CD34:H3K36Me3 |
| H3K36me3 | GSE16368 | GSM806938 | Fetal brain | H3K36me3 ChIP-Seq analysis of fetal brain tissue from HuFNSC02 (A03497) |
| H3K36me3 | GSE16368 | GSM806946 | Fetal brain | H3K36me3 ChIP-Seq analysis of fetal brain tissue from HuFNSC01 (A03489) |
| H3K36me3 | GSE16368 | GSM817225 | Foreskin keratinocyte | H3K36me3 ChIP-Seq analysis of foreskin keratinocyte from skin01 (A05018) |
| H3K36me3 | GSE16368 | GSM817227 | Brain (germinal matrix) | H3K36me3 ChIP-Seq analysis of brain (germinal matrix) tissue from HuFGM02 (A03505) |
| H3K36me3 | GSE16368 | GSM817238 | Foreskin fibroblast | H3K36me3 ChIP-Seq analysis of foreskin fibroblast from skin01 (A08393) |
| H3K36me3 | GSE16368 | GSM817241 | Foreskin fibroblast | H3K36me3 ChIP-Seq analysis of foreskin fibroblast from skin02 (A08399) |
| H3K36me3 | GSE16256 | GSM818034 | NPC | Reference Epigenome: ChIP-Seq Analysis of H3K36me3 in Neural Progenitor Cells; renlab.H3K36me3.NPC.01.01 |
| H3K36me3 | GSE16256 | GSM818035 | NPC | Reference Epigenome: ChIP-Seq Analysis of H3K36me3 in Neural Progenitor Cells; renlab.H3K36me3.NPC.02.01 |
| H3K36me3 | GSE33887 | GSM838387 | HeLa | h3k36me3 sictrl ChIP-Seq |
| H3K36me3 | GSE33887 | GSM838388 | HeLa | h3k36me3 si23 ChIP-Seq |
| H3K36me3 | GSE16256 | GSM906401 | Embryonic stem cell differentiated into trophoblas | Reference Epigenome: ChIP-Seq Analysis of H3K36me3 in BMP4 Trophoblast Cells; renlab.H3K36me3.BMP4T.03.01 |
| H3K36me3 | GSE16256 | GSM906402 | Left ventricle | Reference Epigenome: ChIP-Seq Analysis of H3K36me3 in Human Left Ventricle Tissue; renlab.H3K36me3.STL001LV.01.01 |
| H3K36me3 | GSE16256 | GSM910565 | Small bowel | Reference Epigenome: ChIP-Seq Analysis of H3K36me3 in Human Small Bowel Tissue; renlab.H3K36me3.STL001SB.01.01 |
| H3K36me3 | GSE16256 | GSM910566 | Aorta | Reference Epigenome: ChIP-Seq Analysis of H3K36me3 in Human Aorta Tissue; renlab.H3K36me3.STL003AO.01.01 |
| H3K36me3 | GSE16256 | GSM910567 | Esophagus | Reference Epigenome: ChIP-Seq Analysis of H3K36me3 in Human Esophagus Tissue; renlab.H3K36me3.STL003EG.01.01 |
| H3K36me3 | GSE16256 | GSM910568 | Gastric | Reference Epigenome: ChIP-Seq Analysis of H3K36me3 in Human Gastric Tissue; renlab.H3K36me3.STL003GA.01.01 |
| H3K36me3 | GSE16256 | GSM910569 | Left ventricle | Reference Epigenome: ChIP-Seq Analysis of H3K36me3 in Human Left Ventricle Tissue; renlab.H3K36me3.STL003LV.01.01 |
| H3K36me3 | GSE16256 | GSM910570 | Pancreas | Reference Epigenome: ChIP-Seq Analysis of H3K36me3 in Human Pancreas Tissue; renlab.H3K36me3.STL003PA.01.01 |
| H3K36me3 | GSE16256 | GSM910571 | Spleen | Reference Epigenome: ChIP-Seq Analysis of H3K36me3 in Human Spleen Tissue; renlab.H3K36me3.STL003SX.01.01 |
| H3K36me3 | GSE17312 | GSM916015 | Substantia nigra cells | ChIP-Seq analysis of H3K36me3 in human substantia nigra cells; DNA_Lib 554 |
| H3K36me3 | GSE17312 | GSM916020 | Duodenum mucosa cells | ChIP-Seq analysis of H3K36me3 in human Duodenum mucosa cells; DNA_Lib 375 |
| H3K36me3 | GSE17312 | GSM916028 | CD4+ CD25- IL17+ Th17 stim | ChIP-Seq analysis of H3K36me3 in human CD25- IL17+ Th17 stim cells; DNA_Lib 739 |
| H3K36me3 | GSE17312 | GSM916031 | Colon smooth muscle cells | ChIP-Seq analysis of H3K36me3 in human colon smooth muscle cells; DNA_Lib 928 |
| H3K36me3 | GSE17312 | GSM916036 | Duodenum smooth muscle cells | ChIP-Seq analysis of H3K36me3 in human Duodenum smooth muscle cells; DNA_Lib 987 |
| H3K36me3 | GSE17312 | GSM916041 | Hippocampus middle cells | ChIP-Seq analysis of H3K36me3 in human hippocampus middle cells; DNA_Lib 982 |
| H3K36me3 | GSE17312 | GSM916042 | Colonic mucosa cells | ChIP-Seq analysis of H3K36me3 in human colonic mucosa cells; DNA_Lib 365 |
| H3K36me3 | GSE17312 | GSM916068 | CD56+ mesoderm cells | ChIP-Seq analysis of H3K36me3 in human CD56+ mesoderm cells; DNA_Lib 1283 |
| H3K36me3 | GSE17312 | GSM916072 | CD56+ mesoderm cells | ChIP-Seq analysis of H3K36me3 in human CD56+ mesoderm cells; DNA_Lib 1277 |
| H3K36me3 | GSE16368 | GSM941714 | cortex neural stem and progenitor cells | H3K36me3 ChIP-Seq analysis of cortex neural stem and progenitor cells from HuFNSC01 (A03273) |
| H3K36me3 | GSE16368 | GSM941716 | cortex neural stem and progenitor cells | H3K36me3 ChIP-Seq analysis of ganglionic eminence neural stem and progenitor cells from HuFNSC01 (A03279) |
| H3K36me3 | GSE16368 | GSM941729 | Foreskin melanocyte | H3K36me3 ChIP-Seq analysis of foreskin melanocyte from skin01 (A08387) |
| H3K36me3 | GSE16368 | GSM941734 | Foreskin melanocyte | H3K36me3 ChIP-Seq analysis of foreskin melanocyte from skin02 (A08747) |
| H3K36me3 | GSE16368 | GSM941739 | Foreskin keratinocyte | H3K36me3 ChIP-Seq analysis of foreskin keratinocyte from skin02 (A11924) |
| H3K36me3 | GSE16368 | GSM941752 | UCSF-4 | H3K36me3 ChIP-Seq analysis of UCSF-4 embryonic stem cell line (A14673) |
| H3K36me3 | GSE38442 | GSM942101 | IMR90 | Chip Seq H3K36me3 in ER:Ras-induced senescent IMR90 with Lenti-sh-HMGA1 SLX3614 |
| H3K36me3 | GSE38442 | GSM942102 | IMR90 | Chip Seq H3K36me3 in ER:Ras expressing IMR90 with no treatment SLX3168 |
| H3K36me3 | GSE38442 | GSM942103 | IMR90 | Chip Seq H3K36me3 in ER:Ras expressing IMR90 with no treatment SLX934 |
| H3K36me3 | GSE38442 | GSM942104 | IMR90 | Chip Seq H3K36me3 in ER:Ras expressing IMR90 d6 4OHT with sh-HMGA1/MSCV SLX3602 |
| H3K36me3 | GSE38442 | GSM942105 | IMR90 | Chip Seq H3K36me3 in ER:Ras expressing IMR90 d6 4OHT with sh-Rb SLX3610 |
| H3K36me3 | GSE38442 | GSM942106 | IMR90 | Chip Seq H3K36me3 in ER:Ras expressing IMR90 with no 4OHT with sh-Rb SLX3606 |
| H3K36me3 | GSE38442 | GSM942107 | IMR90 | Chip Seq H3K36me3 in ER:Ras expressing IMR90 with no 4OHT with sh-HMGA1/MSCV SLX3598 |

| | | | | |
|---|---|---|---|---|
| H3K36me3 | GSE38442 | GSM942108 | IMR90 | Chip Seq H3K36me3 in ER:Ras expressing IMR90 d6 4OHT SLX3169 |
| H3K36me3 | GSE38442 | GSM942109 | IMR90 | Chip Seq H3K36me3 in ER:Ras expressing IMR90 d6 4OHT SLX936 |
| H3K36me3 | GSE16256 | GSM956012 | Trophoblast differentiated from H1 hESC | Reference Epigenome: ChIP-Seq Analysis of H3K36me3 in BMP4 Trophoblast Cells; renlab.H3K36me3.BMP4T.04.01 |
| H3K36me3 | GSE16256 | GSM956013 | Sigmoid colon | Reference Epigenome: ChIP-Seq Analysis of H3K36me3 in Human Sigmoid Colon Tissue; renlab.H3K36me3.STL001SG.01.01 |
| H3K36me3 | GSE16256 | GSM956014 | Lung | Reference Epigenome: ChIP-Seq Analysis of H3K36me3 in Human Lung Tissue; renlab.H3K36me3.STL002LG.01.01 |
| H3K36me3 | GSE16256 | GSM956015 | Adrenal gland | Reference Epigenome: ChIP-Seq Analysis of H3K36me3 in Human Adrenal Gland Tissue; renlab.H3K36me3.STL003AD.01.01 |
| H3K36me3 | GSE16256 | GSM956016 | Sigmoid colon | Reference Epigenome: ChIP-Seq Analysis of H3K36me3 in Human Sigmoid Colon Tissue; renlab.H3K36me3.STL003SG.01.01 |
| H3K36me3 | GSE16368 | GSM958149 | Foreskin fibroblast | H3K36me3 ChIP-Seq analysis of foreskin fibroblast from skin03 (A15569) |
| H3K36me3 | GSE16368 | GSM958159 | Foreskin keratinocyte | H3K36me3 ChIP-Seq analysis of foreskin keratinocyte from skin03 (A15576) |
| H3K36me3 | GSE16368 | GSM958160 | Foreskin melanocyte | H3K36me3 ChIP-Seq analysis of foreskin melanocyte from skin03 (A15583) |
| H3K36me3 | GSE40195 | GSM988181 | Sperm | Sperm Donor Pool H3K36me3 ChIP-seq |
| H3K36me3 | GSE17312 | GSM997226 | CD4+ CD25- IL17+ Th17 stim | ChIP-Seq analysis of H3K36me3 in human CD25- IL17+ Th17 stim cells; DNA_Lib 2215 |
| H3K36me3 | GSE17312 | GSM997230 | CD4+ CD25+ CD127- Treg | ChIP-Seq analysis of H3K36me3 in human CD25+ CD127- Treg cells; DNA_Lib 2090 |
| H3K36me3 | GSE17312 | GSM997237 | CD4+ CD25- CD45RO+ memory T cell | ChIP-Seq analysis of H3K36me3 in human CD25- CD45RO+ mem cells; DNA_Lib 2097 |
| H3K36me3 | GSE17312 | GSM997241 | CD4+ CD25- T Cells | ChIP-Seq analysis of H3K36me3 in human CD25- Th cells; DNA_Lib 2271 |
| H3K36me3 | GSE17312 | GSM997253 | CD56+ ectoderm | ChIP-Seq analysis of H3K36me3 in human CD56+ ectoderm cells; DNA_Lib 1808 |
| H3K36me3 | GSE17312 | GSM997255 | CD4+ CD25- CD45RA+ naive T cell | ChIP-Seq analysis of H3K36me3 in human CD25- CD45RA+ naive cells; DNA_Lib 2209 |
| H3K36me3 | GSE17312 | GSM997264 | CD4+ CD25int CD127+ memory T cell | ChIP-Seq analysis of H3K36me3 in human CD25int CD127+ Tmem cells; DNA_Lib 2265 |
| H3K36me3 | GSE17312 | GSM997274 | CD4+ CD25- IL17+ Th17 stim | ChIP-Seq analysis of H3K36me3 in human CD25- IL17- Th stim MACS cells; DNA_Lib 2259 |
| H3K36me3 | GSE17312 | GSM997276 | Colon smooth muscle cells | ChIP-Seq analysis of H3K36me3 in human colon smooth muscle cells; DNA_Lib 1851 |

## Table S4. Pri-miRNA annotations not included in Bouvy-Liivrand et al, 2017

| Pri-miRNA name | TSSid | Chr | Start | End | Strand | Host gene | Other annot |
|---|---|---|---|---|---|---|---|
| hsa-mir-5087_15 | chr1_147931980_-_2059 | chr1 | 147781101 | 147931980 | - | NBPF19:NM_001351365 | |
| hsa-mir-5087_16 | chr1_148011788_-_2059 | chr1 | 147955419 | 148011788 | - | NBPF19:NM_001351365 | |
| hsa-mir-5087_17 | chr1_148176401_-_2059 | chr1 | 148025863 | 148176401 | - | NBPF19:NM_001351365 | |
| hsa-mir-5087_18 | chr1_148271900_-_2059 | chr1 | 148269609 | 148271900 | - | NBPF19:NM_001351365 | |
| hsa-mir-5087_19 | chr1_148282340_-_2059 | chr1 | 148277551 | 148282340 | - | NBPF11:uc001eqg | |
| hsa-mir-5087_20 | chr1_148285533_-_2059 | chr1 | 148282340 | 148285533 | - | NBPF19:NM_001351365 | |
| hsa-mir-5087_21 | chr1_148310085_-_2059 | chr1 | 148298262 | 148310085 | - | NBPF19:NM_001351365 | |
| hsa-mir-5087_22 | chr1_148346791_-_2059 | chr1 | 148338469 | 148346791 | - | NBPF19:NM_001351365 | |
| hsa-mir-5698_1 | chr1_154108448_-_2157 | chr1 | 153965168 | 154108448 | - | NUP210L:uc009woq | |
| hsa-mir-5698_2 | chr1_154127592_-_2157 | chr1 | 154108448 | 154127592 | - | NUP210L:uc001fdw | |
| hsa-mir-4259_2 | chr1_159869906_-_2243 | chr1 | 159863095 | 159869906 | - | | |
| hsa-mir-921_2 | chr1_166135992_-_2307 | chr1 | 166039596 | 166135992 | - | | |
| hsa-mir-3119-2_1 | chr1_170115188_+_999 | chr1 | 170115188 | 170136923 | + | METTL11B:NM_001136107 | |
| hsa-mir-1295b_1 | chr1_171060018_+_1004 | chr1 | 171060018 | 171086959 | + | | |
| hsa-mir-199a-2_hsa-mir-214_1 | chr1_172113975_-_2340 | chr1 | 172106019 | 172113975 | - | | |
| hsa-mir-3972_1 | chr1_17575593_+_159 | chr1 | 17575593 | 17610727 | + | PADI3:NM_016233 | |
| hsa-mir-488_1 | chr1_177001985_-_2359 | chr1 | 176945189 | 177001985 | - | | |
| hsa-mir-488_2 | chr1_177134024_-_2359 | chr1 | 177001985 | 177134024 | - | | |
| hsa-mir-205_1 | chr1_209602168_+_1177 | chr1 | 209602168 | 209605892 | + | MIR205HG:uc009xcn | |
| hsa-mir-4677_3 | chr1_243419307_+_1317 | chr1 | 243419307 | 243449574 | + | SDCCAG8:NM_001350246 | |
| hsa-mir-4677_2 | chr1_243449574_+_1317 | chr1 | 243449574 | 243456393 | + | SDCCAG8:uc001hzx | |
| hsa-mir-4677_1 | chr1_243456393_+_1317 | chr1 | 243456393 | 243663393 | + | SDCCAG8:uc001hzy | |
| hsa-mir-6507_1 | chr10_100995632_-_3681 | chr10 | 100216834 | 100995632 | - | | |
| hsa-mir-936_2 | chr10_105845638_-_3726 | chr10 | 105792048 | 105845638 | - | | |
| hsa-mir-6715a_1 | chr10_114043414_+_3191 | chr10 | 114043414 | 114064792 | + | TECTB:NM_058222 | |
| hsa-mir-3663_1 | chr10_118928566_-_3760 | chr10 | 118918167 | 118928566 | - | | |
| hsa-mir-4293_5 | chr10_14504143_-_3344 | chr10 | 14372883 | 14504143 | - | | |
| hsa-mir-511-1_hsa-mir-511-2_1 | chr10_17851342_+_2754 | chr10 | 17851342 | 18041227 | + | MRC1:NM_002438 | |
| hsa-mir-3611_2 | chr10_35379311_-_3413 | chr10 | 35363258 | 35379311 | - | | |
| hsa-mir-3156-1_2 | chr10_45642842_+_2841 | chr10 | 45642842 | 45650110 | + | | |
| hsa-mir-3156-1_1 | chr10_45650110_+_2841 | chr10 | 45650110 | 45681489 | + | | |
| hsa-mir-548f-1_1 | chr10_56424050_-_3489 | chr10 | 55568452 | 56424050 | - | PCDH15:uc021pqv | AC013737 |
| hsa-mir-34b_hsa-mir-34c_1 | chr11_111381327_+_4478 | chr11 | 111381327 | 111384613 | + | | |
| hsa-mir-4301_2 | chr11_113346001_-_5392 | chr11 | 113295694 | 113346001 | - | | |
| hsa-mir-6716_3 | chr11_118498021_+_4530 | chr11 | 118498021 | 118502630 | + | PHLDB1:uc001p | |
| hsa-mir-6716_2 | chr11_118502630_+_4530 | chr11 | 118502630 | 118512269 | + | PHLDB1:uc009zai | |
| hsa-mir-6716_1 | chr11_118512269_+_4530 | chr11 | 118512269 | 118528748 | + | PHLDB1:uc010ryi | |
| hsa-mir-4486_7 | chr11_19372271_+_3991 | chr11 | 19372271 | 19734881 | + | NAV2:NM_001111018 | |
| hsa-mir-483_2 | chr11_2160204_-_4662 | chr11 | 2154904 | 2160204 | - | IGF2:uc001lvg | |
| hsa-mir-483_3 | chr11_2162354_-_4662 | chr11 | 2160204 | 2162354 | - | IGF2:uc021qcb | |
| hsa-mir-483_4 | chr11_2170833_-_4662 | chr11 | 2162354 | 2170833 | - | IGF2:NM_001007139 | |
| hsa-mir-483_1 | chr11_2182439_-_4662 | chr11 | 2170833 | 2182439 | - | INS-IGF2:NM_001042376 | |
| hsa-mir-4487_2 | chr11_47377001_+_4072 | chr11 | 47377001 | 47430046 | + | SLC39A13:uc001nfd | |
| hsa-mir-4696_4 | chr11_74442186_-_5260 | chr11 | 74430348 | 74442186 | - | | |
| hsa-mir-4300_1 | chr11_82114955_-_5290 | chr11 | 81590896 | 82114955 | - | | |
| hsa-mir-5691_2 | chr11_9113150_-_4751 | chr11 | 9072257 | 9113150 | - | SCUBE2:NM_001170690 | |
| hsa-mir-1304_1 | chr11_93469014_-_5319 | chr11 | 93463447 | 93469014 | - | | |
| hsa-mir-1244-2_4 | chr12_11802788_+_5610 | chr12 | 11802788 | 12022358 | + | | |
| hsa-mir-1244-2_3 | chr12_12022358_+_5610 | chr12 | 12022358 | 12202798 | + | | |
| hsa-mir-1244-2_2 | chr12_12202798_+_5610 | chr12 | 12202798 | 12224402 | + | | |
| hsa-mir-1244-2_1 | chr12_12232233_+_5610 | chr12 | 12232233 | 12363947 | + | | |
| hsa-mir-3612_1 | chr12_128751948_+_6143 | chr12 | 128751948 | 129192460 | + | TMEM132C:NM_001136103 | |
| hsa-mir-4701_1 | chr12_49168561_-_6409 | chr12 | 49164549 | 49168561 | - | | |
| hsa-mir-4701_2 | chr12_49177877_-_6409 | chr12 | 49168561 | 49177877 | - | | |
| hsa-mir-4701_3 | chr12_49182820_-_6409 | chr12 | 49177877 | 49182820 | - | | |
| hsa-mir-492_1 | chr12_95228174_+_5960 | chr12 | 95228174 | 95228804 | + | | |
| hsa-mir-2681_hsa-mir-4705_2 | chr13_102963036_-_7430 | chr13 | 102568995 | 102963036 | - | | |
| hsa-mir-2681_hsa-mir-4705_3 | chr13_102970054_-_7430 | chr13 | 102963036 | 102970054 | - | | |
| hsa-mir-2681_hsa-mir-4705_4 | chr13_103019749_-_7430 | chr13 | 102970054 | 103019749 | - | | |
| hsa-mir-2681_hsa-mir-4705_5 | chr13_103046869_-_7430 | chr13 | 103019749 | 103046869 | - | | |
| hsa-mir-2681_hsa-mir-4705_6 | chr13_103054778_-_7430 | chr13 | 103046869 | 103054778 | - | | |
| hsa-mir-4501_2 | chr13_96743093_+_7086 | chr13 | 96743093 | 96821806 | + | | |
| hsa-mir-4501_1 | chr13_96821806_+_7086 | chr13 | 96821806 | 97491816 | + | | |
| hsa-mir-1247_1 | chr14_102026759_-_8339 | chr14 | 102022013 | 102026759 | - | | |
| hsa-mir-208a_1 | chr14_23877486_-_8000 | chr14 | 23851199 | 23877486 | - | MYH6:NM_002471 | |
| hsa-mir-208b_1 | chr14_23904895_-_8001 | chr14 | 23881947 | 23904895 | - | MYH7:uc001wjx | |
| hsa-mir-3171_1 | chr14_28142427_-_8026 | chr14 | 27791032 | 28142427 | - | | |
| hsa-mir-4504_1 | chr14_50778947_-_8079 | chr14 | 50704285 | 50778947 | - | L2HGDH:NM_024884 | |
| hsa-mir-5586_2 | chr14_60194431_-_8117 | chr14 | 60097532 | 60194431 | - | RTN1:uc001xem | |
| hsa-mir-211_1 | chr15_31360295_-_9244 | chr15 | 31293264 | 31360295 | - | TRPM1:uc010azy | |
| hsa-mir-211_2 | chr15_31393929_-_9244 | chr15 | 31360295 | 31393929 | - | TRPM1:NM_001252024 | |
| hsa-mir-211_3 | chr15_31453476_-_9244 | chr15 | 31393929 | 31453476 | - | TRPM1:NM_001252020 | |
| hsa-mir-1233-2_1 | chr15_34828222_-_9262 | chr15 | 34817484 | 34828222 | - | | |
| hsa-mir-4310_2 | chr15_42186275_-_9299 | chr15 | 42144227 | 42186275 | - | SPTBN5:NM_016642 | |
| hsa-mir-190a_4 | chr15_62939510_+_8735 | chr15 | 62939510 | 63032644 | + | TLN2:NM_015059 | AC103740 |

| | | | | | | | |
|---|---|---|---|---|---|---|---|
| hsa-mir-190a_3 | chr15_63032644_+_8735 | chr15 | 63032644 | 63050789 | + | TLN2:uc002alc | AC103740 |
| hsa-mir-190a_2 | chr15_63050789_+_8735 | chr15 | 63050789 | 63112685 | + | TLN2:uc002ald | AC103740 |
| hsa-mir-190a_1 | chr15_63112685_+_8735 | chr15 | 63112685 | 63136829 | + | TLN2:uc010uic | |
| hsa-mir-4512_2 | chr15_66790153_-_9442 | chr15 | 66784406 | 66790153 | - | SNAPC5:NM_006049 | |
| hsa-mir-9-3_1 | chr15_89911330_+_8918 | chr15 | 89911330 | 89921273 | + | | |
| hsa-mir-484_5 | chr16_15737124_+_9915 | chr16 | 15737124 | 15744083 | + | NDE1:NM_001143979 | |
| hsa-mir-548w_2 | chr16_25703347_+_9984 | chr16 | 25703347 | 26040233 | + | | |
| hsa-mir-6510_1 | chr17_39675270_-_12066 | chr17 | 39669997 | 39675270 | - | KRT15:uc002hxa | |
| hsa-mir-1-2_hsa-mir-133a-1_1 | chr18_19411367_-_12752 | chr18 | 19405448 | 19411367 | - | | |
| hsa-mir-6718_1 | chr18_3878180_+_12447 | chr18 | 3878180 | 3897069 | + | | |
| hsa-mir-4528_3 | chr18_49866542_+_12575 | chr18 | 49866542 | 50278424 | + | DCC:NM_005215 | AC090660 |
| hsa-mir-4528_2 | chr18_50278424_+_12575 | chr18 | 50278424 | 50461142 | + | DCC:uc010xdr | AC090660 |
| hsa-mir-4528_1 | chr18_50461142_+_12575 | chr18 | 50461142 | 51062273 | + | DCC:uc010dpf | AC090660 |
| hsa-mir-4529_1 | chr18_53119764_+_12581 | chr18 | 53119764 | 53150171 | + | | |
| hsa-mir-3189_1 | chr19_18496770_+_13183 | chr19 | 18496770 | 18499987 | + | | |
| hsa-mir-5196_1 | chr19_35828658_+_13284 | chr19 | 35828658 | 35838264 | + | | |
| hsa-mir-642a_1 | chr19_46171502_+_13456 | chr19 | 46171502 | 46186982 | + | | |
| hsa-mir-4747_2 | chr19_4903092_+_12985 | chr19 | 4903092 | 4909510 | + | UHRF1:NM_001290050 | |
| hsa-mir-4747_1 | chr19_4909510_+_12985 | chr19 | 4909510 | 4962165 | + | UHRF1:NM_013282 | |
| hsa-mir-4324_1 | chr19_49828474_-_14360 | chr19 | 49792892 | 49828474 | - | SLC6A16:NM_014037 | |
| hsa-mir-4772_1 | chr2_103035250_+_14934 | chr2 | 103035250 | 103069024 | + | IL18RAP:uc010fiz | |
| hsa-mir-3681_1 | chr2_12147254_+_14587 | chr2 | 12147254 | 12718474 | + | | |
| hsa-mir-663b_2 | chr2_133015542_-_16037 | chr2 | 132919525 | 133015542 | - | | |
| hsa-mir-5590_1 | chr2_135596186_+_15097 | chr2 | 135596186 | 135659602 | + | | |
| hsa-mir-4773-2_1 | chr2_152235885_-_16068 | chr2 | 152194342 | 152235885 | - | | |
| hsa-mir-3131_1 | chr2_219925238_-_16282 | chr2 | 219919142 | 219925238 | - | IHH:NM_002181 | |
| hsa-mir-4439_4 | chr2_225907330_-_16306 | chr2 | 225811782 | 225907330 | - | | |
| hsa-mir-562_1 | chr2_232888987_+_15417 | chr2 | 232888987 | 233201687 | + | | |
| hsa-mir-4440_hsa-mir-4441_3 | chr2_240111773_-_16360 | chr2 | 240098259 | 240111773 | - | HDAC4:uc010fyy | AC017028 |
| hsa-mir-3126_1 | chr2_69240276_+_14784 | chr2 | 69240276 | 69476459 | + | | |
| hsa-mir-103a-2_1 | chr20_3869581_+_16428 | chr20 | 3869581 | 3910529 | + | PANK2:NM_024960 | |
| hsa-mir-1-1_hsa-mir-133a-2_1 | chr20_61147660_+_16736 | chr20 | 61147660 | 61167971 | + | MIR1-1HG:NM_001302812.2 | |
| hsa-mir-3196_1 | chr20_61867276_+_16748 | chr20 | 61867276 | 61871859 | + | | |
| hsa-mir-6070_1 | chr21_45078201_-_17440 | chr21 | 44949072 | 45078201 | - | HSF2BP:NM_007031 | |
| hsa-mir-5571_hsa-mir-650_2 | chr22_22985768_+_17555 | chr22 | 22985768 | 22988782 | + | GGTLC2:NM_001282879 | D86994 |
| hsa-mir-5571_hsa-mir-650_1 | chr22_22988782_+_17555 | chr22 | 22988782 | 23229960 | + | GGTLC2:NM_001282879 | D86994 |
| hsa-mir-885_2 | chr3_10452499_-_18883 | chr3 | 10377987 | 10452499 | - | | |
| hsa-mir-885_3 | chr3_10547341_-_18883 | chr3 | 10452499 | 10547341 | - | | |
| hsa-mir-885_4 | chr3_10661646_-_18883 | chr3 | 10547341 | 10661646 | - | | |
| hsa-mir-885_5 | chr3_10668492_-_18883 | chr3 | 10661646 | 10668492 | - | | |
| hsa-mir-885_6 | chr3_10749716_-_18883 | chr3 | 10668492 | 10749716 | - | | |
| hsa-mir-5002_hsa-mir-6083_5 | chr3_123813528_+_18553 | chr3 | 123813528 | 124103672 | + | KALRN:NM_003947 | |
| hsa-mir-3919_2 | chr3_158991036_+_18705 | chr3 | 158991036 | 159481930 | + | SCHIP1:NM_001197108 | |
| hsa-mir-551b_2 | chr3_167967310_+_18723 | chr3 | 167967310 | 168530083 | + | | |
| hsa-mir-944_2 | chr3_189507449_+_18807 | chr3 | 189507449 | 189526061 | + | TP63:NM_001114980 | |
| hsa-mir-944_1 | chr3_189526061_+_18807 | chr3 | 189526061 | 189615068 | + | TP63:uc003fse | |
| hsa-mir-2115_1 | chr3_48369831_-_19028 | chr3 | 48348336 | 48369831 | - | SPINK8:uc003csq | |
| hsa-mir-3938_3 | chr3_55984588_-_19117 | chr3 | 55693497 | 55984588 | - | | |
| hsa-mir-3938_4 | chr3_56115012_-_19117 | chr3 | 55984588 | 56115012 | - | | |
| hsa-mir-3921_2 | chr3_99833357_-_19191 | chr3 | 99595046 | 99833357 | - | | |
| hsa-mir-4453_1 | chr4_153457416_+_20048 | chr4 | 153457416 | 153460415 | + | | |
| hsa-mir-3945_1 | chr4_185776806_-_20661 | chr4 | 185768200 | 185776806 | - | | |
| hsa-mir-378d-1_1 | chr4_5991374_-_20190 | chr4 | 5899318 | 5991374 | - | | |
| hsa-mir-4274_hsa-mir-4798_2 | chr4_7194374_+_19637 | chr4 | 7194374 | 7383625 | + | SORCS2:NM_020777 | |
| hsa-mir-4274_1 | chr4_7383625_+_19637 | chr4 | 7383625 | 7744564 | + | SORCS2:uc011bwi | |
| hsa-mir-95_2 | chr4_8022552_-_20203 | chr4 | 7985159 | 8022552 | - | | |
| hsa-mir-95_3 | chr4_8073743_-_20203 | chr4 | 8022552 | 8073743 | - | | |
| hsa-mir-548f-3_2 | chr5_109961298_-_21600 | chr5 | 109765804 | 109961298 | - | TMEM232:uc003kow | |
| hsa-mir-1289-2_2 | chr5_132948223_-_21659 | chr5 | 132561617 | 132948223 | - | | |
| hsa-mir-874_1 | chr5_137008616_-_21684 | chr5 | 136953189 | 137008616 | - | KLHL3:uc011cyc | |
| hsa-mir-874_2 | chr5_137028374_-_21684 | chr5 | 137008616 | 137028374 | - | KLHL3:uc003lbs | |
| hsa-mir-874_3 | chr5_137042116_-_21684 | chr5 | 137028374 | 137042116 | - | KLHL3:NM_001257195 | |
| hsa-mir-874_4 | chr5_137056999_-_21684 | chr5 | 137042116 | 137056999 | - | KLHL3:NM_001257194 | |
| hsa-mir-584_3 | chr5_148442737_-_21749 | chr5 | 148421180 | 148442737 | - | SH3TC2:NM_024577 | |
| hsa-mir-6499_1 | chr5_150911531_-_21771 | chr5 | 150883653 | 150911531 | - | | |
| hsa-mir-6499_2 | chr5_150948505_-_21771 | chr5 | 150911531 | 150948505 | - | | |
| hsa-mir-4281_1 | chr5_176057562_-_21856 | chr5 | 176047085 | 176057562 | - | SNCB:NM_001318036 | |
| hsa-mir-3650_2 | chr5_38595507_-_21407 | chr5 | 38556748 | 38595507 | - | LIFR:uc003jli | |
| hsa-mir-449a_hsa-mir-449b_hsa-mir-449c_1 | chr5_54469005_-_21449 | chr5 | 54408799 | 54469005 | - | | |
| hsa-mir-583_1 | chr5_95297705_+_20939 | chr5 | 95297705 | 95966789 | + | | |
| hsa-mir-548a-1_1 | chr6_18522978_+_21990 | chr6 | 18522978 | 18723129 | + | | |
| hsa-mir-4463_1 | chr6_76092902_+_22489 | chr6 | 76092902 | 76168615 | + | | |
| hsa-mir-3666_5 | chr7_113928074_+_24057 | chr7 | 113928074 | 114055052 | + | FOXP2:uc003vgv | AC020606 |
| hsa-mir-3666_4 | chr7_114055052_+_24057 | chr7 | 114055052 | 114066557 | + | FOXP2:NM_001172766 | AC020606 |
| hsa-mir-3666_3 | chr7_114066557_+_24057 | chr7 | 114066557 | 114268595 | + | FOXP2:NM_001172767 | AC020606 |
| hsa-mir-3666_2 | chr7_114268595_+_24057 | chr7 | 114268595 | 114298091 | + | FOXP2:uc010ljz | AC020606 |
| hsa-mir-592_1 | chr7_126752374_-_24767 | chr7 | 126341811 | 126752374 | - | | |

| | | | | | | | | |
|---|---|---|---|---|---|---|---|---|
| hsa-mir-592_2 | chr7_126883569_-_24767 | chr7 | 126752374 | 126883569 | - | | |
| hsa-mir-592_3 | chr7_126892428_-_24767 | chr7 | 126883569 | 126892428 | - | | |
| hsa-mir-3907_1 | chr7_151133411_-_24916 | chr7 | 151129265 | 151133411 | - | | |
| hsa-mir-3907_2 | chr7_151137899_-_24916 | chr7 | 151133411 | 151137899 | - | | |
| hsa-mir-5707_1 | chr7_158383321_+_24283 | chr7 | 158383321 | 158384625 | + | | |
| hsa-mir-3943_1 | chr7_43157495_+_23766 | chr7 | 43157495 | 43202786 | + | | |
| hsa-mir-4649_1 | chr7_44143960_+_23773 | chr7 | 44143960 | 44154164 | + | | |
| hsa-mir-489_hsa-mir-653_1 | chr7_93116319_-_24623 | chr7 | 93053799 | 93116319 | - | | |
| hsa-mir-489_hsa-mir-653_2 | chr7_93189122_-_24623 | chr7 | 93116319 | 93189122 | - | | |
| hsa-mir-1273a_1 | chr8_101054195_-_25742 | chr8 | 100973276 | 101054195 | - | RGS22:uc022azf | |
| hsa-mir-1273a_2 | chr8_101083781_-_25742 | chr8 | 101054195 | 101083781 | - | RGS22:NM_001286693 | |
| hsa-mir-1273a_3 | chr8_101092583_-_25742 | chr8 | 101083781 | 101092583 | - | RGS22:uc022azh | |
| hsa-mir-1273a_4 | chr8_101118344_-_25742 | chr8 | 101092583 | 101118344 | - | RGS22:uc003yjc | |
| hsa-mir-383_3 | chr8_15095792_-_25474 | chr8 | 14412435 | 15095792 | - | SGCZ:NM_001322879 | |
| hsa-mir-4287_2 | chr8_27850369_-_25521 | chr8 | 27845147 | 27850369 | - | SCARA5:NM_173833 | |
| hsa-mir-2052_1 | chr8_75512101_+_25197 | chr8 | 75512101 | 75670587 | + | | |
| hsa-mir-4661_2 | chr8_92114847_+_25233 | chr8 | 92114847 | 92130604 | + | LRRC69:NM_001129890 | |
| hsa-mir-4661_1 | chr8_92130604_+_25233 | chr8 | 92130604 | 92221722 | + | LRRC69:uc003yew | |
| hsa-mir-3150a_2 | chr8_95907996_+_25242 | chr8 | 95907996 | 95961808 | + | NDUFAF6:NM_001354522 | |
| hsa-mir-3150b_1 | chr8_96085148_-_25726 | chr8 | 96079037 | 96085148 | - | | |
| hsa-mir-548aw_2 | chr9_135820932_+_26413 | chr9 | 135820932 | 135853894 | + | | |
| hsa-mir-3689a_hsa-mir-3689b_hsa-mir-3689c_hsa-mir-3689d-1_hsa-mir-3689d-2_hsa-mir-3689e_hsa-mir-3689f_1 | chr9_137764464_-_27058 | chr9 | 137711261 | 137764464 | - | ENST00000581079 | AL603650 |
| hsa-mir-6722_1 | chr9_139642980_-_27082 | chr9 | 139637414 | 139642980 | - | LCN6:uc004ciy | |
| hsa-mir-873_hsa-mir-876_3 | chr9_29212319_-_26571 | chr9 | 28719303 | 29212319 | - | LINGO2:NM_001258282 | |
| hsa-mir-4476_hsa-mir-4540_1 | chr9_37002773_-_26634 | chr9 | 36838531 | 37002773 | - | PAX5:uc011lpt | |
| hsa-mir-4476_hsa-mir-4540_2 | chr9_37026572_-_26634 | chr9 | 37002773 | 37026572 | - | PAX5:uc011lqd | |
| hsa-mir-204_1 | chr9_73461507_-_26724 | chr9 | 73149966 | 73461507 | - | TRPM3:uc004ahu | |
| hsa-mir-3153_1 | chr9_91926110_+_26184 | chr9 | 91926110 | 91931618 | + | | |
| hsa-mir-4290_1 | chr9_92803781_-_26801 | chr9 | 92782936 | 92803781 | - | | |
| hsa-mir-3910-2_1 | chr9_94712444_-_26808 | chr9 | 94325373 | 94712444 | - | ROR2:NM_004560 | |
| hsa-mir-4670_3 | chr9_95298374_-_26816 | chr9 | 95285175 | 95298374 | - | | |
| hsa-mir-934_1 | chrX_135614311_+_27651 | chrX | 135614311 | 135638966 | + | VGLL1:NM_016267 | |
| hsa-mir-6086_2 | chrX_13587694_+_27178 | chrX | 13587694 | 13614355 | + | | |
| hsa-mir-509-2_hsa-mir-509-3_1 | chrX_146341244_-_28195 | chrX | 146340278 | 146341244 | - | | |
| hsa-mir-105-1_2 | chrX_151561735_-_28211 | chrX | 151419148 | 151561735 | - | | |
| hsa-mir-105-1_hsa-mir-105-2_hsa-mir-767_3 | chrX_151619831_-_28211 | chrX | 151561735 | 151619831 | - | | |
| hsa-mir-1184-1_2 | chrX_154134848_-_28251 | chrX | 154114577 | 154134848 | - | | |
| hsa-mir-1184-1_3 | chrX_154250998_-_28251 | chrX | 154134848 | 154250998 | - | | |
| hsa-mir-1184-1_4 | chrX_154255215_-_28251 | chrX | 154250998 | 154255215 | - | | |
| hsa-mir-23c_1 | chrX_20074909_-_27831 | chrX | 20024831 | 20074909 | - | MAP7D2:uc004czq | |
| hsa-mir-23c_2 | chrX_20082923_-_27831 | chrX | 20074909 | 20082923 | - | MAP7D2:uc004czs | |
| hsa-mir-3915_hsa-mir-548f-5_8 | chrX_32834757_-_27856 | chrX | 32430371 | 32834757 | - | | |
| hsa-mir-3915_hsa-mir-548f-5_9 | chrX_32867937_-_27856 | chrX | 32834757 | 32867937 | - | | |
| hsa-mir-3915_hsa-mir-548f-5_10 | chrX_33146545_-_27856 | chrX | 32867937 | 33146545 | - | | |
| hsa-mir-3915_hsa-mir-548f-5_11 | chrX_33229673_-_27856 | chrX | 33146545 | 33229673 | - | | |
| hsa-mir-3915_hsa-mir-548f-5_12 | chrX_33357726_-_27856 | chrX | 33229673 | 33357726 | - | | |
| hsa-mir-325_hsa-mir-384_1 | chrX_76234957_-_28004 | chrX | 75878198 | 76234957 | - | | |
| hsa-mir-3690-2_2 | chrY_1337693_+_28265 | chrY | 1337693 | 1351571 | + | CSF2RA:NM_001161531 | BX649553 |
| hsa-mir-3690-2_1 | chrY_1351571_+_28265 | chrY | 1351571 | 1378828 | + | CSF2RA:NM_001161530 | BX649553 |
